# Proton-Induced Bystander Signaling Drives Cell-Type-Specific Proteomic Remodeling in Chondrocytes and Endothelial Cells

**DOI:** 10.3390/cancers18213534

**Published:** 2026-11-03

**Authors:** Mihaela Tudor, Benoît Bernay, Mihaela Temelie, Liviu Craciun, François Chevalier, Diana Iulia Savu

**Affiliations:** 1 Department of Life and Environmental Physics, Horia Hulubei National Institute for R&D in Physics and Nuclear Engineering, 077125 Magurele, Romania; mihaela.tudor@nipne.ro (M.T.); mihaela.temelie@nipne.ro (M.T.); 2 Faculty of Biology, University of Bucharest, Splaiul Independentei 91-95, 050095 Bucharest, Romania; 3 US EMerode, Plateform Proteogen Université de Caen Normandie, 14032 Caen, France; benoit.bernay@unicaen.fr; 4 Radiopharmaceutical Research Centre, Horia Hulubei National Institute for R&D in Physics and Nuclear Engineering, 077125 Magurele, Romania; cliviu@nipne.ro; 5 Centre de recherche sur les Ions les Matériaux et la Photonique, Université de Caen Normandie, CEA, CNRS, ENSICAEN, UMR6252 CIMAP, Team Applications in Radiobiology with Accelerated Ions, 14000 Caen, France

**Keywords:** proton therapy, bystander effects, chondrosarcoma, proteomics

## Abstract

Background/Objectives: Radiation-induced bystander effects (RIBEs), whereby irradiated cells transmit biological signals to neighboring, non-irradiated cells, are a well-recognized phenomenon in radiobiology. However, proton-induced RIBEs remain incompletely understood. This study investigated the bystander responses elicited by proton-irradiated chondrosarcoma cells in non-irradiated chondrocytes and endothelial cells. Methods: T/C-28A2 chondrocytes and EA.hy926 endothelial cells were exposed to conditioned medium (CM) collected from SW1353 chondrosarcoma cells irradiated with protons at doses ranging from 0.1 to 2 Gy. Clonogenic survival and micronucleus (MN) assays were initially performed to assess the susceptibility of recipient cells to bystander signaling. Based on these findings, cell cycle distribution, reactive oxygen species (ROS) level, and proteomic profiles were subsequently analyzed under the most responsive experimental conditions. Results: CM from proton-irradiated chondrosarcoma cells induced significant but cell-type-specific bystander responses. Chondrocytes were most responsive to CM from cells irradiated with 0.1 Gy, exhibiting reduced clonogenic survival, increased micronucleus formation, and adaptive-like proteomic changes consistent with reduced proliferation and extracellular matrix remodeling. In contrast, endothelial cells responded predominantly to conditioned medium from 2 Gy irradiated cells, displaying reduced survival, increased genomic instability, cell cycle perturbations, persistent ROS accumulation, and extensive proteomic remodeling involving elevated levels of proteins associated with metabolism and DNA repair proteins. Network analysis further revealed cell-type-specific alterations in inflammatory, mitochondrial, and intracellular signaling pathways, highlighting distinct mechanisms underlying bystander response. Conclusions: Proton-induced bystander responses differed between the two recipient cell models and were associated with distinct proteomic profiles under the selected experimental conditions. These findings identify candidate proteins and pathways for further investigation but do not establish causal mechanisms or validated biomarkers. Further studies are needed to distinguish the effects of recipient cell type from those of donor irradiation dose and to determine the relevance of these responses to primary healthy tissues.

## Simple Summary

Proton therapy is a precise form of radiotherapy, but irradiated tumor cells may also influence neighboring non-irradiated cells through radiation-induced bystander effects. In this study, we investigated how conditioned medium from proton-irradiated chondrosarcoma cells affected two recipient cell models: T/C-28A2 chondrocytes and EA.hy926 endothelial cells. The responses differed between the models and depended on the irradiation dose delivered to the tumor cells. Chondrocytes showed more pronounced functional changes following exposure to conditioned medium from cells irradiated at 0.1 Gy, whereas endothelial cells responded more strongly to conditioned medium from cells irradiated at 2 Gy. Proteomic analysis revealed distinct patterns of protein abundance changes in the two models, providing potential leads for further investigation of the molecular processes associated with bystander signaling. Because established cell lines were used and the detailed molecular analyses involved different donor irradiation doses, the findings require further validation before conclusions can be drawn about effects on healthy tissues or their clinical implications.

## 1. Introduction

According to the classical theory of ionizing radiation action, cellular effects are primarily mediated through DNA damage that ultimately leads to cell death. However, this paradigm has evolved over the past two decades, as numerous studies have demonstrated that non-targeted effects can significantly influence a large population of cells, particularly at low radiation doses where biological responses deviate from a linear dose–response relationship [1]. Deciphering these mechanisms is therefore essential for refining therapeutic strategies and predicting clinical outcomes.

RIBEs occur when non-irradiated cells respond to signals from irradiated neighbors, mediated through gap junctions or soluble factors in the extracellular medium [2,3]. While previous studies noted such responses in chondrosarcoma models [4,5,6,7] the specific impact of high-linear energy transfer (LET) particles, such as protons, remains a critical area of investigation.

In the context of hadrontherapy, which employs high-LET particles such as protons and carbon ions, bystander effects exhibit distinctive characteristics [8]. Proton therapy achieves superior precision by depositing the majority of its energy within the tumor via the Bragg peak, with minimal exit dose, thereby reducing the integral dose to surrounding healthy tissues [9]. Advanced techniques, including intensity-modulated and volumetric modulated arc therapy, combined with innovations like proton computed tomography (pCT) for range verification, have further enabled precise dose sparing of critical regions. These advances are particularly valuable in treating pediatric and young adult cancers, where managing long-term risks of tissue damage and secondary malignancies is paramount [10]. However, despite this ballistic precision, proton-induced bystander responses—triggered by ROS and cytokine release—display spatial and temporal dynamics that differ significantly from photon-based therapies [11,12,13]. Similarly, high-LET carbon ions generate complex DNA lesions and MN in bystander cells, offering potential therapeutic advantages but requiring careful management to minimize toxicity in normal tissues [11,12,14]. High-LET particles generate complex DNA lesions and micronuclei in non-irradiated cells, orchestrated by key signaling pathways such as NF-κB-mediated inflammation, MAPK, and JAK/STAT [15,16].

The vascular compartment is a primary target for high-LET radiation toxicity, which triggers distinct pathophysiologic responses compared to X-rays. In endothelial models, high-LET radiation activates genes linked to endothelial dysfunction and ROS-driven inflammatory cycles. These molecular shifts drive a cascade of senescence, vascular rarefaction, and chronic inflammation, ultimately leading to progressive tissue ischemia and severe organ-specific pathologies, such as leukoencephalopathy, late enteropathy, and myocardial fibrosis [17,18].

Despite the increasing interest in RIBEs, important questions remain regarding how the recipient cell type influences the biological and molecular responses to signals released by irradiated tumor cells. This question is particularly relevant in the context of chondrosarcoma, where tumor cells interact with surrounding cartilage-associated cells and the vascular compartment. Although these cellular populations have distinct biological functions, their responses to tumor-derived signals following proton irradiation have not been fully characterized in a comparative experimental setting.

In the present study, T/C-28A2 chondrocytes and EA.hy926 endothelial cells were selected as complementary in vitro recipient models to investigate potential differences in responses to CM obtained from proton-irradiated SW1353 chondrosarcoma cells. The T/C-28A2 cells provide a model for studying chondrocyte-related responses [6,19,20,21], while EA.hy926 cells allow investigation of responses in an endothelial-like cellular model [22].

Another important question is whether the responses observed at the functional level are accompanied by distinct molecular changes. Although clonogenic survival, micronucleus formation, cell cycle distribution, and ROS measurements provide information about cellular outcomes, these endpoints do not independently identify the broader protein-level changes associated with the responses. Quantitative proteomics can complement these functional measurements by identifying differentially abundant proteins and associated biological pathways. Accordingly, this study combines functional assays with proteomic profiling to characterize the responses of two recipient cell models to tumor-derived signals following proton irradiation and to identify molecular candidates for future validation.

## 2. Materials and Methods

### 2.1. Cell Culture

Two “normal” human cell lines were employed: the T/C-28A2 chondrocyte line, kindly provided by collaborators from the CIMAP laboratory (Caen, France), and originating from Goldring’s laboratory (Massachusetts General Hospital, Charlestown, MA, USA), and EA.hy926 endothelial cells (ATCC, Manassas, VA, USA). T/C-28A2 is an immortalized human chondrocyte cell line [20], while EA.hy926 is a hybrid cell line generated by fusion of human umbilical vein endothelial cells (HUVECs) with the A549 lung carcinoma cell line [23]. Both cell lines were cultured in high-glucose DMEM supplemented with 10% fetal bovine serum (FBS) under standard conditions (37 °C, 5% CO_2_, 95% humidity). The SW1353 chondrosarcoma cell line (CLS Cell Lines Service GmbH, Eppelheim, Germany) was prepared as described previously [24].

For proton irradiation experiments, bystander cells were seeded one day prior to irradiation at densities of 30,000 cells per well in 12-well plates (1 mL medium) and 5000 cells on 10 mm glass coverslips (0.5 mL medium), achieving 90–100% confluence at the time of medium transfer.

CM from proton-irradiated chondrosarcoma cells was transferred to recipient non-irradiated cells 24 h post-irradiation.

### 2.2. Cell Irradiation

Chondrosarcoma SW1353 cells were irradiated with 0.1, 0.5 and 2 Gy of 10.6 MeV protons at a LET of 12.6 keV/μm, delivered by the Cyclotron facility at IFIN-HH (Măgurele, Romania), as described in Tudor et al. (2023, 2026) [24,25]. Cells were irradiated in 12- or 24-well plates (Techno Plastic Products, Trasadingen, Switzerland) with a normal-incidence proton beam (~10 pA, ~1 Gy/min). To optimize energy deposition, a methyl polymethacrylate (PMMA) filter was employed to place samples close to the Bragg peak. A computer-controlled moving axis ensured precise well-cassette positioning. To reduce proton flux by 6 orders of magnitude, the beam was extracted into air through a 25 μm tungsten foil and spread via successive scattering on metal sheets, with collimators selecting a small fraction of scattered protons. Setup validation using Gafchromic EBT2 films (Ashland Inc., Wilmington, DE, USA) showed <10% field non-uniformity across a 30 mm diameter. Fluka simulations confirmed beam spread and uniformity, while dose monitoring was performed using an Advanced Markus 34045 ionization chamber (PTW, Freiburg, Germany).

The selected doses (0.1, 0.5, and 2 Gy) were used to investigate the dependence of radiation-induced bystander signaling on the dose delivered to the donor chondrosarcoma cells. They span a low-to-higher experimental dose range previously investigated in studies of radiation-induced bystander effects in chondrocytes [5,6,21].

### 2.3. Medium Transfer

Twenty-four hours after irradiation, culture media from SW1353 cells were collected, sterile-filtered using 0.2 μm syringe filters at room temperature, and then applied to non-irradiated T/C-28A2 and EA.hy926 cultures. Following a 24 h incubation with CM, bystander cells were trypsinized, resuspended in fresh medium, and processed for further analyses.

### 2.4. Colony Formation Assay

Twenty-four hours after CM transfer, cells from each of the two duplicate wells per condition were trypsinized, counted twice, and plated in triplicate at densities of 800 cells/well (T/C-28A2) and 1600 cells/well (EA.hy926) in 6-well plates with 2 mL medium. Plates were incubated for 14 days under standard conditions, followed by staining with Crystal Violet. Colonies with ≥50 cells were scored.

Plating efficiency (PE) was calculated as the ratio of colonies formed to cells plated, multiplied by 100. Survival fraction (SF) was derived by dividing the number of colonies formed post-irradiation by the number of cells plated, normalized to PE. SF values were expressed relative to non-irradiated controls. Each experiment’s results represent the mean of at least three independent biological experiments, where technical triplicates from each duplicate were averaged within each run prior to statistical evaluation.

### 2.5. MN Assay

For bystander studies, cells were seeded on 10 mm glass slides at 1000 cells per slide approximately 48 h before CM transfer. After medium transfer, cytochalasin B (6 µg/mL) was added for ~22 h prior to fixation. Staining and scoring procedures follow the protocols detailed in Tudor et al. [25,26], where, after fixation and acridine orange staining, 1000 cells per coverslip (2 coverslips per sample) were analyzed by scoring micronuclei according to Fenech’s established morphological criteria [27].

### 2.6. Cell Cycle Analysis

Bystander cells were seeded at 20,000 cells per well about 48 h before the addition of CM. Analyses were performed at 3 and 24 h post-transfer to evaluate bystander effects. Cells were detached using 1× trypsin-EDTA, pelleted by centrifugation at 300× *g* for 5 min at room temperature, and fixed in cold 70% ethanol. Fixed samples were stored at −20 °C prior to staining. DNA was labeled with Hoechst 33342 (1 µg/mL) overnight at 4 °C and subsequently analyzed on a CytoFLEX flow cytometer (Beckman Coulter, Brea, CA, USA) using CytExpert software (Version 2.6.0.105, Beckman Coulter, Brea, CA, USA).

### 2.7. ROS Measurement

Bystander cells were plated at 40,000 cells per well 48 h prior to medium transfer. ROS levels were assessed at 3 and 24 h post-transfer using flow cytometry with MitoSOX^TM^ Red (M36008, Invitrogen^TM^, Thermo Fisher Scientific, Waltham, MA, USA) for mitochondrial superoxide and CM-H2DCFDA (C6827, Invitrogen^TM^, Thermo Fisher Scientific, Waltham, MA, USA) for total ROS detection. Cells were subsequently collected by trypsinization, centrifuged at 300× *g* for 5 min, and resuspended in Hank’s Balanced Salt Solution (HBSS) supplemented with 5 µM of the corresponding fluorescent probe. After 15 min of incubation at 37 °C, samples were analyzed using a CytoFLEX flow cytometer according to the manufacturer’s recommended settings.

### 2.8. Protein Extraction

Following the CM transfer described earlier, bystander cells were cultured for 24 h before being harvested for protein extraction.

Cells were lysed in RIPA buffer (comprising 25 mM Tris-Base, 120 mM NaCl, 10 mM Triton X, and 1 mM EDTA) at a concentration of 50 µL per million cells. Following two 20 min ice-incubation periods—separated by a 20 s mechanical homogenization step—samples were centrifuged (20,000× *g*, 15 min, 4 °C) to recover the supernatant. These extracts were stored at −20 °C until use. Protein concentration was measured using the Pierce^TM^ 660 nm Protein Assay Reagent (Thermo Scientific, Waltham, MA, USA), utilizing BSA for the standard curve. Data acquisition was performed on an FC Multiskan^TM^ (Thermo Scientific, Waltham, MA, USA) microplate reader equipped with SkanIt software (version RE 6.0.2, Thermo Scientific, Waltham, MA, USA).

Proteomic analysis was conducted by a TIMS-TOF Pro mass spectrometer (Bruker Daltonics, Bremen, Germany) coupled to a nanoElute UHPLC system. Peptides were separated on an Acclaim PepMap100 trap column (Thermo Scientific, Waltham, MA, USA) and an IonOpticks C18 column (25 cm × 75 µm) (IonOpticks, Collingwood, Victoria, Australia) maintained at 50 °C at a flow rate of 250 nl/min. The LC solvents consisted of 0.1% formic acid in water (solvent A) and 0.1% formic acid in acetonitrile (solvent B). The gradient was set as follows: 2–30% B in 70 min, 30–37% B in 5 min, followed by an 85% B wash (85 min total run time). One injection per sample (100 ng) was performed following the protocol described by Gilbert et al. [6].

In short, protein samples (5 µg) were processed for mass spectrometry through overnight modified GASP (gel-aided sample preparation), with cysteine alkylation by acrylamide at 37 °C, followed by peptide purification using µC18 Omix tips (Agilent Technologies, Santa Clara, CA, USA)). Nano-LC–MS/MS was operated in DIA mode to ensure systematic fragmentation of all detectable peptide ions across 16 variable *m*/*z* windows (400–1200 *m*/*z*), acquisition mode—diaPASEF. This systematic approach was selected over conventional DDA to enhance quantitative reproducibility and data completeness across all experimental conditions.

Data analysis was performed in DIA-NN (v. 1.6.0) utilizing a library-free workflow. Spectral matching was conducted against in silico-generated libraries (UniProt H. sapiens), with mass and RT corrections applied automatically. The quantification pipeline utilized fragment ion intensities (XIC), applying a 1% FDR at the precursor and protein-group levels. Only consistently quantified proteins across 2–3 biological replicates were retained for downstream analysis, with missing values addressed through imputation, with T/C-28A2 presenting 7583 protein groups quantified where 7301 were retained (6843 without missing values) and EA.hy926 presenting 9010 where 8619 were retained (6505 without missing values). Fixed modifications included C-propionamide, while N-terminal acetylation and methionine oxidation were treated as variable; post-translational modification-specific analysis was outside the scope of this study.

Functional enrichment was conducted based on the species-specific origin of the respective cell lines, with identified proteins mapped using the UniProt database. Venn diagrams (BioTools.fr) were employed to visualize cell line differentially abundant protein overlap among differentially abundant proteins. Gene Ontology (GO) analysis for biological processes was performed via the ClueGO plugin in Cytoscape Web version 1.1.0 [28], applying a medium specificity setting, a GO tree level range of 3–8, a minimum of 3 genes per term, a Kappa score threshold of 1, and 4% of the term, with a two-sided hypergeometric test, Bonferroni step-down, and whole genome as background. Statistical significance for GO terms was defined by a Bonferroni step-down (Holm)-adjusted *p* < 0.05 that corrects the number of terms tested, independently of the protein-level FDR used for differential abundance. Protein–protein interaction (PPI) landscapes were mapped using STRING to depict physical and functional associations. Colored nodes are query proteins and first shell of interactors, while white nodes are second shell of interactors, filled with a 3D structure that is known or predicted. Node color = corrected log2FC (blue decreased, red increased); diamond = detected in one condition only; node size = degree. Edges represent protein–protein associations with a minimum interaction combined score ≥ 0.4, with width proportional to score (thicker lines have a higher edge confidence), where edge thickness correlates with interaction strength [29,30,31]. STRING enrichment used an FDR lower than 0.05 from the whole genome. Central regulatory nodes within these networks were further identified using the CytoHubba plugin through the selection of the top 10 ranked by MCC.

### 2.9. Statistical Analysis

Experimental data were obtained from three independent biological experiments (*n* = 3), each conducted with duplicate technical replicates per condition, and are expressed as mean ± standard error of the mean (SEM). Statistical analyses were performed on the averaged values from these biological replicates. Clonogenic survival data were processed using SigmaPlot (version 15.0, Systat Software, Inc., San Jose, CA, USA) while other experimental outputs were analyzed via OriginPro (version 8.6, OriginLab Corporation, Northampton, MA, USA). One-Way Analysis of Variance (ANOVA) was used to assess dose-dependent responses within individual cell lines, whereas Two-Way ANOVA incorporating cell line and radiation dose as independent factors was applied only for the MN assay to compare biological responses across cell types and to evaluate potential factor interactions. Post hoc pairwise comparisons were carried out using Fisher’s LSD test to identify specific differences relative to untreated controls or between group conditions. The precise statistical test, sample sizes, and post hoc analyses applied are stated in each corresponding figure legend, with statistical significance defined as *p* < 0.05 (*), *p* < 0.01 (**), and *p* < 0.001 (***).

For proteomic data, we used a sample size of three biological replicates per group (sham-CM vs. irradiated-CM), 6 samples per cell line, 12 LC-MS/MS runs, and one injection per sample. Relative protein abundance was evaluated using the R (version 4.1/4.2) package DEP (version 1.16/1.18). Each cell line was analyzed separately, using three sham-CM replicates and three irradiated-CM replicates (0.1 Gy for T/C-28A2 and 2 Gy for EA.hy926); MaxLFQ intensities were log2-transformed, with no additional normalization. Only proteins identified in at least 2 out of 3 replicates per condition were retained for downstream analysis. Missing values were addressed through imputation using random draws from a manually defined, left-shifted Gaussian distribution. Differential expression was defined using the contrast between irradiated-CM and sham-CM. Fold changes were expressed as log2 fold change values (log_2FC = log_2(irradiated-CM/sham-CM)), where positive values indicate higher expression/abundance in bystander cells exposed to irradiated-CM compared to those exposed to sham-CM. Proteins exhibiting a q < 0.05 and |FC| ≥ 1.5, resulting in a |log_2_ FC| ≥ 0.585, were classified as significantly enriched. FDR corresponds to the DEP adjusted *p*-value, which represents the fdrtool q-value (empirical null). A sensitivity analysis was performed to evaluate the impact of alternative statistical approaches: applying Benjamini–Hochberg (BH) correction yielded 2 passing proteins for T/C-28A2 and 0 for EA.hy926; incorporating the biological replicate as a blocking factor to account for paired sham and irradiated samples within each replicate alongside BH correction resulted in 0 passing proteins for both cell lines, with the lowest adjusted *p*-values being 0.096 and 0.59, respectively. All proteomic datasets have been deposited in the iProX public repository under accession code IPX0019367001 (https://www.iprox.cn, accessed on 22 October 2026).

## 3. Results

To investigate the indirect effects of chondrosarcoma proton irradiation, we analyzed clonogenic survival, genotoxicity, oxidative stress, cell cycle progression and protein expression modulations in non-irradiated T/C-28A2 chondrocytes and EA.hy926 endothelial cells exposed to CM from irradiated SW1353 chondrosarcoma cells. Clonogenic survival and MN assays were first performed to identify whether bystander responses occurred and to determine the irradiation doses that elicited significant effects in each recipient cell type. Based on these results, representative experimental conditions were selected for further investigation of the cellular response/underlying mechanisms through cell cycle analysis, ROS quantification, and quantitative proteomic profiling. This stepwise approach enabled the characterization of both the biological outcomes and the molecular pathways associated with proton-induced bystander signaling.

### 3.1. Analysis of Cell Survival

**Figure 1:**
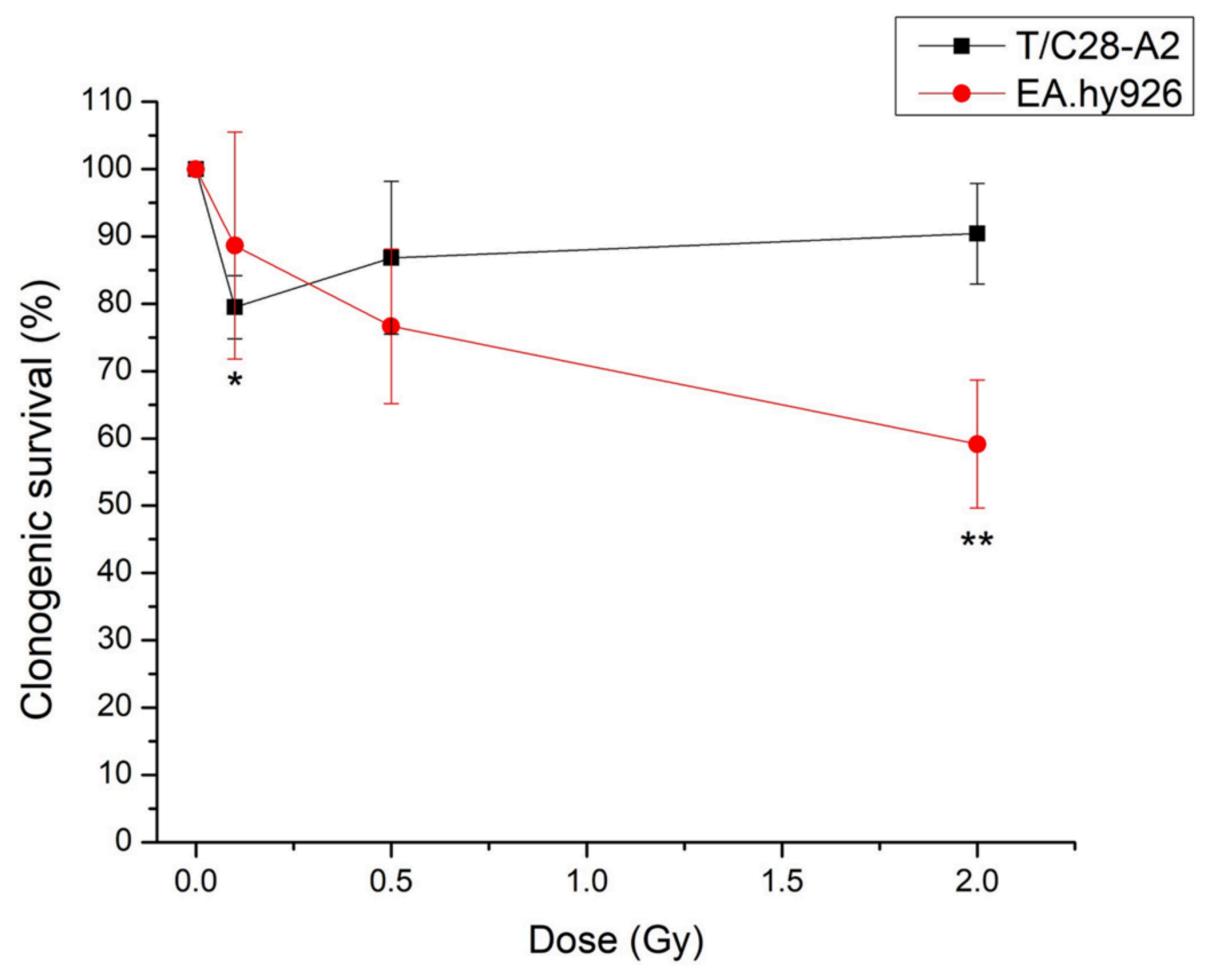
Clonogenic survival of T/C-28A2 chondrocytes and EA.hy926 endothelial cells following medium transfer from SW1353 chondrosarcoma cells exposed to proton irradiation (0–2 Gy). Values represent the mean of at least three independent experiments; errors are expressed as ± SEM (*n* = 3). Differences relative to unirradiated control (0 Gy) were evaluated per cell line the One-Way ANOVA method, * *p* < 0.05, ** *p* < 0.01.

In Figure 1 we can observe that the two recipient cell lines exhibited distinct dose-dependent responses following exposure to CM from irradiated SW1353 cells. In T/C-28-A2 chondrocytes, CM derived from cells irradiated with 0.1 Gy significantly reduced cell survival to 79 ± 4.7% compared with the control (*p* = 0.017). In contrast, EA.hy926 endothelial cells were more sensitive to CM obtained from SW1353 cells irradiated with 2 Gy, exhibiting a more pronounced reduction in survival, down to 59 ± 9.5% of the control (*p* = 0.0069). Furthermore, a statistically significant difference was observed between the two cell lines at both 0.1 Gy (*p* = 0.023) and 2 Gy (*p* < 0.001), highlighting cell-line-specific responses to radiation.

### 3.2. Investigation of DNA Damage by MN Assay

**Figure 2:**
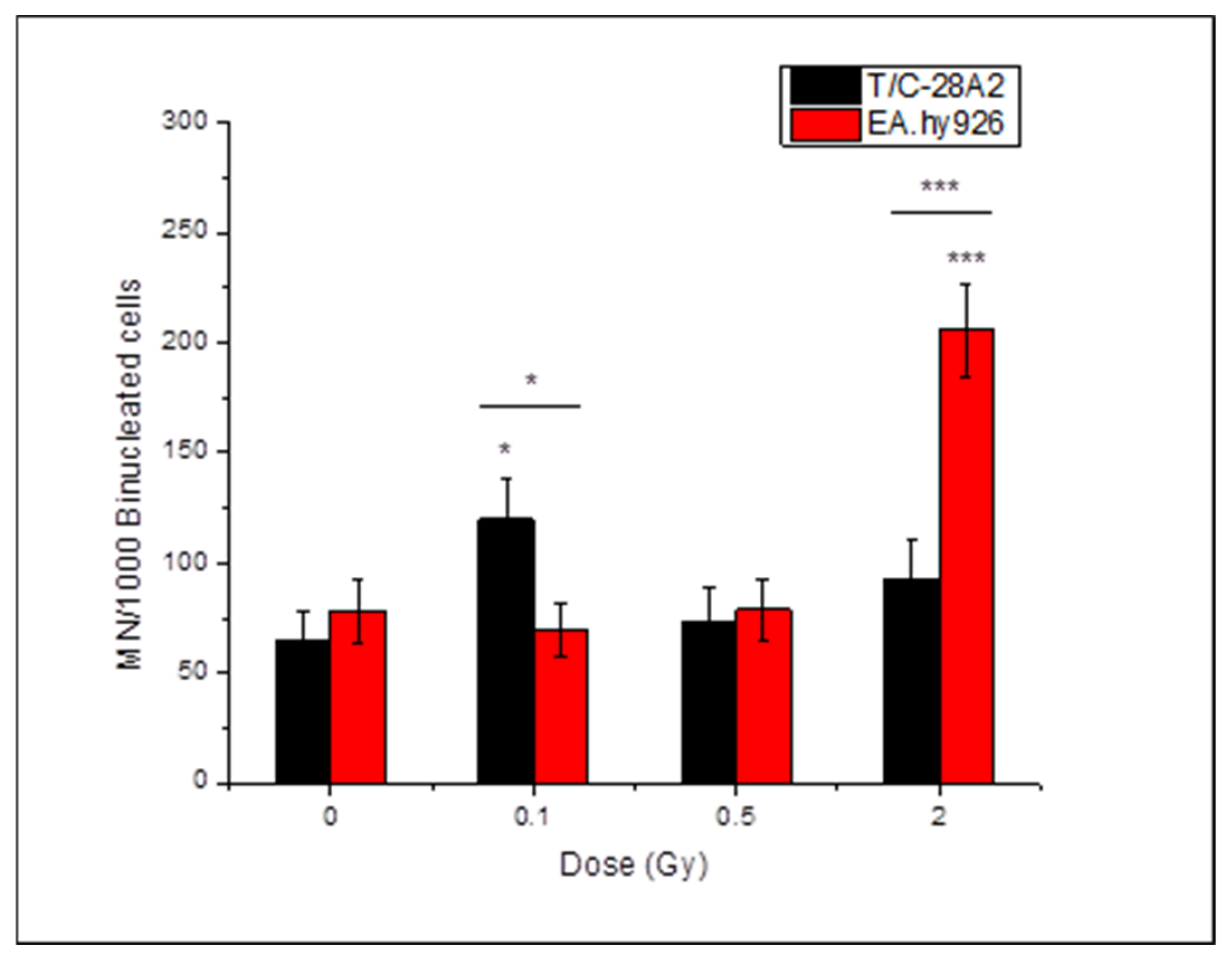
Genotoxicity induced in T/C-28A2 chondrocytes and EA.hy926 endothelial cells via medium transfer from SW1353 chondrosarcoma cells exposed to proton irradiation (0–2 Gy). Values represent the mean of at least three independent experiments; errors are expressed as ± SEM (*n* = 3). Statistical significance was evaluated using One-Way ANOVA to assess dose-dependent differences relative to the unirradiated control (0 Gy) within each cell line, and Two-Way ANOVA incorporating cell line and radiation dose as independent factors to evaluate differences and interactions between conditions. Pairwise comparisons were performed using Fisher’s LSD post hoc test. * *p* < 0.05, *** *p* < 0.001.

Both recipient cell lines elicited distinct dose-dependent responses post-exposure to CM (Figure 2). In chondrocytes, CM from chondrosarcoma cells irradiated with 0.1 Gy protons significantly increased MN formation, reaching an average of approximately 120 MN/1000 BN cells (*p* = 0.018) compared with the control. Higher doses (0.5 and 2 Gy) were associated with no alteration at the level of induced MN.

Endothelial cells displayed an opposite pattern. Exposure to CM from SW1353 cells irradiated with 2 Gy protons resulted in substantial increase in MN formation with an average of approximately 200 MN/BN cells (*p* < 0.001), No significant difference was observed at 0.5 Gy when comparing to control or between cell lines. Conversely, exposure to 0.5 Gy yielded no statistically significant difference relative to the control or between the cell lines.

Overall, these findings demonstrate that proton irradiation triggered distinct cell-type- and dose-dependent bystander survival and genotoxicity. Chondrocytes exhibited minimal survival accompanied by maximal DNA damage following exposure to CM generated after low-dose (0.1 Gy) proton irradiation, whereas endothelial cells were more susceptible to CM derived from cells irradiated with the highest proton dose (2 Gy).

Further, these doses will be used for chondrosarcoma irradiation.

### 3.3. Investigation of Cell Cycle Dynamics

To further investigate the cellular response, cell cycle dynamics were analyzed by flow cytometry to provide insight into phase-specific changes at 3 and 24 h following CM exposure of recipient cells.

**Figure 3:**
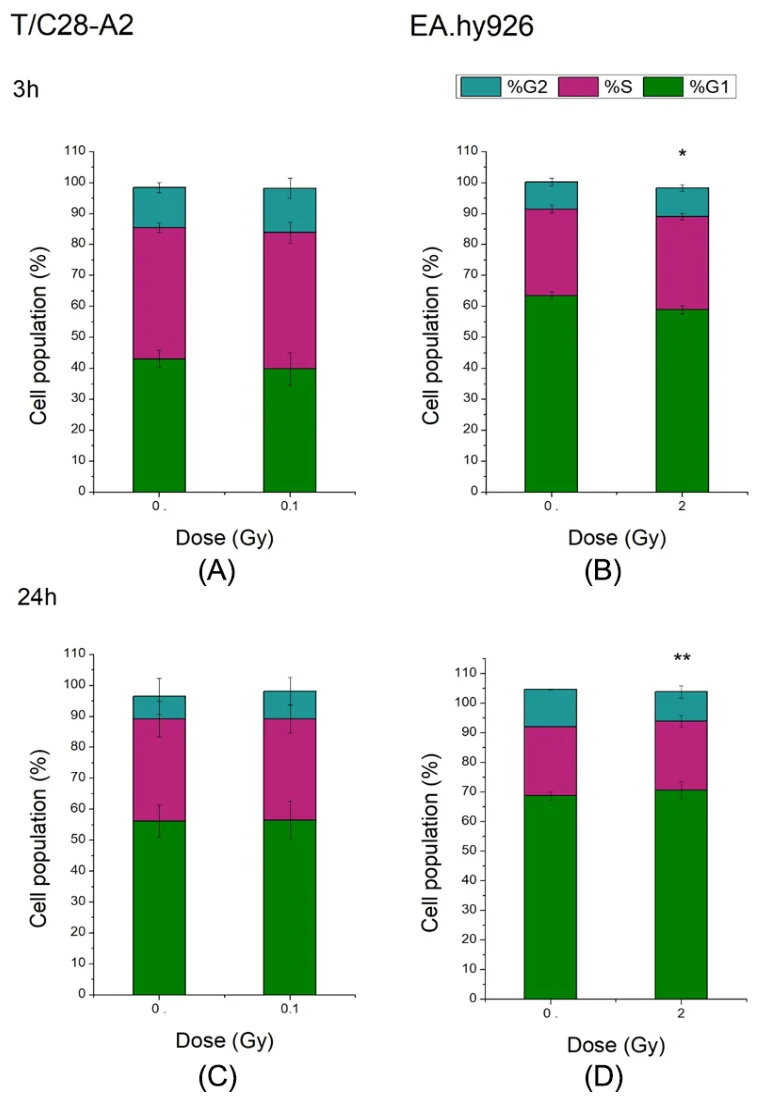
Quantification of cell cycle distribution in T/C-28A2 chondrocytes (**left panel**) and EA.hy926 endothelial cells (**right panel**) by flow cytometry at 3 (**A**,**B**) and 24 (**C**,**D**) h after transfer of CM from SW1353 chondrosarcoma cells exposed to 0.1 Gy and 2 Gy protons. Values represent the mean of at least three independent experiments; errors are expressed as ± SEM (*n* = 3). Statistical significance was calculated using the One-Way ANOVA method followed by Fisher’s LSD post hoc test, * *p* < 0.05, ** *p* < 0.01.

In the T/C-28A2 chondrocytes, no statistically significant alterations in cell cycle phase distribution were observed at either 3 or 24 h post-exposure to CM from 0.1 Gy proton-irradiated chondrosarcoma cells (Figure 3).

In contrast, EA.hy926 endothelial cells exhibited clear evidence of cell cycle disruption at 3 and 24 h post-exposure to CM from 2 Gy proton-irradiated chondrosarcoma cells (Figure 3). At 24 h, the G2-phase population decreased compared to control (*p* = 0.002), and at 3 h, a significant reduction was observed in the G1-phase population (*p* = 0.012). Additional time-dependent differences were also identified in the G1 and S phases for both treated and control groups, while G2-phase changes over time were observed only in the sham controls.

### 3.4. Quantification of Oxidative Stress and Reactive Oxygen Species Production

Accumulation of ROS, particularly when exceeding the cellular antioxidant defense capacity, can significantly affect cell viability and function. Both total and mitochondrial ROS levels were measured in chondrocytes and endothelial cells at 3 and 24 h after treatment with CM derived from SW1353 cells proton-irradiated with 0.1 Gy or 2 Gy, respectively.

**Figure 4:**
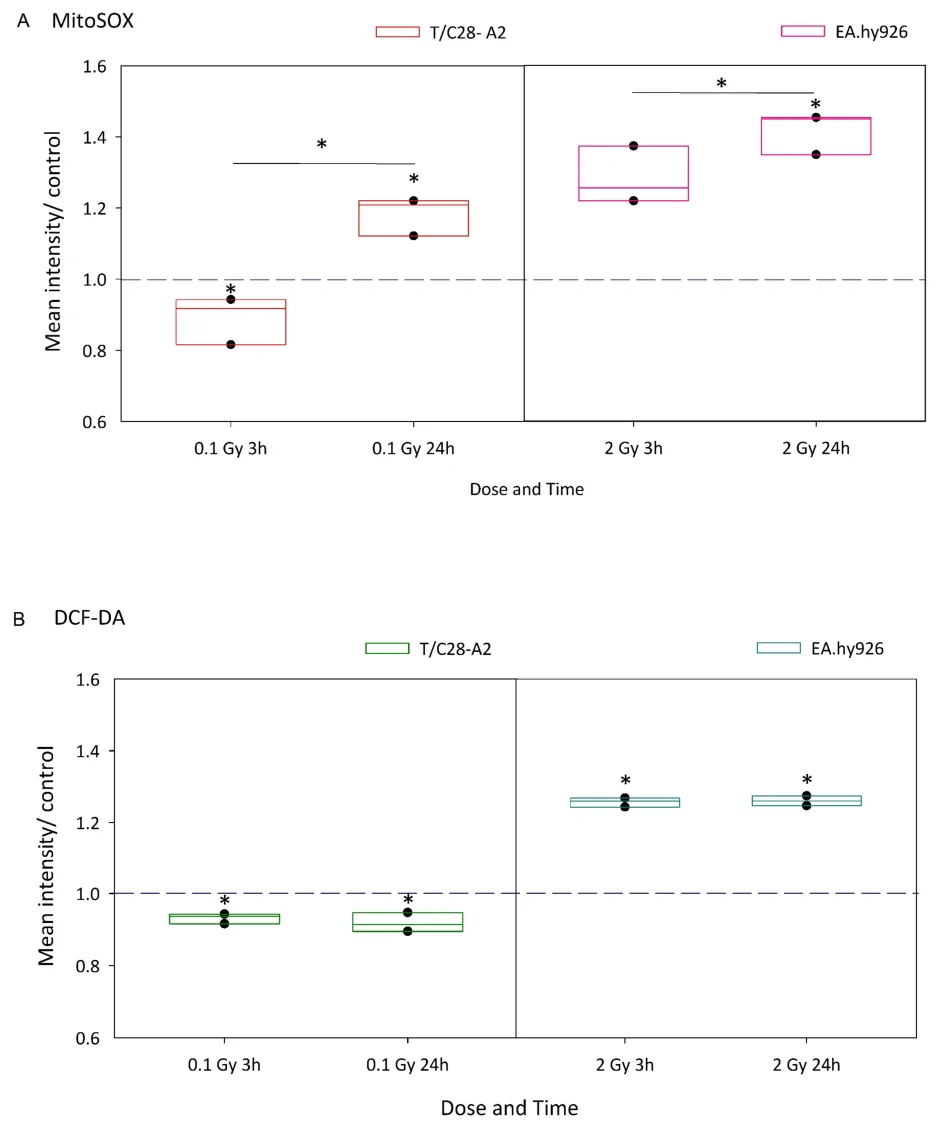
Oxidative stress levels in chondrocytes and endothelial cells following CM transfer from proton-irradiated chondrosarcoma cells with 0.1 Gy and 2 Gy, respectively. ROS production was assessed using MitoSOX (**A**) and DCF-DA (**B**) at 3 and 24 h post-CM transfer in T/C-28A2 (**left panels**) and EA.hy926 (**right panels**) cells. Data represent mean ± SEM (*n* = 3); statistical significance was calculated using the One-Way ANOVA method, followed by Fisher’s LSD post hoc test at * *p* < 0.05.

The results are illustrated in Figure 4. In T/C-28A2 chondrocytes a significant reduction in ROS levels was observed at 3 h post-exposure: mitochondrial superoxide levels decreased by approximately 10% (*p* = 0.015), while hydrogen peroxide levels dropped by about 18% (*p* < 0.001). Despite this initial decline, a more complex pattern emerged at 24 h, with a partial recovery of mitochondrial ROS and a continued reduction in total ROS levels, indicating a dynamic modulation of oxidative stress over time. Moreover, a significant temporal variation in mitochondrial ROS levels was detected between the two time points, suggesting activation of time-dependent redox regulatory mechanisms.

In EA.hy926 endothelial cells, a specific response was observed, with a significant increase in mitochondrial superoxide levels at 24 h (*p* = 0.0014). Additionally, total ROS levels were elevated at 3 h post-transfer and remained consistently high at 24 h, indicating sustained oxidative stress.

### 3.5. Proteomic Profiling

#### 3.5.1. Quantitative and Qualitative Analyses

We analyzed quantitative protein expression changes in bystander cells exposed to CM from proton-irradiated chondrosarcoma cell line SW1353, compared to sham controls.

**Figure 5:**
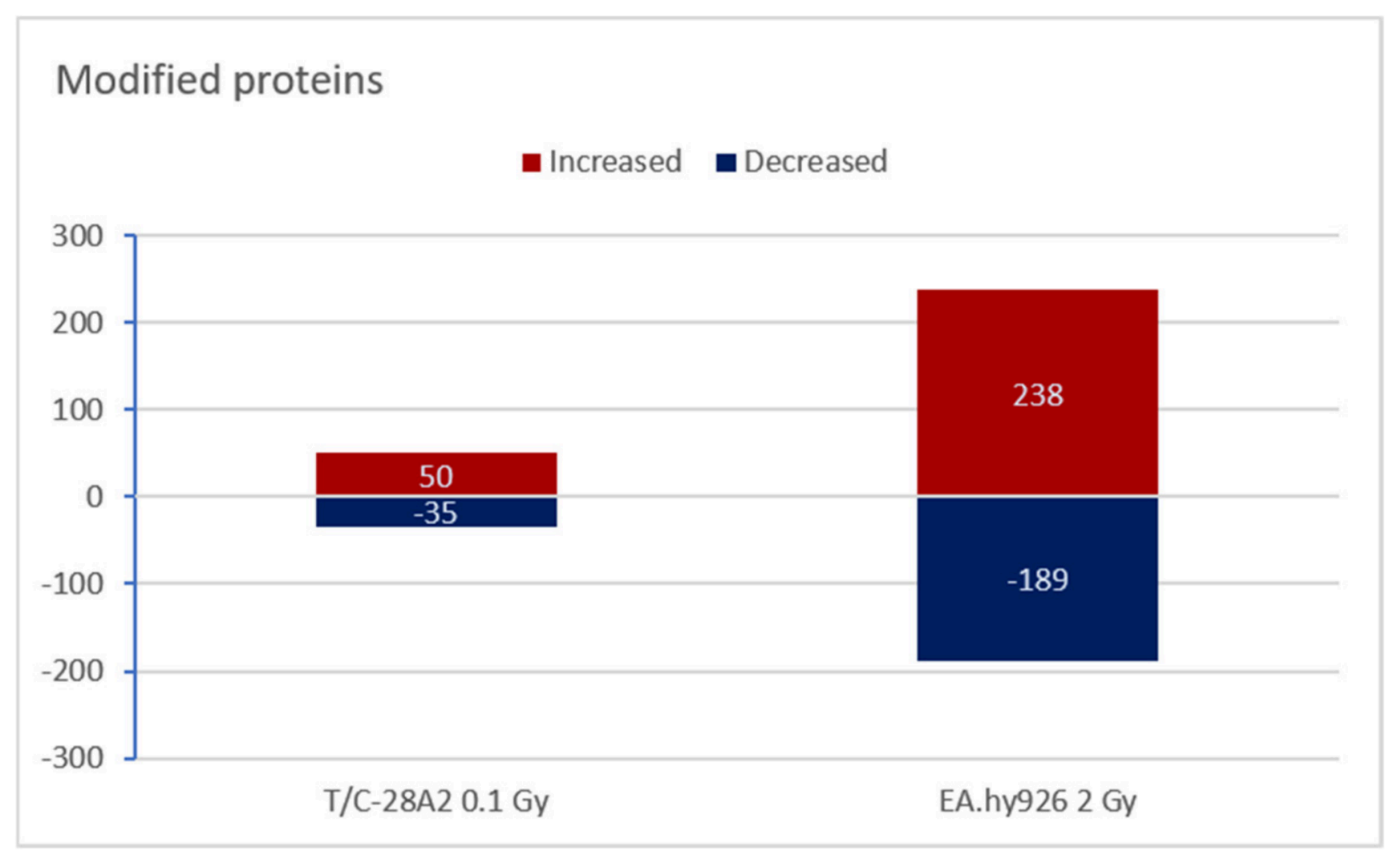
Protein expression changes induced by proton irradiation. Bar graphs represent the number of proteins showing increased and decreased abundance 24 h after transfer of CM from SW1353 cells irradiated with the indicated proton doses. The exact number of upregulated and downregulated proteins is displayed in each bar.

In T/C-28A2 chondrocytes exposed to medium from SW1353 cells irradiated with 0.1 Gy protons, 85 proteins showed differential expression (Figure 5), with 35 downregulated and 50 upregulated proteins compared to control (Supplementary Table S1). Meanwhile, in EA.hy926 endothelial cells treated with CM from SW1353 cells irradiated with 2 Gy protons, 427 proteins exhibited differential expression (Figure 5), with 189 downregulated and 238 upregulated proteins compared to control (Supplementary Table S2).

##### Highlights on the Differentially Abundant Proteins Following Irradiation

The bystander cells exposed to CM from proton-irradiated SW1353 chondrosarcoma cells revealed distinct modulation patterns in the chondrocytes and endothelial cells, indicating cell-type-specific responses to proton-induced bystander signaling.

In the T/C-28A2 bystander cells exposed to conditioned medium from low dose, a predominance of downregulated proteins was observed in the highest fold-changed accessions, with five out of the six most modulated proteins showing decreased expression (Table 1). This suggests an inhibition of cellular response possibly linked to proliferation (Q93045, −8.63 log_2_ fold, *p* < 0.001), extracellular matrix remodeling (Q92839, −7.03 log_2_ fold, *p* < 0.001), or cytoskeletal reorganization (Q9BZW7, −4.05 log_2_ fold, *p* < 0.001). These expression shifts may reflect mechanisms involved in tissue repair (P26010, −3.44 log_2_ fold, *p* = 0.022) or adaptive stress responses (Q92928, −4.39 log_2_ fold, *p* = 0.027). The low presence of upregulation observed may reflect selective activation of pathways that enhance survival or adaptive responses with the possibly to increase energy production under stress (A0A0D9SFB1, 2.31 log_2_ fold, *p* < 0.001).

In contrast, bystander EA.hy926 cells exposed to conditioned medium from a higher dose (2 Gy) showed the opposite trend, with the majority of modulated proteins (six out of nine) being upregulated (Table 1). This profile may reflect a tendency to promote bystander effect, potentially affecting energy metabolism (Q9BYZ2, 5.48 log_2_ fold, *p* = 0.0022), intracellular trafficking (O95716.4, 3.81 log_2_ fold, *p* = 0.0012), and mitochondrial RNA processing (Q96CM3, 3.55 log_2_ fold, *p* < 0.001). The upregulation of proteins involved in signal transduction (P27348.2, 4.72 log_2_ fold, *p* = 0.014) and RNA binding (P61129, 3.74 log_2_ fold, *p* = 0.009) further supports the presence of a paracrine-induced stress response. This protective or adaptive response may trigger cell cycle arrest (P21108, 3.76 log_2_ fold, *p* < 0.001) or reflect underlying mitochondrial dysfunction, possibly mediated through p53, AMPK, or TGF-β signaling pathways, which are associated with cellular stress responses and observed functional consequences of the affected proteins.

These results highlight a dose-dependent and cell-type-specific modulation of protein expression in bystander cells. While low-dose proton exposure appears to trigger degenerative responses in chondrocytes, higher doses lead to a predominantly permissive proteomic profile in endothelial-like cells, which may preserve their cellular functions.

##### Common and Specific Modulated Accessions Between Cell Types and Irradiation Doses

**Figure 6:**
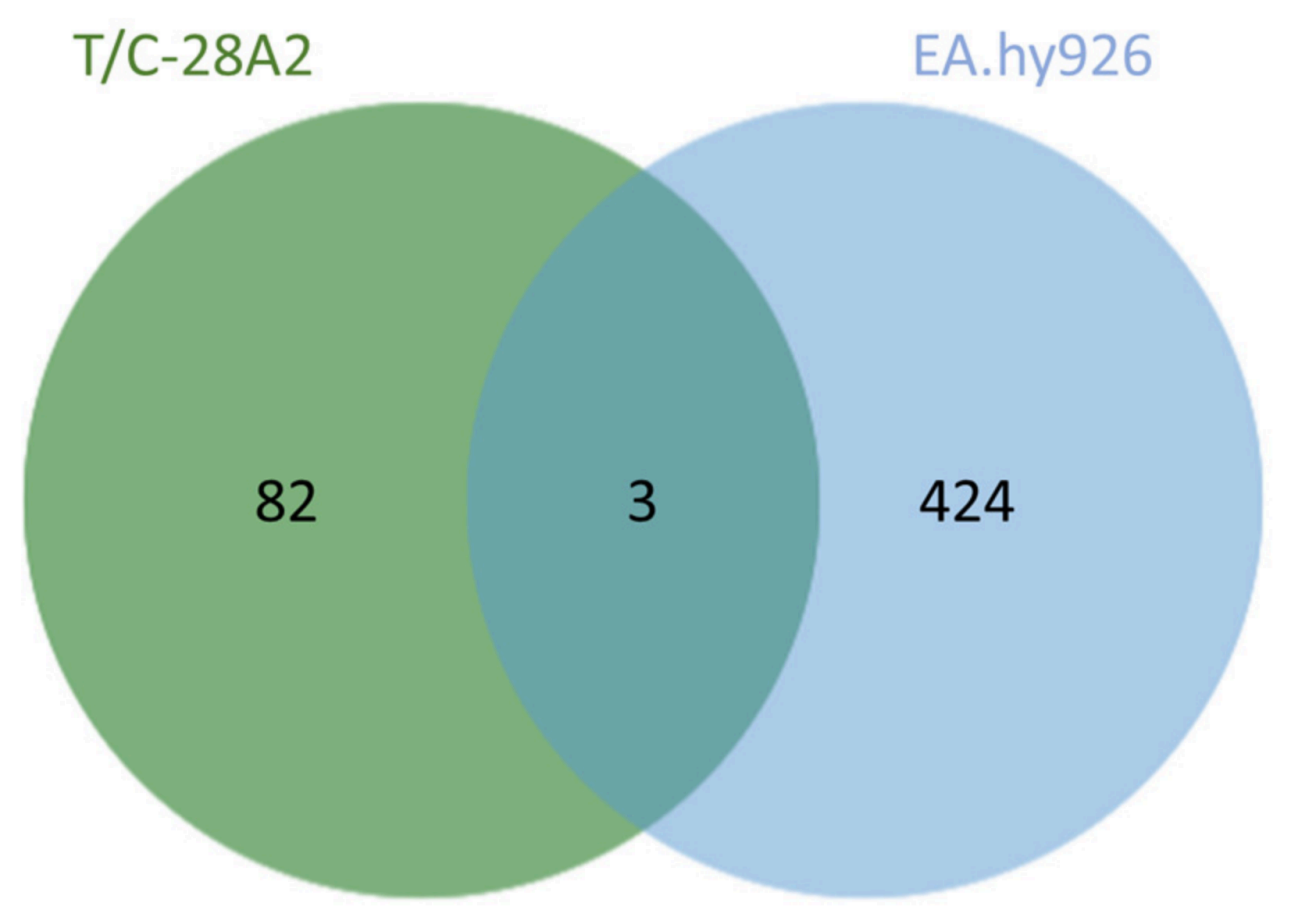
Venn diagram showing the distribution of differentially abundant proteins identified in the two analyzed cell lines. A total of 82 proteins were uniquely detected in the T/C-28A2 chondrocyte-like cell line, while 424 proteins were exclusive to the EA.hy926 endothelial cell line. Only three differentially abundant proteins were shared between the two cell lines: Q86YT6, P19105, and Q92839.

To identify proteins characteristic to each cell type and to investigate potential overlaps, a comparative proteomic analysis was performed on the differentially abundant proteins identified in the T/C-28A2 and EA.hy926 cell lines. The results are illustrated in a Venn diagram (Figure 6), which summarizes the number of unique and common proteins displaying significantly different abundance in both cell lines.

Out of the total differentially abundant, 82 were found exclusively in the T/C-28A2 cell line, suggesting these proteins may be involved in pathways specific to chondrocytic function or phenotype. In contrast, the EA.hy926 endothelial cell line exhibited a much broader unique proteome, with 424 differentially abundant proteins identified solely in this line, reflecting the complex molecular machinery associated with endothelial biology.

Interestingly, only three proteins were common between the two cell lines: E3 ubiquitin-protein ligase (Q86YT6), a regulator involved in Notch signaling and protein degradation; Myosin regulatory light chain 12A (P19105), which plays a role in cytoskeletal dynamics and contractility; and Hyaluronan synthase 1 (Q92839), an enzyme critical for the biosynthesis of hyaluronic acid, a key component of the extracellular matrix.

The minimal overlap reflects the functional and phenotypic divergence of these two cell lines, which guided our selection of distinct optimal donor doses. However, because the recipient cells received conditioned medium from donors irradiated at different doses (0.1 and 2 Gy), the observed proteomic profiles represent a combination of cell-type-specific responses and donor dose levels, rather than purely cell-type differences alone. The presence of hyaluronan synthase 1 in both lines could indicate a shared role in matrix production or cell–cell interactions, though potentially via different regulatory pathways.

Analysis of the protein dataset implicated exclusively in T/C-28A2 cells revealed the involvement of several inflammation-related signaling pathways—IL-17, PI3K-AKT, and TNF—relevant to chondrocyte activation under bystander inflammatory stimuli. Within the IL-17 signaling context, reduced levels of P31152, Q15628, and P21580 suggest that chondrocytes exposed to inflammatory mediators may exhibit reduced activity of atypical mitogen-activated protein kinases, which normally function in the upstream regulation of AKT by phosphorylating it at non-canonical sites and driving transcriptional reprogramming, likely contributing to the dampening of pro-inflammatory gene expression even in cells not directly exposed to cytokines.

Proteins implicated in the PI3K-AKT signaling pathway, such as P26010, O00459.1, and P01111.1, indicate that chondrocytes may suppress survival and metabolic pathways in response to integrin engagement and upstream Ras signaling. This suggests that bystander cells could inhibit protective pathways in response to paracrine inflammatory signals.

Furthermore, elements linked to TNF signaling, including Q15628, P21580, O00459.1, and A0A0D9SFB1, point to a potential involvement of mitochondrial dynamics and redox balance in chondrocyte stress responses. The increased abundance of A0A0D9SFB1, in particular, implies that TNF-related mitochondrial fragmentation and ROS generation may modulate inflammation-driven cartilage degradation, even in non-directly stimulated cells.

In EA.hy926 endothelial cells treated with medium derived from 2 Gy proton-irradiated SW1353 chondrosarcoma, we observed a decline in several signaling pathways related to stress response and intercellular communication. Notably, calcium signaling-related proteins showed a mixed modulation profile, with decreased expression observed for A0A2R8Y5A6, P62195.1, and Q9BSB4, alongside increased expression of P20226.1, P31749.3, P45983.1, Q14571.1, and Q96E52. This suggests that calcium homeostasis and mitochondrial crosstalk may be central to endothelial dysfunction under bystander inflammatory stress.

IL-17 pathway involvement was reflected by the presence of P25054, Q07955.1, P04049.1, and Q8TDR0, all of which are known to influence transcriptional regulation and inflammatory amplification in non-immune cells. Their expression indicates that IL-17-mediated responses may not be restricted to immune activation but also participate in vascular remodeling following bystander exposure.

Modulation of the PI3K-AKT signaling pathway was suggested by multiple proteins involved in integrin signaling, Ras-mediated pathways, and kinase cascades. Specifically, decreased expression was observed for P01111, P01112, P15153, P52333, and Q16512, whereas O15230, P07996, P27348 isoforms (P27348.2, P27348.3, P31946.3), P56199, Q00987, and Q15831 showed increased expression. This dual response in the PI3K-AKT cascade—which is classically known to regulate endothelial survival, permeability, and angiogenesis—suggests a complex remodeling of these cellular functions through paracrine signaling from stressed cells.

Furthermore, several proteins modulated under these conditions are involved in DNA repair, particularly in the regulation of nucleotide-excision repair pathways, including A0A0U1RQQ2, Q6STE5.3, F8VXC8, and P51531.2. These findings indicate a complex alteration of DNA maintenance mechanisms and a potential compensatory response to genotoxic stress in endothelial cells.

Finally, proteins associated with oxidative stress and mitochondrial function were predominantly upregulated, including P00395, P00414, P20674, P24539, Q9UDW1, O14957, Q99643, and O95258, with only O43676 showing decreased expression. Their modulation suggests mitochondrial stress and a heightened oxidative stress response in endothelial cells exposed to the inflammatory secretome of irradiated chondrosarcoma cells.

Together, these findings suggest that bystander chondrocytes and endothelial cells respond to inflammatory environments through the modulation of key signaling pathways that regulate inflammatory gene expression, cellular homeostasis, calcium dynamics, survival mechanisms, and mitochondrial stress responses.

#### 3.5.2. Gene Ontology Analysis

Bystander effects induced by proton irradiation affected distinct biological pathways in chondrocytes and endothelial cells depending on the radiation dose.

**Figure 7:**
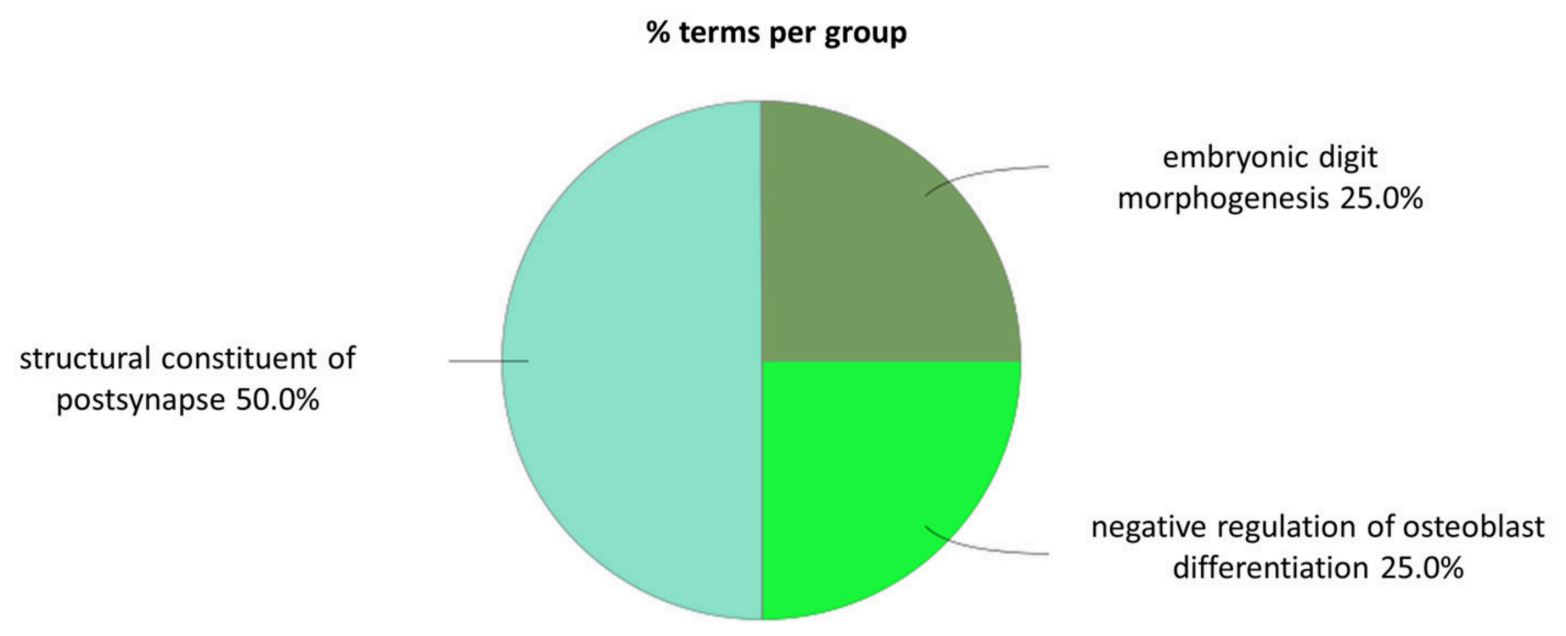
Representation of the proportions of different metabolic pathways in T/C-28A2 following medium transfer from SW1353 cells irradiated with protons, based on GO term enrichment for biological processes.

Gene Ontology (GO) enrichment in chondrocytes exposed to 0.1 Gy (Figure 7) revealed disruptions in developmental and differentiation processes, including embryonic digit morphogenesis, negative regulation of osteoblast differentiation, and cartilage development, indicating a potential impact on chondrogenesis and endochondral ossification. Moreover, alterations in proteins involved in synapse and postsynaptic structural components suggest potential impairments in cellular communication and cartilage microenvironment remodeling.

**Figure 8:**
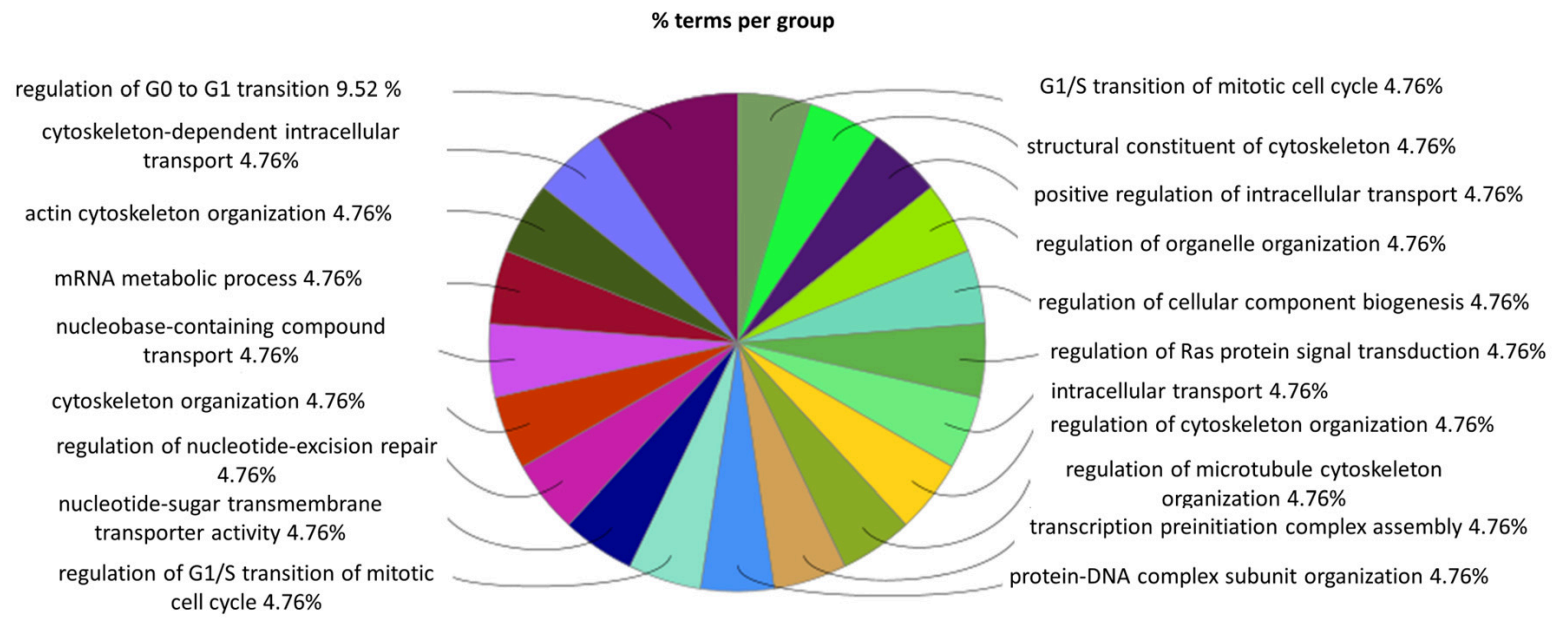
Representation of the proportions of different metabolic pathways in EA.hy926 following medium transfer from SW1353 cells irradiated with protons, based on GO term enrichment for biological processes.

In contrast, endothelial cells (Figure 8 and Supplementary Table S4) exposed to medium from SW1353 irradiated with 2 Gy protons exhibited enrichment in pathways related to G1/S transition of the mitotic cell cycle, cytoskeletal organization, nucleotide–sugar transmembrane transporter activity, and nucleotide-excision repair. These results highlight radiation-induced disruptions in cell proliferation, DNA repair, and cellular architecture, including actin-dependent processes crucial for cell integrity and intracellular transport. Additional pathways such as mRNA metabolism, transport of nitrogenous compounds, and organelle organization further underscore the broader impact of proton irradiation on cellular homeostasis and gene expression.

#### 3.5.3. Network Analysis of Modulated Accessions

STRING network analysis (Supplementary Figure S1) of proteomic alterations in T/C-28A2 chondrocytes and EA.hy926 endothelial cells, following exposure to CM from proton-irradiated SW1353 cells, revealed distinct patterns reflecting the differential impact of radiation dose on cellular responses.

To complement pathway-level findings in bystander T/C-28A2 cells (Supplementary Figure S1A), we constructed a protein–protein interaction network using the STRING database (confidence threshold: 0.4). Although no statistically significant pathway enrichment was identified under these parameters, the network topology highlighted several central proteins with potential regulatory influence in the context of inflammatory stress.

A0A2R8YEA7, an isoform of P60709, emerged as a prominent hub, displaying multiple interactions with transcriptional regulators and structural proteins, including P37275, Q15672, and P52926. These associations point toward a cytoskeletal–nuclear axis potentially involved in transcriptional adaptation during bystander signaling. Interestingly, proteins already implicated in signaling pathways in chondrocytes, such as P31152 in IL-17 signaling, and Q15628 and P21580 in both IL-17 and TNF pathways, also appeared connected, supporting their involvement beyond isolated pathway nodes.

Additional links between P26010 and A0A0D9SFB1 suggest convergence between membrane trafficking, integrin signaling, and inflammatory modulation—mechanisms that may fine-tune the chondrocyte response under non-irradiated, bystander conditions.

While the network as a whole does not highlight specific canonical pathways, the spatial organization of nodes and the presence of known signaling mediators such as Q9H9G7, P52657, and P19105 indicate the activation of diverse cellular processes, including post-transcriptional gene regulation, transcriptional initiation, and contractility.

Conversely, EA.hy926 endothelial cells (Supplementary Figure S1B and Supplementary Table S5) exposed to CM from SW1353 cells irradiated with 2 Gy revealed a strong enrichment for general cellular and metabolic processes (GO:0044237; GO:0008152), as well as components related to intracellular compartments and organelle structure, including the cytoplasm (GO:0005737), nucleus (GO:0005634), and multiple membrane-bounded organelles (GO:0043227, GO:0043229).

Notably, mitochondrial components were highly represented, with terms such as mitochondrial inner membrane (GO:0005743), respirasome (GO:0005746), and respiratory chain complex (GO:0098803) suggesting that mitochondrial homeostasis and energy metabolism are key features of the bystander response. Enrichment in electron transport chain-associated pathways (hsa00190, WP111) and oxidative phosphorylation also points toward altered mitochondrial dynamics in endothelial cells, alongside involvement of chromatin-remodeling complexes such as npBAF and bBAF (GO:71564, GO:0140092).

In parallel, structural and trafficking components such as the endoplasmic reticulum membrane (GO:0005789), the myosin complex (GO:0016459), and calmodulin-binding proteins (KW-0112) were annotated, indicating potential alterations in intracellular transport and cytoskeletal organization, as well as pathways linked to macroautophagy and selective autophagy (HSA-1632852, HSA-9663891). These findings complement the pathway-specific results by highlighting organelle-level disruptions and energy stress responses that may underpin the broader phenotypic alterations observed in bystander endothelial cells.

Also, based on the interaction strength and a comprehensive network analysis, complex interactions among proteins involved in multiple critical pathways were revealed, including amyotrophic lateral sclerosis (ALS), thermogenesis, oxidative phosphorylation, central carbon metabolism in cancer, general cellular metabolic processes, neurodegenerative disorders, chromatin remodeling, and autophagy. The ALS pathway underscores radiation’s influence on stress responses and potential neurodegenerative processes, while thermogenesis reflects alterations in energy expenditure and metabolic heat production. Oxidative phosphorylation, involving key proteins such as Q9UDW1, O14957, and P20674, indicates substantial changes in mitochondrial function and ATP production, which are essential for cellular energy metabolism and adaptation. The central carbon metabolism pathway highlights shifts in metabolic processes critical for cancer cell survival and proliferation, whereas general metabolic process changes reflect broader alterations in cellular metabolic activity, further reinforced by transcriptional regulation via BAF complexes and cellular quality control mechanisms.

## 4. Discussion

The investigation of bystander effects mediated by CM from proton-irradiated SW1353 chondrosarcoma cells revealed distinct cellular responses between chondrocytes and endothelial cells.

One of the most notable findings of the present study is the contrasting biological behavior of the two recipient cell types; chondrocytes responded only to CM generated after low-dose irradiation (0.1 Gy), whereas endothelial cells were considerably more susceptible to bystander signals released following therapeutic-dose irradiation (2 Gy). However, both cell populations exhibited evidence of non-linear, dose-dependent bystander responses expressed by reduced clonogenic survival as well as increased MN formation. These observations align with previous studies reporting increased MN formation at low radiation doses (0.05–0.1 Gy for carbon ions; 0.1–0.2 Gy for X-rays) in similar bystander models [5].

The observed differences between T/C-28A2 and EA.hy926 cells suggest that the response to conditioned medium from proton-irradiated chondrosarcoma cells depends on the recipient model and the irradiation dose delivered to the donor cells. Chondrocytes exhibited more pronounced functional effects following exposure to CM obtained from cells irradiated at 0.1 Gy, whereas endothelial cells showed stronger responses following exposure to CM obtained from cells irradiated at 2 Gy. These findings indicate that the relationship between donor irradiation dose and bystander response may differ between recipient cell types.

Nevertheless, an important limitation of the experimental design is that the subsequent cell cycle, ROS, and proteomic analyses were performed under selected conditions based on the strongest responses observed in the initial functional assays. Consequently, the molecular profiles obtained for the two recipient cell types correspond to different donor irradiation doses. The observed differences therefore cannot be attributed exclusively to recipient cell identity, because cell type and donor irradiation dose are not independently controlled in these selected comparisons. A systematic comparison of both recipient cell types across all irradiation doses would be required to distinguish more clearly between cell-type-dependent and dose-dependent effects.

Additionally, such cell-type-specific responses reinforce previous reports indicating that bystander effects varies significantly based on cell line and irradiation type [4,12,32]. Ultimately, these differential sensitivities underscore the necessity of considering lineage-specific risks in proton therapy [4,5].

This divergence is further reflected in cell cycle dynamics, oxidative stress and proteomic remodeling. While chondrocytes maintained a stable cell cycle, endothelial cells exhibited significant G1- and G2-phase depletion, suggesting a more profound disruption of progression. This aligns with identified G1/S and mitotic G2 DNA damage checkpoints as specific bystander responses to low-dose ion irradiation [6].

Mechanistically, chondrocytes exhibited an early reduction in mitochondrial ROS, indicating an adaptive antioxidant response similar to redox signatures observed following low-dose X-ray exposure [6,21]. Conversely, endothelial cells mirrored direct radiation effects [33], showing sustained increases in ROS after exposure to 2 Gy derived medium, suggesting a transition to chronic oxidative stress.

While functional assays capture the phenotypic outcomes of bystander signaling, proteomic profiling serves as an exploratory, hypothesis-generating tool to map underlying molecular alterations. This approach identifies valuable preliminary candidate pathways, establishing a framework for future mechanistic studies on normal cell responses within radioresistant tumor microenvironments. In our dataset, the proteomic profile of bystander chondrocytes at 0.1 Gy suggests an adaptive-like impairment [6]. The downregulation of Q93045 and Q92839 indicates a shift toward cell cycle arrest and ECM remodeling—processes linked to radiation sensitivity and inflammatory signaling [34,35,36,37,38,39,40,41,42]. Furthermore, Q9BZW7 and P26010 suggest coordinated cytoskeletal and integrin reorganization. While Q9BZW7 restricts migration and angiogenesis [43,44,45,46], P26010 modulation contrasts with patterns seen in radioresistant cancers where it mediates TRIM2 oncogenic function [47,48,49,50,51,52,53]. This remodeling, likely driven by the vesicular regulator Q92928 [54,55,56,57,58,59], may be triggered by collagen-related peptides cleaved during irradiation [60]. Conversely, endothelial cells exposed to 2 Gy conditioned bystander medium exhibited a stimulating profile. The upregulation of Q9BYZ2 indicates enhanced pyruvate metabolism, a protein linked to radioresistance [61,62,63,64]. This is compounded by the activation of Q96CM3, essential for mitochondrial respiratory activity and ribosomal biogenesis [65,66,67,68]. Signal transduction and vesicle transport were also targeted; the upregulation of O95716.4 may promote secretory functions required for motility and EMT [69,70,71,72,73,74,75,76,77,78]. Furthermore, the activation of P27348.2 and P61129 mitigates this paracrine stress, as these proteins are typically involved in DNA repair, stress response, and cell viability [54,79,80,81,82,83,84]. Specifically, P27348.2 shows reduced abundance following hydroquinidine treatment in breast and ovarian cancers, is implicated in DNA damage repair process through PARP1 regulation and can serve as a sensitive target in chemotherapy, described as hypo-variable, and is associated with regulatory processes and stress response in childhood cancer survivors in response to radiation doses [85,86]. P61129 is associated with regulatory processes and stress response, appearing downregulated in rectal cancer patients or following exposure to nanoliposomal ASOmiR-21/373 [87,88,89,90].

GO enrichment substantiated these distinctions: chondrocytes displayed altered developmental and inflammatory pathways (IL-17, PI3K-AKT, TNF), while endothelial cells showed broader impairments in DNA repair, cytoskeletal organization, and oxidative phosphorylation. While direct radiation activates p53 and senescence, bystander cells mirror this through G1/S transition and nucleotide-excision repair [91,92].

A comparative analysis identified minimal overlap, with only Q86YT6, P19105, and Q92839 shared. The presence of Q92839 in both cell types suggests a shared functional role in extracellular matrix dynamics and inflammatory signaling, although likely operating through distinct signaling contexts within each lineage [38,39,40,41,42]. Q86YT6 implies involvement in NOTCH activation [93,94], while P19105 acts as a node for carcinogenesis and lethal X-ray responses, linked to survival and metastasis [95,96,97,98,99,100,101].

Network analysis identified A0A2R8YEA7 as a hub in chondrocytes, connecting cytoskeletal and nuclear axes for transcriptional reprogramming. Its upregulation relates to dose-specific responses and positive regulation of DNA damage repair [102,103,104,105]. Signaling mediators like P31152 and Q15628 further contribute to mitochondrial regulation; P31152 is linked to radiation resistance and the modulation of osteoarthritis [106,107]. Complementing this, Q15628 facilitates TNF-α-induced cell death and superoxide production through complexes with Nox1, while also participating in caspase-mediated apoptotic signaling, RIPK1 kinase activity, and mitochondrial adaptor interactions [108,109,110,111,112]. These molecular interactions potentially exacerbate cartilage degradation through the secretion of pro-inflammatory cytokines into the microenvironment. Although these signaling molecules are key players in bystander effects related to apoptosis [113,114], the observed increase in cytokine secretion at a dose of 2 Gy [7] suggests a complex, dose-dependent modulation of the inflammatory environment that governs the final bystander response.

The proteomic analysis provides an additional molecular perspective on the functional responses observed in the two recipient cell models. The identification of 85 differentially abundant proteins in T/C-28A2 cells and 427 in EA.hy926 cells, with only three proteins shared between the two profiles, indicates that the selected experimental conditions were associated with substantially different patterns of protein abundance changes. These differences provide a basis for generating hypotheses about the molecular processes associated with the responses of each recipient model.

However, differential protein abundance and functional enrichment do not, by themselves, demonstrate that the identified proteins directly cause the observed changes in clonogenic survival, micronucleus formation, cell cycle distribution, or ROS levels. Similarly, protein–protein interaction networks identify potential functional relationships but do not establish direct interactions or causal regulatory mechanisms under the experimental conditions used. The observed enrichment of pathways associated with proliferation, extracellular matrix organization, metabolism, and DNA repair should therefore be interpreted as indicative of molecular processes potentially involved in the response to bystander signaling rather than as definitive evidence of their functional activation or suppression.

In particular, the protein-level changes observed in chondrocytes may be consistent with alterations in processes related to proliferation and extracellular matrix organization. However, the present data do not establish a pro-regenerative phenotype or demonstrate enhanced tissue repair capacity. Likewise, the extensive proteomic changes observed in endothelial cells, together with the functional and oxidative stress measurements, suggest alterations in cellular processes associated with metabolic activity and the maintenance of genomic stability. Nevertheless, the proteomic data alone do not establish a generalized loss of metabolic or genomic integrity. These interpretations require further validation through targeted protein measurements and functional assays specifically designed to assess the proposed processes.

Several limitations should be considered when interpreting these findings. First, the study used established cell lines rather than primary chondrocytes and endothelial cells. T/C-28A2 is an immortalized chondrocyte cell line, while EA.hy926 is a hybrid cell line generated by fusion of human umbilical vein endothelial cells with the A549 lung carcinoma cell line. Although these models are useful for investigating cellular responses under controlled experimental conditions, they may not fully reproduce the biological characteristics of primary healthy cartilage and vascular tissues. Therefore, the observed effects should be interpreted as responses of the selected in vitro models rather than direct evidence of tissue-level effects in vivo.

Second, the more detailed molecular analyses were conducted under selected conditions corresponding to the strongest functional responses in each recipient model. Because these conditions involved different donor irradiation doses, the relative contributions of recipient cell identity and donor dose cannot be fully separated. Future studies should investigate both recipient cell types across the complete dose range, ideally using primary cells and additional biological replicates, to determine whether the observed differences are reproducible and attributable to cell-specific responses.

Finally, the proteomic findings and network-based analyses identify candidate proteins and pathways associated with the observed responses, but they do not establish causality or validate the proposed molecular mechanisms. Targeted protein validation and functional perturbation experiments will be necessary to determine whether the identified candidates contribute directly to the observed phenotypes. Accordingly, the potential relevance of these proteins as biomarkers of radiation-induced bystander responses remains exploratory and requires independent validation.

However, the integration of functional assays with quantitative proteomics represents a major strength of this study, allowing phenotypic changes to be linked with underlying molecular pathways.

## 5. Conclusions

This study demonstrates that conditioned medium from proton-irradiated SW1353 chondrosarcoma cells can induce distinct functional and proteomic responses in T/C-28A2 chondrocytes and EA.hy926 endothelial cells. Under the conditions investigated, chondrocytes showed more pronounced responses to conditioned medium obtained following 0.1 Gy irradiation, whereas endothelial cells exhibited stronger responses to conditioned medium obtained following 2 Gy irradiation. These differences were accompanied by distinct protein abundance profiles, suggesting that the responses of the two recipient models involve different molecular processes.

The proteomic analysis identified candidate proteins and biological pathways potentially associated with the observed responses, providing a basis for further investigation of the molecular processes involved in proton-induced bystander signaling. However, the results do not establish a pro-regenerative effect in chondrocytes, a generalized loss of metabolic or genomic integrity in endothelial cells, or the validity of the identified proteins as biomarkers. Furthermore, because the detailed molecular analyses were performed at different donor irradiation doses for the two recipient cell types, the contribution of recipient cell identity cannot be fully separated from that of donor irradiation dose.

Overall, these findings highlight the importance of considering both donor irradiation dose and recipient cell characteristics when investigating radiation-induced bystander effects. Further studies using primary cells, systematic dose comparisons, and targeted functional validation are needed to establish the biological significance and potential translational relevance of the identified molecular changes.

## Figures and Tables

**Table 1:** List of the most modulated proteins in the proteome of bystander cells after receiving CM from SW1353 chondrosarcoma cells irradiated with protons (selected from Supplementary Tables S1 and S2).

Entry T/C-28A2 0.1 Gy	Protein Name	Log_2_ Fold	Entry EA.hy926 2 Gy	Protein Name	Log_2_ Fold
A0A0D9SFB1	dynamin 1	2.31	Q9BYZ2	lactate dehydrogenase A like 6B	5.48
P26010	integrin subunit beta 7	−3.44	P27348.2	tyrosine 3-monooxygenase/tryptophan 5-monooxygenase activation protein theta	4.72
Q9BZW7	testis specific 10	−4.05	O95716.4	RAB3D, member RAS oncogene family	3.81
Q92928	RAB1C, member RAS oncogene family (pseudogene)	−4.39	P21108	phosphoribosyl pyrophosphate synthetase 1 like 1	3.76
Q92839	hyaluronan synthase 1	−7.03	P61129	zinc finger CCCH-type containing 6	3.74
Q93045	stathmin 2	−8.63	Q96CM3	RNA pseudouridine synthase D4	3.55
			P00338.3	lactate dehydrogenase A	−3.05
			O00462.1	mannosidase beta	−3.26
			Q8TF01	PNN interacting serine and arginine rich protein	−3.31

## Data Availability

All proteomic data have been deposited in the iProX public repository database, with the code IPX0019367001 (https://www.iprox.cn, accessed on 22 October 2026).

## Supplementary Materials

The following supporting information can be downloaded at https://www.mdpi.com/article/10.3390/cancers18213534/s1, Table S1: Proteins differentially abundant in T/C-28A2 chondrocytes exposed to conditioned medium (CM) from SW1353 cells irradiated with 0.1 Gy protons, compared with sham-CM; Table S2: Proteins differentially abundant in EA.hy926 endothelial cells exposed to conditioned medium (CM) from SW1353 cells irradiated with 2 Gy protons, compared with sham-CM; Table S3: Gene Ontology (GO) term enrichment for "bystander" chondrocytes T/C28-A2, which received medium from SW1353 chondrosarcoma cells irradiated with protons at a dose of 0.1 Gy; Table S4: Gene Ontology (GO) term enrichment for "bystander" endothelial cells EA.hy926, which received medium from SW1353 chondrosarcoma cells irradiated with protons at a dose of 2 Gy; Table S5: STRING Network enrichment and associated proteins for EA.hy926 after receiving medium from SW1353 exposed to 2 Gy; Figure S1: Identity and interaction of proteins within functional network that are altered 24 h after conditioned medium transfer in chondrocyte and endothelial cells.

## Abbreviations

The following abbreviations are used in this manuscript:

BSA	Bovine serum albumin
CHS	Chondrosarcoma
CM	Conditioned medium
DDA	Data-dependent acquisition
DIA	Data-independent acquisition
ECM	Extracellular matrix
EDTA	Ethylenediaminetetraacetic acid
EMT	Epithelial-to-mesenchymal transition
FDR	False discovery rate
G1/G2	Gap 1 phase/Gap 2 phase (cell cycle)
GO	Gene Ontology
HBSS	Hank’s Balanced Salt Solution
IL-17	Interleukin 17
JAK/STAT	Janus kinase/signal transducer and activator of transcription
LC-MS/MS	Liquid chromatography–tandem mass spectrometry
LET	Linear energy transfer
LFQ	Label-free quantification
MN	Micronucleus
m/z	Mass-to-charge ratio
MN	Micronuclei
MS	Mass spectrometry
MTT	3-(4,5-dimethylthiazol-2-yl)-2,5-diphenyltetrazolium bromide
NaCl	Sodium chloride
NF-κB	Nuclear factor kappa-light-chain-enhancer of activated B cells
pCT	Proton computed tomography
PE	Plating efficiency
PI3K-AKT	Phosphoinositide 3-kinase-protein kinase B
PPI	Protein–protein interaction
RIBEs	Radiation-induced bystander effects
RIPA	Radioimmunoprecipitation assay (buffer)
ROS	Reactive oxygen species
RT	Retention time
SF	Survival fraction
TIMS-TOF	Trapped ion mobility spectrometry–time of flight
TNF	Tumor necrosis factor
XIC	Extracted ion chromatogram

## References

[1] Pouget J.P., Georgakilas A.G., Ravanat J.L. (2018). Targeted and Off-Target (Bystander and Abscopal) Effects of Radiation Therapy: Redox Mechanisms and Risk/Benefit Analysis. Antioxid. Redox Signal..

[2] Mothersill C., Seymour C.B. (1998). Cell-Cell Contact during Gamma Irradiation Is Not Required to Induce a Bystander Effect in Normal Human Keratinocytes: Evidence for Release during Irradiation of a Signal Controlling Survival into the Medium. Radiat. Res..

[3] Azzam E.I., De Toledo S.M., Little J.B. (2001). Direct Evidence for the Participation of Gap Junction-Mediated Intercellular Communication in the Transmission of Damage Signals from α-Particle Irradiated to Nonirradiated Cells. Proc. Natl. Acad. Sci. USA.

[4] Wakatsuki M., Magpayo N., Kawamura H., Held K.D. (2012). Differential Bystander Signaling between Radioresistant Chondrosarcoma Cells and Fibroblasts after X-Ray, Proton, Iron Ion and Carbon Ion Exposures. Int. J. Radiat. Oncol. Biol. Phys..

[5] Lepleux C., Marie-Brasset A., Temelie M., Boulanger M., Brotin É., Goldring M.B., Hirtz C., Varès G., Nakajima T., Saintigny Y. (2019). Bystander Effectors of Chondrosarcoma Cells Irradiated at Different LET Impair Proliferation of Chondrocytes. J. Cell Commun. Signal..

[6] Gilbert A., Payet V., Bernay B., Chartier-Garcia E., Testard I., Candéias S.M., Chevalier F. (2022). Label-Free Direct Mass Spectrometry Analysis of the Bystander Effects Induced in Chondrocytes by Chondrosarcoma Cells Irradiated with X-Rays and Carbon Ions. Front. Biosci..

[7] Stelcer E., Kulcenty K., Rucinski M., Kruszyna-Mochalska M., Skrobala A., Sobecka A., Jopek K., Suchorska W.M. (2021). Ionizing Radiation Exposure of Stem Cell-Derived Chondrocytes Affects Their Gene and MicroRNA Expression Profiles and Cytokine Production. Sci. Rep..

[8] Thariat J., Valable S., Laurent C., Haghdoost S., Pérès E.A., Bernaudin M., Sichel F., Lesueur P., Césaire M., Petit E. (2020). Hadrontherapy Interactions in Molecular and Cellular Biology. Int. J. Mol. Sci..

[9] Laurent C. (2018). Oxidative Stress in Hadrontherapy. Novel Prospects in Oxidative and Nitrosative Stress.

[10] Jafarian-Dehkordi F., Hoeschen C. (2025). Low-Dose Radiation Risk in Medicine: A Look at Risk Models, Challenges, and Future Prospects. Z. Med. Phys..

[11] Verma N., Tiku A.B. (2017). Significance and Nature of Bystander Responses Induced by Various Agents. Mutat. Res. Mutat. Res..

[12] Mukherjee S., Chakraborty A. (2018). Radiation-Induced Bystander Phenomenon: Insight and Implications in Radiotherapy. Int. J. Radiat. Biol..

[13] Xing J.L., Stea B. (2024). Molecular Mechanisms of Sensitivity and Resistance to Radiotherapy. Clin. Exp. Metastasis.

[14] Konkova M.S., Kaliyanov A.A., Sergeeva V.A., Abramova M.S., Kostyuk S.V. (2019). Oxidized Cell-Free DNA Is a Factor of Stress Signaling in Radiation-Induced Bystander Effects in Different Types of Human Cells. Int. J. Genom..

[15] Gao L., Zhang A. (2023). Low-Dose Radiotherapy Effects the Progression of Anti-Tumor Response. Transl. Oncol..

[16] Wang J., Zhang J., Wen W., Wang F., Wu M., Chen D. (2023). BBA—Molecular Basis of Disease Exploring Low-Dose Radiotherapy to Overcome Radio-Immunotherapy Resistance. BBA—Mol. Basis Dis..

[17] Hellweg C.E., AliChishti A., Diegeler S., Spitta L.F., Henschenmacher B., Baumstark-Khan C. (2018). Molecular Signaling in Response to Charged Particle Exposures and Its Importance in Particle Therapy. Int. J. Part. Ther..

[18] Obrador E., Estrela J.M., López-Blanch R., Moreno-Murciano P., Montoro A., Oriol-Caballo M. (2026). Radiomitigators: Breakthroughs in Post-Radiation Recovery. Antioxidants.

[19] Goldring M.B., Birkhead J.R., Suen L.F., Yamin R., Mizuno S., Glowacki J., Arbiser J.L., Apperley J.F. (1994). Interleukin-1 Beta-Modulated Gene Expression in Immortalized Human Chondrocytes. J. Clin. Investig..

[20] Finger F., Schörle C., Zien A., Gebhard P., Goldring M.B., Aigner T. (2003). Molecular Phenotyping of Human Chondrocyte Cell Lines T/C-28a2, T/C-28a4, and C-28/I2. Arthritis Rheum..

[21] Tudor M., Gilbert A., Lepleux C., Temelie M., Hem S., Armengaud J., Brotin E., Haghdoost S., Savu D., Chevalier F. (2021). A Proteomic Study Suggests Stress Granules as New Potential Actors in Radiation-induced Bystander Effects. Int. J. Mol. Sci..

[22] Edgell C.J.S., McDonald C.C., Graham J.B. (1983). Permanent Cell Line Expressing Human Factor VIII-Related Antigen Established by Hybridization. Proc. Natl. Acad. Sci. USA.

[23] Ahn K., Pan S., Beningo K., Hupe D. (1995). A Permanent Human Cell Line (EA.Hy926) Preserves the Characteristics of Endothelin Converting Enzyme from Primary Human Umbilical Vein Endothelial Cells. Life Sci..

[24] Tudor M., Popescu R.C., Bernay B., Temelie M., Craciun L., Esanu T.R., Chevalier F., Savu D.I. (2026). Proton Irradiation Induces Differential Cellular Responses and Proteomic Signatures in Chondrosarcoma and Chondrocytes. Curr. Issues Mol. Biol..

[25] Tudor M., Popescu R.C., Negoita R.D., Gilbert A., Ilisanu M.A., Temelie M., Dinischiotu A., Chevalier F., Mihailescu M., Savu D.I. (2023). In Vitro Hyperspectral Biomarkers of Human Chondrosarcoma Cells in Nanoparticle-Mediated Radiosensitization Using Carbon Ions. Sci. Rep..

[26] Tudor M., Popescu R.C., Irimescu I.N., Rzyanina A., Tarba N., Dinischiotu A., Craciun L., Esanu T.R., Vasile E., Hotnog A.T. (2024). Enhancing Proton Radiosensitivity of Chondrosarcoma Using Nanoparticle-Based Drug Delivery Approaches: A Comparative Study of High- and Low-Energy Protons. Int. J. Mol. Sci..

[27] Fenech M. (2007). Cytokinesis-Block Micronucleus Cytome Assay. Nat. Protoc..

[28] Shannon P., Markiel A., Ozier O., Baliga N.S., Wang J.T., Ramage D., Amin N., Schwikowski B.B., Ideker T. (2003). Cytoscape: A Software Environment for Integrated Models of Biomolecular Interaction Networks. Genome Res..

[29] Szklarczyk D., Gable A.L., Lyon D., Junge A., Wyder S., Huerta-Cepas J., Simonovic M., Doncheva N.T., Morris J.H., Bork P. (2019). STRING V11: Protein-Protein Association Networks with Increased Coverage, Supporting Functional Discovery in Genome-Wide Experimental Datasets. Nucleic Acids Res..

[30] Szklarczyk D., Kirsch R., Koutrouli M., Nastou K., Mehryary F., Hachilif R., Gable A.L., Fang T., Doncheva N.T., Pyysalo S. (2023). The STRING Database in 2023: Protein-Protein Association Networks and Functional Enrichment Analyses for Any Sequenced Genome of Interest. Nucleic Acids Res..

[31] Szklarczyk D., Nastou K., Koutrouli M., Kirsch R., Mehryary F., Hachilif R., Hu D., Peluso M.E., Huang Q., Fang T. (2025). The STRING Database in 2025: Protein Networks with Directionality of Regulation. Nucleic Acids Res..

[32] Luong N.C., Kawamura H., Ikeda H., Roppongi R.T., Shibata A., Hu J., Jiang J.G., Yu D.S., Held K.D. (2024). ATR Signaling Controls the Bystander Responses of Human Chondrosarcoma Cells by Promoting RAD51-Dependent DNA Repair. Int. J. Radiat. Biol..

[33] Large M., Reichert S., Hehlgans S., Fournier C., Rödel C., Rödel F. (2014). A Non-Linear Detection of Phospho-Histone H2AX in EA.Hy926 Endothelial Cells Following Low-Dose X-Irradiation Is Modulated by Reactive Oxygen Species. Radiat. Oncol..

[34] Zhong F.J., Sun B., Cao M.M., Xu C., Li Y.M., Yang L.Y. (2021). STMN2 Mediates Nuclear Translocation of Smad2/3 and Enhances TGFβ Signaling by Destabilizing Microtubules to Promote Epithelial-Mesenchymal Transition in Hepatocellular Carcinoma. Cancer Lett..

[35] Shao M., Wang L., Zhang Q., Wang T., Wang S. (2022). STMN2 Overexpression Promotes Cell Proliferation and EMT in Pancreatic Cancer Mediated by WNT/β-Catenin Signaling. Cancer Gene Ther..

[36] Rogers D.A., Schor N.F. (2013). Kidins220/ARMS Depletion Is Associated with the Neural-to Schwann-like Transition in a Human Neuroblastoma Cell Line Model. Exp. Cell Res..

[37] Prudencio M., Humphrey J., Pickles S., Brown A.L., Hill S.E., Kachergus J.M., Shi J., Heckman M.G., Spiegel M.R., Cook C. (2025). Truncated Stathmin-2 Is a Marker of TDP-43 Pathology in Frontotemporal Dementia. J. Clin. Investig..

[38] Elyada E., Bolisetty M., Laise P., Flynn W.F., Courtois E.T., Burkhart R.A., Teinor J.A., Belleau P., Biffi G., Lucito M.S. (2019). Cross-Species Single-Cell Analysis of Pancreatic Ductal Adenocarcinoma Reveals Antigen-Presenting Cancer-Associated Fibroblasts. Cancer Discov..

[39] Hasegawa K., Saga R., Takahashi R., Fukui R., Chiba M., Okumura K., Tsuruga E., Hosokawa Y. (2020). 4-Methylumbelliferone Inhibits Clonogenic Potency by Suppressing High Molecular Weight-Hyaluronan in Fibrosarcoma Cells. Oncol. Lett..

[40] Lierova A., Kasparova J., Filipova A., Cizkova J., Pekarova L., Korecka L., Mannova N., Bilkova Z., Sinkorova Z. (2022). Hyaluronic Acid: Known for Almost a Century, but Still in Vogue. Pharmaceutics.

[41] Kim J., Seki E. (2023). Hyaluronan in Liver Fibrosis: Basic Mechanisms, Clinical Implications, and Therapeutic Targets. Hepatol. Commun..

[42] Dhillon P., Carroll M., Cheng H. (2026). Tumorigenesis and Tumor Microenvironment in Lung Cancer. Curr. Issues Mol. Biol..

[43] Nail H.M., Chiu C.C., Leung C.H., Ahmed M.M.M., Wang H.M.D. (2023). Exosomal MiRNA-Mediated Intercellular Communications and Immunomodulatory Effects in Tumor Microenvironments. J. Biomed. Sci..

[44] Zhuo S., Yang S., Chen S., Ding Y., Cheng H., Yang L., Wang K., Yang K. (2024). Unveiling the Significance of Cancer-Testis Antigens and Their Implications for Immunotherapy in Glioma. Discov. Oncol..

[45] He C., Li L., Wang L., Meng W., Hao Y., Zhu G. (2021). Exosome-Mediated Cellular Crosstalk within the Tumor Microenvironment upon Irradiation. Cancer Biol. Med..

[46] Bao L., You B., Shi S., Shan Y., Zhang Q., Yue H., Zhang J., Zhang W., Shi Y., Liu Y. (2018). Metastasis-Associated MiR-23a from Nasopharyngeal Carcinoma-Derived Exosomes Mediates Angiogenesis by Repressing a Novel Target Gene TSGA10. Oncogene.

[47] Serafim R.B., da Silva P., Cardoso C., Di Cristofaro L.F.M., Netto R.P., de Almeida R., Navegante G., Storti C.B., de Sousa J.F., de Souza F.C. (2021). Expression Profiling of Glioblastoma Cell Lines Reveals Novel Extracellular Matrix-Receptor Genes Correlated with the Responsiveness of Glioma Patients to Ionizing Radiation. Front. Oncol..

[48] Zhang Y., Xie R., Zhang H., Zheng Y., Lin C., Yang L., Huang M., Li M., Song F., Lu L. (2021). Integrin Β7 Inhibits Colorectal Cancer Pathogenesis via Maintaining Antitumor Immunity. Cancer Immunol. Res..

[49] Zhou D., Zhong C., Yang Q., Cui B., Wang Y. (2025). Integration of Bulk and Single-Cell Transcriptomic Data Reveals a Novel Signature Related to Liver Metastasis and Basement Membrane in Pancreatic Cancer. Front. Immunol..

[50] Roy Choudhury S., Byrum S.D., Alkam D., Ashby C., Zhan F., Tackett A.J., Van Rhee F. (2023). Expression of Integrin β-7 Is Epigenetically Enhanced in Multiple Myeloma Subgroups with High-Risk Cytogenetics. Clin. Epigenet..

[51] Wang Y., Yuan D., Zhou L., Liang Z., Zhou W., Lu J., Jiang B., You L., Guo J., Zhao Y.P. (2020). Transducin-Like Enhancer of Split-1 Inhibits Malignant Behaviors in Vitro and Predicts a Better Prognosis in Pancreatic Ductal Adenocarcinoma. Front. Oncol..

[52] Sun Q., Ye Z., Qin Y., Fan G., Ji S., Zhuo Q., Xu W., Liu W., Hu Q., Liu M. (2020). Oncogenic Function of TRIM2 in Pancreatic Cancer by Activating ROS-Related NRF2/ITGB7/FAK Axis. Oncogene.

[53] Radke J., Schumann E., Onken J., Koll R., Acker G., Bodnar B., Senger C., Tierling S., Möbs M., Vajkoczy P. (2022). Decoding Molecular Programs in Melanoma Brain Metastases. Nat. Commun..

[54] Muntiu A., Moresi F., Vincenzoni F., Rossetti D.V., Iavarone F., Messana I., Castagnola M., La Rocca G., Mazzucchi E., Olivi A. (2024). Proteomic Profiling of Pre- and Post-Surgery Saliva of Glioblastoma Patients: A Pilot Investigation. Int. J. Mol. Sci..

[55] Zykova T., Zhu F., Wang L., Li H., Lim D.Y., Yao K., Roh E., Yoon S.P., Kim H.G., Bae K.B. (2018). Targeting PRPK Function Blocks Colon Cancer Metastasis. Mol. Cancer Ther..

[56] Abe Y., Takeuchi T., Imai Y., Murase R., Kamei Y., Fujibuchi T., Matsumoto S., Ueda N., Ogasawara M., Shigemoto K. (2006). A Small Ras-like Protein Ray/Rab1c Modulates the P53-Regulating Activity of PRPK. Biochem. Biophys. Res. Commun..

[57] Suárez-Arroyo I.J., Feliz-Mosquea Y.R., Pérez-Laspiur J., Arju R., Giashuddin S., Maldonado-Martínez G., Cubano L.A., Schneider R.J., Martínez-Montemayor M.M. (2016). The Proteome Signature of the Inflammatory Breast Cancer Plasma Membrane Identifies Novel Molecular Markers of Disease. Am. J. Cancer Res..

[58] Wheeler D.B., Zoncu R., Root D.E., Sabatini D.M., Sawyers C.L. (2015). Identification of an Oncogenic RAB Protein. Science.

[59] Homma Y., Hiragi S., Fukuda M. (2020). Rab Family of Small GTPases: An Updated View on Their Regulation and Functions. FEBS J..

[60] Montanari J., Schwob L., Marie-Brasset A., Vinatier C., Lepleux C., Antoine R., Guicheux J., Poully J.C., Chevalier F. (2024). Pilot Screening of Potential Matrikines Resulting from Collagen Breakages through Ionizing Radiation. Radiat. Environ. Biophys..

[61] Yang H., Cui Y., Zhu Y.M. (2023). Comprehensive Analysis Reveals Signal and Molecular Mechanism of Mitochondrial Energy Metabolism Pathway in Pancreatic Cancer. Front. Genet..

[62] Kashirina D., Matveeva D., Ezdakova M., Brzhozovskiy A., Kononikhin A., Pastushkova L., Larina I., Nikolaev E., Buravkova L., Ratushnyy A. (2025). Influence of Stress-Induced Senescence on the Secretome of Primary Mesenchymal Stromal Cells. Biomolecules.

[63] Chen H., Wang Z., Sun C., Zhong Y., Liu Y., Li Y., Zhang T., Zhang Y., Zhu X., Li L. (2025). MALMPS: A Machine Learning-Based Metabolic Gene Prognostic Signature for Stratifying Clinical Outcomes and Molecular Heterogeneity in Stage II/III Colorectal Cancer. Adv. Sci..

[64] Liu S., Imani S., Deng Y., Pathak J.L., Wen Q., Chen Y., Wu J. (2020). Targeting IFN/STAT1 Pathway as a Promising Strategy to Overcome Radioresistance. Onco. Targets. Ther..

[65] Zaganelli S., Rebelo-Guiomar P., Maundrell K., Rozanska A., Pierredon S., Powell C.A., Jourdain A.A., Hulo N., Lightowlers R.N., Chrzanowska-Lightowlers Z.M. (2017). The Pseudouridine Synthase RPUSD4 Is an Essential Component of Mitochondrial RNA Granules. J. Biol. Chem..

[66] Yu R., Wei Y., He C., Zhou P., Yang H., Deng C., Liu R., Wu P., Gao Q., Cao C. (2022). Integrative Analyses of M6A Regulators Identify That METTL3 Is Associated with HPV Status and Immunosuppressive Microenvironment in HPV-Related Cancers. Int. J. Biol. Sci..

[67] Zhou X., Ling Y., Cui J., Wang X., Long N., Teng W., Liu J., Xiang X., Yang H., Chu L. (2024). Mitochondrial RNA Modification-Based Signature to Predict Prognosis of Lower Grade Glioma: A Multi-Omics Exploration and Verification Study. Sci. Rep..

[68] Zheng P., Long Z., Gao A., Lu J., Wang S., Zhong C., Lai H., Guo Y., Wang K., Fang C. (2023). A Five-Pseudouridylation-Associated-LncRNA Classifier for Primary Prostate Cancer Prognosis Prediction. Front. Genet..

[69] Yang T., Sun J., Wang W., Li D., Yang X., Jia A., Ma Y., Fan Z. (2022). Hsa_circ_0006732 Regulates Colorectal Cancer Cell Proliferation, Invasion and EMT by MiR-127-5p/RAB3D Axis. Mol. Cell. Biochem..

[70] Ashrafizadeh M., Dai J., Torabian P., Nabavi N., Aref A.R., Aljabali A.A.A., Tambuwala M., Zhu M. (2024). Circular RNAs in EMT-Driven Metastasis Regulation: Modulation of Cancer Cell Plasticity, Tumorigenesis and Therapy Resistance. Cell. Mol. Life Sci..

[71] Ma J., Li Q., Li Y. (2022). CircRNA PRH1-PRR4 Stimulates RAB3D to Regulate the Malignant Progression of NSCLC by Sponging MiR-877-5p. Thorac. Cancer.

[72] Zhang J., Kong R., Sun L. (2017). Silencing of Rab3D Suppresses the Proliferation and Invasion of Esophageal Squamous Cell Carcinoma Cells. Biomed. Pharmacother..

[73] Stenmark H. (2009). Rab GTPases as Coordinators of Vesicle Traffic. Nat. Rev. Mol. Cell Biol..

[74] Li C., Lin X., Zhang C., Wan L., Yin J., Wang B. (2021). Long Non-Coding RNA FGD5-AS1 Enhances Osteosarcoma Cell Proliferation and Migration by Targeting MiR-506-3p/RAB3D Axis. Hum. Cell.

[75] Xie J., Zheng Y., Xu X., Sun C., Lv M. (2020). Long Noncoding RNA CAR10 Contributes to Melanoma Progression By Suppressing MiR-125b-5p to Induce RAB3D Expression. Onco. Targets. Ther..

[76] Yang J., Liu W., Lu X., Fu Y., Li L., Luo Y. (2015). High Expression of Small GTPase Rab3D Promotes Cancer Progression and Metastasis. Oncotarget.

[77] Ferreira A., Castanheira P., Escrevente C., Barral D.C., Barona T. (2024). Membrane Trafficking Alterations in Breast Cancer Progression. Front. Cell Dev. Biol..

[78] Wu X., Chen H., Liu N., Liu S., Lin G. (2022). Curcumin Suppresses Lung Cancer Progression via CircRUNX1 Mediated MiR-760/RAB3D Axis. Thorac. Cancer.

[79] Xu Y., Ren J., He X., Chen H., Wei T., Feng W. (2019). YWHA/14-3-3 Proteins Recognize Phosphorylated TFEB by a Noncanonical Mode for Controlling TFEB Cytoplasmic Localization. Autophagy.

[80] Hu X., Bao M., Huang J., Zhou L., Zheng S. (2020). Identification and Validation of Novel Biomarkers for Diagnosis and Prognosis of Hepatocellular Carcinoma. Front. Oncol..

[81] Sibai M., Parlayan C., Tuğlu P., Öztürk G., Demircan T. (2019). Integrative Analysis of Axolotl Gene Expression Data from Regenerative and Wound Healing Limb Tissues. Sci. Rep..

[82] Zhou Y., Liu S., Luo Y., Zhang M., Jiang X., Xiong Y. (2020). IncRNA MAPKAPK5-AS1 Promotes Proliferation and Migration of Thyroid Cancer Cell Lines by Targeting MiR-519e-5p/YWHAH. Eur. J. Histochem..

[83] Mao Z., Zhong L., Zhuang X., Liu H., Peng Y. (2022). Curcumenol Targeting YWHAG Inhibits the Pentose Phosphate Pathway and Enhances Antitumor Effects of Cisplatin. Evid. Based Complement. Altern. Med..

[84] Li M., Zhai G., Gu X., Sun K. (2018). ATF3 and PRAP1 Play Important Roles in Cisplatin-Induced Damages in Microvascular Endothelial Cells. Gene.

[85] Yavuz M., Şahin B., Baykal A.T., Demircan T. (2023). Hydroquinidine Displays a Significant Anticarcinogenic Activity in Breast and Ovarian Cancer Cells via Inhibiting Cell-Cycle and Stimulating Apoptosis. Turk. J. Biol..

[86] Grandt C.L., Brackmann L.K., Foraita R., Schwarz H., Hummel-Bartenschlager W., Hankeln T., Kraemer C., Zahnreich S., Drees P., Mirsch J. (2023). Gene Expression Variability in Long-Term Survivors of Childhood Cancer and Cancer-Free Controls in Response to Ionizing Irradiation. Mol. Med..

[87] Thomsen H., Chen B., Figlioli G., Elisei R., Romei C., Cipollini M., Cristaudo A., Bambi F., Hoffmann P., Herms S. (2016). Runs of Homozygosity and Inbreeding in Thyroid Cancer. BMC Cancer.

[88] Conde-Muíño R., Cuadros M., Zambudio N., Segura-Jiménez I., Cano C., Palma P. (2015). Predictive Biomarkers to Chemoradiation in Locally Advanced Rectal Cancer. BioMed Res. Int..

[89] Wu B., Shen L., Peng G., Li Y., Zhou Z., Li J., Huang X., Zhou Q., Jiang H., Huang J. (2022). Molecular Characteristics of Pediatric Nasopharyngeal Carcinoma Using Whole-Exome Sequencing. Oral Oncol..

[90] Nikkhah O., Einollahi B., Asadi M., Heiat M., Hushmandi K. (2025). Gene Network Changes after Exposure to Nanoliposomes Containing Antisense MiR-21 and MiR-373 in Bladder Cancer Cells: An in vitro Model Study. Biochem. Biophys. Rep..

[91] Fujie T., Kobayashi M., Ikeuchi L., Nakano T., Kitabatake K., Shinoda Y., Fujiwara Y., Yamamoto C., Tsukimoto M., Kaji T. (2024). Transcriptome Analysis of Cultured Human Vascular Endothelial Cells after γ-Ray Irradiation and Correlation Analysis with ATP, ADP, and Adenosine as Secondary Soluble Factors. J. Toxicol. Sci..

[92] Beck M., Rombouts C., Moreels M., Aerts A.N., Quintens R., Tabury K., Michaux A., Janssen A., Neefs M., Ernst E. (2014). Modulation of Gene Expression in Endothelial Cells in Response to High LET Nickel Ion Irradiation. Int. J. Mol. Med..

[93] Otani Y., Yoo J.Y., Chao S., Liu J., Jaime-Ramirez A.C., Lee T.J., Hurwitz B., Yan Y., Dai H., Glorioso J.C. (2020). Oncolytic HSV-Infected Glioma Cells Activate NOTCH in Adjacent Tumor Cells Sensitizing Tumors to Gamma Secretase Inhibition. Clin. Cancer Res..

[94] Brandes D., Yasin L., Nebral K., Ebler J., Schinnerl D., Picard D., Bergmann A.K., Alam J., Köhrer S., Haas O.A. (2023). Optical Genome Mapping Identifies Novel Recurrent Structural Alterations in Childhood *ETV6::RUNX1+* and High Hyperdiploid Acute Lymphoblastic Leukemia. Hemasphere.

[95] Xi Y., Yuefen P., Wei W., Quan Q., Jing Z., Jiamin X., Shuwen H. (2019). Analysis of Prognosis, Genome, Microbiome, and Microbial Metabolome in Different Sites of Colorectal Cancer. J. Transl. Med..

[96] Lee Y.Q., Rajadurai P., Abas F., Othman I., Naidu R. (2021). Proteomic Analysis on Anti-Proliferative and Apoptosis Effects of Curcumin Analog, 1,5-Bis(4-Hydroxy-3-Methyoxyphenyl)-1,4-Pentadiene-3-One-Treated Human Glioblastoma and Neuroblastoma Cells. Front. Mol. Biosci..

[97] Zhang D., Hugo W., Bergsneider M., Wang M.B., Kim W., Vinters H.V., Heaney A.P. (2022). Single-Cell RNA Sequencing in Silent Corticotroph Tumors Confirms Impaired POMC Processing and Provides New Insights into Their Invasive Behavior. Eur. J. Endocrinol..

[98] Chen Z., Guo X., Tao R., Huyghe J.R., Law P.J., Fernandez-Rozadilla C., Ping J., Jia G., Long J., Li C. (2024). Fine-Mapping Analysis Including over 254,000 East Asian and European Descendants Identifies 136 Putative Colorectal Cancer Susceptibility Genes. Nat. Commun..

[99] Liu X.D., Xie D.F., Wang Y.L., Guan H., Huang R.X., Zhou P.K. (2018). Integrated Analysis of LncRNA-MRNA Co-Expression Networks in the α-Particle Induced Carcinogenesis of Human Branchial Epithelial Cells. Int. J. Radiat. Biol..

[100] Sui Y., Shen Z., Li X., Lu Y., Feng S.T., Ma R., Wu J., Jing C., Wang Z., Feng J. (2024). Rupatadine-Inhibited OTUD3 Promotes DLBCL Progression and Immune Evasion through Deubiquitinating MYL12A and PD-L1. Cell Death Dis..

[101] Tatara Y., Monzen S. (2023). Proteomics and Secreted Lipidomics of Mouse-Derived Bone Marrow Cells Exposed to a Lethal Level of Ionizing Radiation. Sci. Rep..

[102] Nishad S., Chauhan P.K., Sowdhamini R., Ghosh A. (2021). Chronic Exposure of Humans to High Level Natural Background Radiation Leads to Robust Expression of Protective Stress Response Proteins. Sci. Rep..

[103] Juvkam I.S., Zlygosteva O., Sitarz M., Thiede B., Sørensen B.S., Malinen E., Edin N.J., Søland T.M., Galtung H.K. (2023). Proton Compared to X-Irradiation Induces Different Protein Profiles in Oral Cancer Cells and Their Derived Extracellular Vesicles. Int. J. Mol. Sci..

[104] Shahreza R.M., Zare H., Rastegar-Pouyani N., Aria H., Hakim F., Arefnia M., Bakherad H. (2025). GRP-Based Vaccines as a Novel Approach in Cancer Immunotherapy: Mechanisms, Challenges, and Prospects. Cancer Cell Int..

[105] Quarta R., Boccanegra B., Cristiano E., Ladisa A., Conte E., Ohana J., Mouly V., De Luca A., Hildyard J., Cappellari O. (2026). Best Reference Genes for Unbiased Normalized Transcript Expression in Normal and Dystrophic Human Cell Models of Myogenesis. PLoS ONE.

[106] Castenetto A., Gagliano T. (2025). Epigenetics and the Tumor Microenvironment in Neuroendocrine Tumors. Cancers.

[107] Camicia R., Winkler H.C., Hassa P.O. (2015). Novel Drug Targets for Personalized Precision Medicine in Relapsed/Refractory Diffuse Large B-Cell Lymphoma: A Comprehensive Review. Mol. Cancer.

[108] Miao L., Holley A.K., Zhao Y., St. Clair W.H., St. Clair D.K. (2014). Redox-Mediated and Ionizing-Radiation-Induced Inflammatory Mediators in Prostate Cancer Development and Treatment. Antioxid. Redox Signal..

[109] Robert M., Kennedy B.K., Crasta K.C. (2024). Therapy-Induced Senescence through the Redox Lens. Redox Biol..

[110] Chandrasekar A.P., Cummins N.W., Badleya A.D. (2019). The Role of the BCL-2 Family of Proteins in HIV-1 Pathogenesis and Persistence. Clin. Microbiol. Rev..

[111] Akter S., Madhuvilakku R., Kar A.K., Nila I.S., Liu P., Inuzuka H., Wei W., Hong Y. (2026). Reactive Oxygen Species (ROS) in Cancer: From Mechanism to Therapeutic Implications. Signal Transduct. Target. Ther..

[112] Vitiello G.A.F., Ferreira W.A.S., Cordeiro de Lima V.C., Medina T.d.S. (2021). Antiviral Responses in Cancer: Boosting Antitumor Immunity Through Activation of Interferon Pathway in the Tumor Microenvironment. Front. Immunol..

[113] Tubin S., Gupta S., Grusch M., Popper H.H., Brcic L., Ashdown M.L., Khleif S.N., Peter-Vörösmarty B., Hyden M., Negrini S. (2020). Shifting the Immune-Suppressive to Predominant Immune-Stimulatory Radiation Effects by SBRT-PArtial Tumor Irradiation Targeting HYpoxic Segment (SBRT-PATHY). Cancers.

[114] Tubin S., Fossati P., Carlino A., Martino G., Gora J., Stock M., Hug E. (2022). Novel Carbon Ion and Proton Partial Irradiation of Recurrent Unresectable Bulky Tumors (Particle-PATHY): Early Indication of Effectiveness and Safety. Cancers.

