# Supplementary material for: Proton-Induced Bystander Signaling Drives Cell-Type-Specific Proteomic Remodeling in Chondrocytes and Endothelial Cells
Source: Cancers (Basel). 2026 Nov 3;18(21):3534. doi: 10.3390/cancers18213534 (PMC13762256; doi:10.3390/cancers18213534)
Supplement: Supplementary file 1 [file cancers-18-03534-s001.zip › cancers-4624314-supplementary.pdf]

Table S1. Proteins differentially abundant in T/C-28A2 chondrocytes exposed to conditioned medium (CM) from SW1353 cells irradiated with 0.1 Gy protons, compared with sham-CM (n = 3 per group; q < 0.05 and |FC| ≥ 1.5; 85 proteins).

Identifier	UniProt accessions (protein group)	Gene name(s)	Protein name	log2FC	Direction	q-value	Valid values sham-CM / irradiated-CM (n/3)
A0A0D9SFB1	A0A0D9SFB1;H7C5U0	DNM1	dynamin 1	2.31	increased	1.9E-04	1 / 2
P52926	P52926	HMGA2	high mobility group AT-hook 2	1.99	increased	4.3E-02	3 / 3
Q8N4H5	Q8N4H5	TOMM5	translocase of outer mitochondrial membrane 5	1.81	increased	2.5E-03	3 / 3
Q9BTV5	Q9BTV5	FSD1	fibronectin type III and SPRY domain containing 1	1.58	increased	2.2E-06	2 / 1
Q9H5U6	Q9H5U6	ZCCHC4	zinc finger CCHC-type containing 4	1.53	increased	4.8E-03	1 / 3
P52657	P52657	GTF2A2	general transcription factor IIA subunit 2	1.47	increased	2.8E-05	2 / 3
F8VXL3	F8VXL3;F8VZU9;F8W180;G8J LA 2	MYL6	myosin light chain 6	1.42	increased	7.9E-05	3 / 3
O75223	O75223	GGCT	gamma-glutamylcyclotransferase	1.39	increased	1.2E-02	3 / 3
P0C5J1 †	P0C5J1;Q8N7N1	FAM86B1;FAM86B2	family with sequence similarity 86 member B2	1.38	increased	2.6E-13	0 / 2
Q9P266	Q9P266	JCAD	junctional cadherin 5 associated	1.36	increased	1.3E-03	3 / 3
B7Z613	B7Z613	GPM6B	glycoprotein M6B	1.33	increased	2.1E-02	3 / 2
Q8IYU8	Q8IYU8	MICU2	mitochondrial calcium uptake 2	1.26	increased	3.7E-02	2 / 3
P51793	P51793	CLCN4	chloride voltage-gated channel 4	1.22	increased	3.3E-04	3 / 1
Q7L211	Q7L211	ABHD13	abhydrolase domain containing 13	1.12	increased	3.2E-07	3 / 3
Q92738	Q92738	USP6NL	USP6 N-terminal like	1.10	increased	1.7E-02	3 / 3
Q96BQ5	Q96BQ5	CCDC127	coiled-coil domain containing 127	1.09	increased	4.9E-03	2 / 3
Q8IWR0.1	Q8IWR0;Q9UGR2	ZC3H7A;ZC3H7B	zinc finger CCCH-type containing 7A	1.03	increased	5.3E-10	3 / 3
O75197.1	O75197;O75581	LRP5;LRP6	LDL receptor related protein 5	1.01	increased	2.7E-02	3 / 3
Q9Y535	Q9Y535	POLR3H	RNA polymerase III subunit H	1.00	increased	4.0E-02	3 / 3
A0A804HII9	A0A804HII9;A0A804HIN7;A0A8 04HJU8;A0A804HKE2;A0A8 04H LD0	ACTN1	actinin alpha 1	0.99	increased	1.3E-02	3 / 3
Q15695	Q15695;Q15696	ZRSR2;ZRSR2P1	zinc finger CCCH-type, RNA binding motif and serine/arginine rich 2	0.93	increased	1.1E-03	3 / 3
Q9H9G7	Q9H9G7	AGO3	argonaute RISC catalytic component 3	0.92	increased	6.1E-03	3 / 3
Q9GZM7	Q9GZM7	TINAGL1	tubulointerstitial nephritis antigen like 1	0.88	increased	2.0E-02	3 / 3
Q8TAG9.1	Q8TAG9;Q9Y2D4	EXOC6;EXOC6B	exocyst complex component 6	0.84	increased	2.1E-02	3 / 3
P98172	P98172	EFNB1	ephrin B1	0.83	increased	2.8E-02	3 / 3
Q8N960	Q8N960	CEP120	centrosomal protein 120	0.81	increased	1.3E-02	3 / 3

Identifier	UniProt accessions (protein group)	Gene name(s)	Protein name	log2FC	Direction	q-value	Valid values sham-CM / irradiated-CM (n/3)
Q9Y6M9	Q9Y6M9	NDUFB9	NADH:ubiquinone oxidoreductase subunit B9	0.78	increased	1.2E-02	3 / 3
A0A2R8YEA7	A0A2R8YEA7	ACTB	actin beta	0.76	increased	2.7E-02	3 / 3
F6TLX2	F6TLX2;I3L3Q4	GLOD4	glyoxalase domain containing 4	0.74	increased	1.8E-02	3 / 3
Q9UPM9	Q9UPM9	B9D1	B9 domain containing 1	0.74	increased	2.3E-02	3 / 3
Q15672	Q15672;Q8WVJ9	TWIST1;TWIST2	twist family bHLH transcription factor 1	0.73	increased	4.4E-02	3 / 3
Q6P4Q7.1	Q6P4Q7;Q9H8M5	CNNM2;CNNM4	cyclin and CBS domain divalent metal cation transport mediator 4	0.72	increased	1.4E-02	3 / 3
Q6NT76	Q6NT76	HMBOX1	homeobox containing 1	0.69	increased	2.2E-02	2 / 3
Q8N543	Q8N543	OGFOD1	2-oxoglutarate and iron dependent oxygenase domain containing 1	0.69	increased	2.3E-02	3 / 3
Q86YT6	Q86YT6	MIB1	MIB E3 ubiquitin protein ligase 1	0.68	increased	3.0E-04	3 / 3
Q92698	Q92698	RAD54L	RAD54 like	0.68	increased	3.8E-03	2 / 1
Q8IWR0	Q8IWR0	ZC3H7A	zinc finger CCCH-type containing 7A	0.67	increased	2.4E-02	3 / 3
Q8N300	Q8N300	SVBP	small vasohibin binding protein	0.66	increased	4.8E-02	3 / 3
O75496	O75496	GMNN	geminin DNA replication inhibitor	0.66	increased	1.5E-02	3 / 3
P19105	P19105	MYL12A	myosin light chain 12A	0.65	increased	2.7E-02	2 / 3
P54252	P54252	ATXN3	ataxin 3	0.64	increased	1.1E-03	3 / 3
Q7Z6J9	Q7Z6J9	TSEN54	tRNA splicing endonuclease subunit 54	0.64	increased	3.9E-02	2 / 3
P26378	P26378;Q12926;Q14576	ELAVL2;ELAVL3;ELAVL4	ELAV like RNA binding protein 4	0.63	increased	2.8E-02	3 / 3
Q96JP5	Q96JP5	ZFP91	ZFP91 zinc finger protein, atypical E3 ubiquitin ligase	0.62	increased	1.7E-02	3 / 3
P51828	P51828	ADCY7	adenylate cyclase 7	0.62	increased	1.3E-02	3 / 3
Q969Z3	Q969Z3	MTARC2	mitochondrial amidoxime reducing component 2	0.62	increased	3.2E-03	3 / 3
Q9H974	Q9H974	QTRT2	queuine tRNA-ribosyltransferase accessory subunit 2	0.61	increased	4.6E-02	3 / 3
Q9NQY0	Q9NQY0	BIN3	bridging integrator 3	0.60	increased	4.3E-02	3 / 3
Q5JSP0	Q5JSP0	FGD3	FYVE, RhoGEF and PH domain containing 3	0.60	increased	7.4E-03	3 / 3
P14854	P14854	COX6B1	cytochrome c oxidase subunit 6B1	0.59	increased	9.4E-03	3 / 3
P37275	P37275	ZEB1	zinc finger E-box binding homeobox 1	-0.59	decreased	3.2E-05	3 / 3
Q969F9	Q969F9	HPS3	HPS3 biogenesis of lysosomal organelles complex 2 subunit 1	-0.61	decreased	1.9E-06	3 / 3
Q86VQ1	Q86VQ1	GLCCI1	glucocorticoid induced 1	-0.61	decreased	2.6E-02	3 / 3
Q7L8J4	Q7L8J4	SH3BP5L	SH3 binding domain protein 5 like	-0.64	decreased	1.5E-08	3 / 3

Identifier	UniProt accessions (protein group)	Gene name(s)	Protein name	log2FC	Direction	q-value	Valid values sham-CM / irradiated-CM (n/3)
Q14168.1	Q14168;Q9NZW5	MPP2;PALS2	MAGUK p55 scaffold protein 2	-0.70	decreased	1.2E-02	3 / 3
Q9NWW5	Q9NWW5	CLN6	CLN6 transmembrane ER protein	-0.71	decreased	1.4E-04	3 / 3
P01111.1	P01111;P01112	HRAS;NRAS	NRAS proto-oncogene, GTPase	-0.73	decreased	2.3E-02	3 / 3
Q8TER0	Q8TER0	SNED1	sushi, nidogen and EGF like domains 1	-0.74	decreased	1.3E-02	3 / 2
P21580	P21580	TNFAIP3	TNF alpha induced protein 3	-0.74	decreased	2.2E-02	3 / 3
Q8NBN7	Q8NBN7	RDH13	retinol dehydrogenase 13	-0.81	decreased	1.3E-02	3 / 3
Q8IZ73 †	Q8IZ73	RPUSD2	RNA pseudouridine synthase domain containing 2	-0.82	decreased	2.6E-02	2 / 0
Q8IYH5	Q8IYH5	ZZZ3	zinc finger ZZ-type containing 3	-0.87	decreased	2.4E-03	3 / 3
P33981	P33981	TTK	TTK protein kinase	-0.93	decreased	1.4E-02	3 / 3
Q3SY17	Q3SY17;Q9H1U9	SLC25A51;SLC25A52	solute carrier family 25 member 52	-0.95	decreased	1.9E-02	3 / 3
Q92522	Q92522	H1-10	H1.10 linker histone	-0.96	decreased	3.6E-03	3 / 3
Q86X53	Q86X53	ERICH1	glutamate rich 1	-0.98	decreased	5.0E-02	3 / 3
P56524.1	P56524;Q8WUI4;Q9UQL6	HDAC4;HDAC5;HDAC7	histone deacetylase 4	-0.99	decreased	2.9E-02	3 / 3
Q16698	Q16698	DECR1	2,4-dienoyl-CoA reductase 1	-1.04	decreased	4.2E-02	3 / 3
O00459.1	O00459;P27986	PIK3R1;PIK3R2	phosphoinositide-3-kinase regulatory subunit 2	-1.07	decreased	8.2E-03	3 / 3
P50452	P50452	SERPINB8	serpin family B member 8	-1.10	decreased	2.6E-13	3 / 3
O95858	O95858	TSPAN15	tetraspanin 15	-1.15	decreased	4.6E-10	3 / 2
Q9Y4Y9	Q9Y4Y9	LSM5	LSM5 homolog, U6 small nuclear RNA and mRNA degradation associated	-1.19	decreased	2.8E-02	3 / 3
Q15628	Q15628	TRADD	TNFRSF1A associated via death domain	-1.21	decreased	3.6E-02	3 / 3
Q86WI1	Q86WI1;Q9UHN6	CEMIP2;PKHD1L1	PKHD1 like 1	-1.49	decreased	3.5E-06	3 / 2
A0A2R8YEJ5	A0A2R8YEJ5	LRGUK	leucine rich repeats and guanylate kinase domain containing	-1.49	decreased	1.6E-03	3 / 1
P48378	P48378;P48380	RFX2;RFX3	regulatory factor X2	-1.58	decreased	1.5E-06	3 / 1
P31152 †	P31152	MAPK4	mitogen-activated protein kinase 4	-1.61	decreased	2.9E-03	2 / 0
Q9NP58	Q9NP58	ABCB6	ATP binding cassette subfamily B member 6 (Langereis blood group)	-1.72	decreased	2.3E-06	3 / 1
O75874.1	O75874;P48735	IDH1;IDH2	isocitrate dehydrogenase (NADP(+)) 1	-1.79	decreased	8.5E-03	3 / 3
Q92618	Q92618	ZNF516	zinc finger protein 516	-1.90	decreased	2.6E-13	3 / 1
P26010 †	P26010	ITGB7	integrin subunit beta 7	-3.44	decreased	2.2E-02	2 / 0
Q9BZW7 †	Q9BZW7	TSGA10	testis specific 10	-4.05	decreased	2.6E-13	3 / 0
Q92928 †	Q92928	RAB1C	RAB1C, member RAS oncogene family (pseudogene)	-4.39	decreased	2.8E-02	2 / 0

Identifier	UniProt accessions (protein group)	Gene name(s)	Protein name	log2FC	Direction	q-value	Valid values sham-CM / irradiated-CM (n/3)
Q92839 †	Q92839	HAS1	hyaluronan synthase 1	-7.03	decreased	2.6E-13	3 / 0
Q93045 †	Q93045	STMN2	stathmin 2	-8.63	decreased	2.6E-13	3 / 0

log2FC = log2(mean abundance in irradiated-CM / mean abundance in sham-CM); positive values indicate higher abundance in bystander cells exposed to irradiated-CM.

q-value: DEP-adjusted p-value (fdrtool q-value) from the limma moderated t-test.

Identifier: leading UniProt accession of the protein group, as used in the text. Numerical suffixes (.1, .2, …) were added by the DEP software to distinguish protein groups sharing the same leading accession; they are not UniProt isoforms. The full protein group is given in the next column.

Protein name: name of the gene of the leading accession; when a protein group contains several genes, the peptides used for quantification are shared between these proteins.

† Detected in only one condition (≥ 2/3 replicates in one group, 0/3 in the other): the fold change depends on imputed values and should be read as presence/absence.

Table S2. Proteins differentially abundant in EA.hy926 endothelial cells exposed to conditioned medium (CM) from SW1353 cells irradiated with 2 Gy protons, compared with sham-CM (n = 3 per group; q < 0.05 and |FC| ≥ 1.5; 427 proteins).

Identifier	UniProt accessions (protein group)	Gene name(s)	Protein name	log2FC	Direction	q-value	Valid values sham-CM / irradiated-CM (n/3)
Q9BYZ2 †	Q9BYZ2	LDHAL6B	lactate dehydrogenase A like 6B	5.48	increased	2.3E-03	0 / 2
P27348.2 †	P27348;P31946;P61981;Q04917	YWHAB;YWHAG;YWHAH;Y WHAQ	tyrosine 3-monooxygenase/tryptophan 5-monooxygenase activation protein theta	4.72	increased	1.4E-02	0 / 2
O95716.4	O95716;P61224;P62834	RAB3D;RAP1A;RAP1B	RAB3D, member RAS oncogene family	3.81	increased	1.3E-03	1 / 3
P21108 †	P21108	PRPS1L1	phosphoribosyl pyrophosphate synthetase 1 like 1	3.76	increased	1.1E-05	0 / 2
P61129	P61129	ZC3H6	zinc finger CCCH-type containing 6	3.74	increased	9.4E-03	1 / 3
Q96CM3	Q96CM3	RPUSD4	RNA pseudouridine synthase D4	3.55	increased	9.9E-05	3 / 3
Q96JA3 †	Q96JA3	PLEKHA8	pleckstrin homology domain containing A8	2.96	increased	2.4E-13	0 / 3
Q8TDR0	Q8TDR0	TRAF3IP1	TRAF3 interacting protein 1	2.84	increased	3.1E-08	3 / 3
P10588.1	P10588;P10589;P24468	NR2F1;NR2F2;NR2F6	nuclear receptor subfamily 2 group F member 6	2.84	increased	1.4E-06	3 / 3
O95503	O95503	CBX6	chromobox 6	2.84	increased	8.9E-07	2 / 3
Q13114	Q13114	TRAF3	TNF receptor associated factor 3	2.79	increased	2.4E-13	1 / 3
P11171.1 †	P11171;Q9H4G0;Q9Y2J2	EPB41;EPB41L1;EPB41L3	erythrocyte membrane protein band 4.1	2.73	increased	2.4E-13	0 / 3
Q01432.1	Q01432;Q01433	AMPD2;AMPD3	adenosine monophosphate deaminase 3	2.44	increased	2.3E-04	1 / 3
Q5U5R9 †	Q5U5R9	HECTD2	HECT domain E3 ubiquitin protein ligase 2	2.25	increased	9.1E-05	0 / 2
Q96DT5	Q96DT5	DNAH11	dynein axonemal heavy chain 11	2.25	increased	4.8E-03	2 / 3
P0DPH7	P0DPH7;P0DPH8;Q6PEY2;Q71U 36	TUBA1A;TUBA3C;TUBA3D; TUBA3E	tubulin alpha 1a	2.18	increased	1.1 E-10	3 / 3
Q99643	Q99643	SDHC	succinate dehydrogenase complex subunit C	2.10	increased	1.2 E-02	2 / 2
Q09013 †	Q09013	DMPK	DM1 protein kinase	2.09	increased	4.8E-02	0 / 2
Q16739	Q16739	UGCG	UDP-glucose ceramide glucosyltransferase	2.09	increased	1.0E-06	3 / 3
Q8N6G5	Q8N6G5	CSGALNACT2	chondroitin sulfate N-acetylgalactosaminyltransferase 2	2.05	increased	2.1E-03	2 / 3
A0A5F9ZH79	A0A5F9ZH79;A0A5F9ZHP1	ASCC1	activating signal cointegrator 1 complex subunit 1	2.03	increased	5.9E-09	2 / 3
Q7RTR2	Q7RTR2	NLRC3	NLR family CARD domain containing 3	2.03	increased	8.2E-10	3 / 3
P20226.1	P20226;Q6SJ96	TBP;TBPL2	TATA-box binding protein	2.00	increased	4.9E-07	3 / 3
Q92504	Q92504	SLC39A7	solute carrier family 39 member 7	1.97	increased	1.0E-04	3 / 3

Identifier	UniProt accessions (protein group)	Gene name(s)	Protein name	log2FC	Direction	q-value	Valid values sham-CM / irradiated-CM (n/3)
Q8N6H7.1	Q8N6H7;Q9NP61	ARFGAP2;ARFGAP3	ADP ribosylation factor GTPase activating protein 2	1.94	increased	2.4E-13	3 / 3
Q9HCU9	Q9HCU9	BRMS1	BRMS1 transcriptional repressor and anoikis regulator	1.93	increased	1.7E-02	1 / 2
Q502W6	Q502W6	VWA3B	von Willebrand factor A domain containing 3B	1.87	increased	3.5E-03	1 / 3
Q5T0B9	Q5T0B9	ZNF362	zinc finger protein 362	1.86	increased	4.2E-02	2 / 2
Q9Y256	Q9Y256	RCE1	Ras converting CAAX endopeptidase 1	1.85	increased	1.2E-10	3 / 3
Q9H4E7	Q9H4E7	DEF6	DEF6 guanine nucleotide exchange factor	1.85	increased	1.6E-02	2 / 3
P51531.2	P51531;P51532;Q6ZRS2	SMARCA2;SMARCA4;SRCA P	SWI/SNF related, matrix associated, actin dependent regulator of chromatin, subfamily a, member 2	1.85	increased	2.0E-06	3 / 3
Q96NW7	Q96NW7	LRRC7	leucine rich repeat containing 7	1.83	increased	1.4E-05	2 / 2
Q8IUH8	Q8IUH8	SPPL2C	signal peptide peptidase like 2C	1.79	increased	2.4E-13	3 / 3
Q00987	Q00987	MDM2	MDM2 proto-oncogene	1.72	increased	4.2E-02	2 / 1
Q99759	Q99759	MAP3K3	mitogen-activated protein kinase kinase kinase 3	1.71	increased	4.7E-03	3 / 3
Q9NWS6	Q9NWS6	FAM118A	family with sequence similarity 118 member A	1.70	increased	2.3E-03	3 / 3
G9CGD6 †	G9CGD6	CNK3/IPCEF1	CNKSR family member 3	1.66	increased	1.0E-03	0 / 2
Q8WYL5	Q8WYL5	SSH1	slingshot protein phosphatase 1	1.65	increased	4.2E-06	1 / 3
A0A140T8Y5	A0A140T8Y5	DHX16	DEAH-box helicase 16	1.65	increased	3.5E-02	1 / 2
P31749.3	P31749;Q9Y243	AKT1;AKT3	AKT serine/threonine kinase 1	1.65	increased	7.4E-04	2 / 3
O43795.1	O43795;Q12965	MYO1B;MYO1E	myosin IB	1.64	increased	7.8E-07	2 / 3
Q6NZ63.1	Q6NZ63;Q9UHE8	STEAP1;STEAP1B	STEAP family member 1B	1.63	increased	4.9E-02	2 / 3
Q5VUA4	Q5VUA4	ZNF318	zinc finger protein 318	1.59	increased	1.3E-03	1 / 3
A0A804HJG7	A0A804HJG7;E7ERI8;E7EW49	CLASP2	cytoplasmic linker associated protein 2	1.59	increased	1.5E-05	2 / 3
Q96DU7	Q96DU7	ITPKC	inositol-trisphosphate 3-kinase C	1.58	increased	1.8E-02	2 / 2
Q15554	Q15554	TERF2	telomeric repeat binding factor 2	1.58	increased	1.7E-03	3 / 3
P07996.2	P07996;P35442;P49746	THBS1;THBS2;THBS3	thrombospondin 1	1.57	increased	3.6E-03	2 / 3
Q86VM9	Q86VM9	ZC3H18	zinc finger CCCH-type containing 18	1.56	increased	6.8E-03	2 / 2
Q96E29	Q96E29	MTERF3	mitochondrial transcription termination factor 3	1.55	increased	4.9E-02	2 / 3

Identifier	UniProt accessions (protein group)	Gene name(s)	Protein name	log2FC	Direction	q-value	Valid values sham-CM / irradiated-CM (n/3)
O43155	O43155	FLRT2	fibronectin leucine rich transmembrane protein 2	1.54	increased	4.4E-02	2 / 1
P00414	P00414	MT-CO3	COX3_HUMAN	1.54	increased	3.0E-02	1 / 2
Q01628.1	Q01628;Q01629	IFITM2;IFITM3	interferon induced transmembrane protein 3	1.54	increased	1.3E-05	3 / 3
A1XBS5	A1XBS5	CIBAR1	CBY1 interacting BAR domain containing 1	1.52	increased	3.5E-11	3 / 3
P28827.1	P28827;Q15262	PTPRK;PTPRM	protein tyrosine phosphatase receptor type M	1.52	increased	1.7E-06	3 / 3
Q9H813 †	Q9H813	PACC1	proton activated chloride channel 1	1.52	increased	3.1E-03	0 / 2
Q8IUE1	Q8IUE1	TGIF2LX	TGFB induced factor homeobox 2 like X-linked	1.51	increased	6.8E-03	2 / 2
Q86V85	Q86V85	GPR180	G protein-coupled receptor 180	1.50	increased	2.9E-12	3 / 3
A0A0U1RQQ2	A0A0U1RQQ2;A0A2R8YEE8;B5 MCL5;G5E975	SMARCB1	SWI/SNF related, matrix associated, actin dependent regulator of chromatin, subfamily b, member 1	1.49	increased	2.1E-05	3 / 3
O15379.1	O15379;Q13547;Q92769	HDAC1;HDAC2;HDAC3	histone deacetylase 3	1.47	increased	4.2E-07	3 / 3
P56211	P56211	ARPP19	cAMP regulated phosphoprotein 19	1.47	increased	7.0E-03	3 / 3
Q8TCY9	Q8TCY9	URGCP	upregulator of cell proliferation	1.47	increased	4.0E-02	2 / 3
Q9HCM2.1	Q9HCM2;Q9UIW2	PLXNA1;PLXNA4	plexin A4	1.43	increased	6.7E-05	2 / 3
Q96LT9	Q96LT9	RNPC3	RNA binding region (RNP1, RRM) containing 3	1.43	increased	1.2E-10	3 / 3
O75695	O75695	RP2	RP2 activator of ARL3 GTPase	1.42	increased	3.8E-02	1 / 2
Q6NSJ5.2	Q6NSJ5;Q8TDW0	LRRC8C;LRRC8E	leucine rich repeat containing 8 VRAC subunit E	1.41	increased	3.5E-04	3 / 3
Q13829	Q13829;Q9H3F6	KCTD10;TNFAIP1	TNF alpha induced protein 1	1.41	increased	9.2E-04	2 / 3
O15230.1	O15230;Q16787	LAMA3;LAMA5	laminin subunit alpha 5	1.37	increased	3.5E-03	1 / 3
O75290 †	O75290	ZNF780A	zinc finger protein 780A	1.37	increased	2.8E-03	2 / 0
P29972	P29972	AQP1	aquaporin 1 (Colton blood group)	1.37	increased	9.3E-07	2 / 2
Q6ZW31	Q6ZW31	SYDE1	synapse defective Rho GTPase homolog 1	1.37	increased	3.3E-04	3 / 3
P35579.1	P35579;P35580	MYH10;MYH9	myosin heavy chain 9	1.35	increased	3.8E-10	3 / 3
O60307	O60307	MAST3	microtubule associated serine/threonine kinase 3	1.34	increased	1.8E-03	3 / 3
P24390	P24390	KDELR1	KDEL endoplasmic reticulum protein retention receptor 1	1.34	increased	7.6E-04	3 / 3
Q9BSK0 †	Q9BSK0	MARVELD1	MARVEL domain containing 1	1.34	increased	2.1E-08	0 / 2

Identifier	UniProt accessions (protein group)	Gene name(s)	Protein name	log2FC	Direction	q-value	Valid values sham-CM / irradiated-CM (n/3)
O95258	O95258	SLC25A14	solute carrier family 25 member 14	1.33	increased	3.4E-03	1 / 2
Q96DE0.1	Q96DE0;Q9BRJ7	NUDT16;NUDT16L1	nudix hydrolase 16	1.33	increased	1.4E-03	3 / 3
P17026	P17026	ZNF22	zinc finger protein 22	1.32	increased	3.8E-13	2 / 1
Q96Q07	Q96Q07	BTBD9	BTB domain containing 9	1.32	increased	6.6E-04	2 / 2
P49842	P49842	STK19	serine/threonine kinase 19	1.31	increased	1.0E-02	1 / 2
Q9BU23	Q9BU23	LMF2	lipase maturation factor 2	1.31	increased	2.8E-02	3 / 3
O43731.1	O43731;P33947	KDELR2;KDELR3	KDEL endoplasmic reticulum protein retention receptor 3	1.30	increased	2.1E-10	3 / 2
P16520	P16520	GNB3	G protein subunit beta 3	1.30	increased	3.8E-02	2 / 1
O95271	O95271	TNKS	tankyrase	1.30	increased	2.4E-05	1 / 2
Q96E22	Q96E22	NUS1	NUS1 dehydrodolichyl diphosphate synthase subunit	1.29	increased	1.3E-05	2 / 3
Q7Z2E3	Q7Z2E3	APTX	aprataxin	1.29	increased	1.1E-03	1 / 2
P04156	P04156	PRNP	prion protein	1.27	increased	2.4E-03	1 / 2
Q9H4I9	Q9H4I9	SMDT1	single-pass membrane protein with aspartate rich tail 1	1.26	increased	7.4E-07	3 / 3
Q8WW01	Q8WW01	TSEN15	tRNA splicing endonuclease subunit 15	1.26	increased	4.5E-03	3 / 3
O95067	O95067	CCNB2	cyclin B2	1.26	increased	5.6E-03	3 / 3
P56199	P56199	ITGA1	integrin subunit alpha 1	1.25	increased	4.6E-02	3 / 3
P07205	P07205	PGK2	phosphoglycerate kinase 2	1.24	increased	1.0E-02	3 / 3
Q14596	Q14596	NBR1	NBR1 autophagy cargo receptor	1.24	increased	8.0E-06	2 / 3
P04350	P04350	TUBB4A	tubulin beta 4A class IVa	1.21	increased	1.6E-02	3 / 3
Q9HC78	Q9HC78	ZBTB20	zinc finger and BTB domain containing 20	1.21	increased	8.0E-05	3 / 3
Q96E52	Q96E52	OMA1	OMA1 zinc metallopeptidase	1.21	increased	2.6E-02	1 / 2
Q15831	Q15831	STK11	serine/threonine kinase 11	1.21	increased	8.4E-05	3 / 2
O95208.1	O95208;Q9H201	EPN2;EPN3	epsin 2	1.21	increased	4.3E-02	2 / 3
P08237.2	P08237;P17858;Q01813	PFKL;PFKM;PFKP	phosphofructokinase, muscle	1.20	increased	2.9E-05	3 / 3
P25054	P25054	APC	APC regulator of WNT signaling pathway	1.20	increased	1.6E-04	3 / 3
Q5SWX3	Q5SWX3	CAMK2G	calcium/calmodulin dependent protein kinase II gamma	1.17	increased	1.4E-03	3 / 3
Q86YN1	Q86YN1	DOLPP1	dolichyldiphosphatase 1	1.17	increased	3.4E-02	3 / 3
Q9UPS6	Q9UPS6	SETD1B	SET domain containing 1B, histone lysine methyltransferase	1.16	increased	1.7E-02	3 / 2
Q7L7X3.2	Q7L7X3;Q9H2K8;Q9UL54	TAOK1;TAOK2;TAOK3	TAO kinase 1	1.15	increased	4.2E-02	3 / 3
P48651	P48651	PTDSS1	phosphatidylserine synthase 1	1.15	increased	1.9E-02	3 / 3

Identifier	UniProt accessions (protein group)	Gene name(s)	Protein name	log2FC	Direction	q-value	Valid values sham-CM / irradiated-CM (n/3)
Q14435	Q14435	GALNT3	polypeptide N-acetylgalactosaminyltransferase 3	1.14	increased	4.7E-02	1 / 2
O60229	O60229	KALRN	kalirin RhoGEF kinase	1.14	increased	1.5E-02	1 / 2
O75417	O75417	POLQ	DNA polymerase theta	1.14	increased	2.5E-05	2 / 1
Q07955.1	Q07955;Q13242	SRSF1;SRSF9	serine and arginine rich splicing factor 1	1.13	increased	1.8E-04	3 / 3
Q8WV92	Q8WV92	MITD1	microtubule interacting and trafficking domain containing 1	1.12	increased	1.8E-03	3 / 3
Q15629	Q15629	TRAM1	translocation associated membrane protein 1	1.11	increased	3.2E-03	3 / 3
Q8N490	Q8N490	PNKD	PNKD metallo-beta-lactamase domain containing	1.11	increased	4.8E-02	3 / 3
Q9BX63	Q9BX63	BRIP1	BRCA1 interacting helicase 1	1.11	increased	2.6E-02	2 / 3
Q9BRU9	Q9BRU9	UTP23	UTP23 small subunit processome component	1.10	increased	1.4E-03	2 / 1
A2RTX5.1	A2RTX5;P26639	TARS1;TARS3	threonyl-tRNA synthetase 3	1.10	increased	3.0E-07	3 / 3
Q9Y2Y9	Q9Y2Y9	KLF13	KLF transcription factor 13	1.10	increased	7.7E-03	3 / 3
Q96A37	Q96A37	RNF166	ring finger protein 166	1.10	increased	5.2E-04	3 / 3
Q96P47.1	Q96P47;Q9UPQ3	AGAP1;AGAP3	ArfGAP with GTPase domain, ankyrin repeat and PH domain 3	1.10	increased	8.5E-03	3 / 3
A5A3E0.3	A5A3E0;P60709;P63261;P6326 7;Q562R	ACTB;ACTBL2;ACTG1;ACT G2;POTEF	POTE ankyrin domain family member F	1.10	increased	1.2E-04	3 / 3
Q92624	1 Q92624	APPBP2	amyloid beta precursor protein binding protein 2	1.10	increased	1.0E-06	3 / 3
Q96EC8	Q96EC8	YIPF6	Yip1 domain family member 6	1.10	increased	8.7E-06	3 / 3
A7E2Y1.1	A7E2Y1;P35579;P35580;P357 4 9	MYH10;MYH11;MYH7B ;M YH9	myosin heavy chain 7B	1.10	increased	1.4E-05	3 / 3
Q96IX5	Q96IX5	ATP5MK	ATP synthase membrane subunit k	1.09	increased	1.1E-05	3 / 3
P35579.3	P35579;P35749	MYH11;MYH9	myosin heavy chain 9	1.09	increased	3.9E-05	3 / 3
Q9H330	Q9H330	TMEM245	transmembrane protein 245	1.07	increased	2.5E-04	3 / 3
Q13227	Q13227	GPS2	G protein pathway suppressor 2	1.07	increased	4.7E-04	3 / 3
Q5BKT4	Q5BKT4	ALG10	ALG10 alpha-1,2-glucosyltransferase	1.07	increased	1.2E-04	3 / 3
P60673	P60673	PFN3	profilin 3	1.07	increased	1.8E-03	3 / 3
Q86WT1	Q86WT1	TTC30A	intraflagellar transport 70A	1.05	increased	4.2E-03	2 / 3
P42229	P42229	STAT5A	signal transducer and activator of transcription 5A	1.05	increased	3.8E-02	3 / 3

Identifier	UniProt accessions (protein group)	Gene name(s)	Protein name	log2FC	Direction	q-value	Valid values sham-CM / irradiated-CM (n/3)
P35579	P35579	MYH9	myosin heavy chain 9	1.05	increased	1.1E-05	3 / 3
Q14571.1	Q14571;Q14573	ITPR2;ITPR3	inositol 1,4,5-trisphosphate receptor type 2	1.05	increased	4.5E-03	3 / 3
Q6DKI1	Q6DKI1	RPL7L1	ribosomal protein L7 like 1	1.04	increased	2.6E-02	3 / 3
O75051.3	O75051;Q9UIW2	PLXNA1;PLXNA2	plexin A2	1.04	increased	2.4E-03	3 / 3
Q9Y2D2	Q9Y2D2	SLC35A3	solute carrier family 35 member A3	1.03	increased	2.3E-04	3 / 3
Q9UDW1	Q9UDW1	UQCR10	ubiquinol-cytochrome c reductase, complex III subunit X	1.02	increased	1.1E-03	3 / 3
P78383	P78383	SLC35B1	solute carrier family 35 member B1	1.01	increased	9.1E-03	3 / 3
P78364	P78364	PHC1	polyhomeotic homolog 1	1.01	increased	6.7E-03	2 / 3
O00507	O00507	USP9Y	ubiquitin specific peptidase 9 Y-linked	1.00	increased	4.8E-02	3 / 3
Q9NWZ3	Q9NWZ3	IRAK4	interleukin 1 receptor associated kinase 4	0.98	increased	1.1E-02	3 / 3
Q9NP80	Q9NP80	PNPLA8	patatin like phospholipase domain containing 8	0.98	increased	1.4E-03	3 / 3
Q5TC82.1	Q5TC82;Q9HBD1	RC3H1;RC3H2	ring finger and CCCH-type domains 1	0.98	increased	1.3E-02	3 / 3
Q9H4A5.1	Q9H4A5;Q9H4A6	GOLPH3;GOLPH3L	golgi phosphoprotein 3 like	0.98	increased	5.2E-03	3 / 3
P24844	P24844	MYL9	myosin light chain 9	0.96	increased	2.9E-05	3 / 3
O14965	O14965	AURKA	aurora kinase A	0.96	increased	6.3E-04	3 / 3
P11908	P11908	PRPS2	phosphoribosyl pyrophosphate synthetase 2	0.95	increased	1.4E-07	3 / 3
O95625	O95625	ZBTB11	zinc finger and BTB domain containing 11	0.94	increased	2.9E-02	3 / 3
P35579.2	P35579;P35580;P35749	MYH10;MYH11;MYH9	myosin heavy chain 9	0.94	increased	4.7E-04	3 / 3
P47755.1	P47755;P52907	CAPZA1;CAPZA2	capping actin protein of muscle Z-line subunit alpha 2	0.94	increased	2.4E-04	3 / 3
Q9H7P9 †	Q9H7P9	PLEKHG2	pleckstrin homology and RhoGEF domain containing G2	0.94	increased	2.9E-02	0 / 3
Q9H1H9	Q9H1H9	KIF13A	kinesin family member 13A	0.94	increased	4.8E-02	3 / 3
Q6NTF9	Q6NTF9	RHBDD2	rhomboid domain containing 2	0.93	increased	5.7E-04	2 / 3
Q8IYB5.1	Q8IYB5;Q8WU79	SMAP1;SMAP2	small ArfGAP 1	0.93	increased	3.9E-03	3 / 3
J3KTD8	J3KTD8	CBFB	core-binding factor subunit beta	0.93	increased	4.0E-05	3 / 3
P57088	P57088	TMEM33	transmembrane protein 33	0.92	increased	3.6E-03	3 / 3
P10589.1	P10589;P24468	NR2F1;NR2F2	nuclear receptor subfamily 2 group F member 1	0.92	increased	3.4E-02	3 / 3
Q86VX9	Q86VX9	MON1A	MON1 homolog A, secretory trafficking associated	0.92	increased	6.7E-05	3 / 3

Identifier	UniProt accessions (protein group)	Gene name(s)	Protein name	log2FC	Direction	q-value	Valid values sham-CM / irradiated-CM (n/3)
O14957	O14957	UQCR11	ubiquinol-cytochrome c reductase, complex III subunit XI	0.91	increased	1.8E-06	3 / 3
B3KU38	B3KU38;P0DPB3	IQCJ-SCHIP1;SCHIP1	IQCJ-SCHIP1 readthrough	0.91	increased	9.9E-04	3 / 3
Q99502	Q99502	EYA1	EYA transcriptional coactivator and phosphatase 1	0.90	increased	9.4E-03	2 / 1
P04049.1	P04049;P10398	ARAF;RAF1	Raf-1 proto-oncogene, serine/threonine kinase	0.90	increased	3.0E-02	3 / 3
Q9NVR5	Q9NVR5	DNAAF2	dynein axonemal assembly factor 2	0.90	increased	2.7E-02	1 / 2
Q9NVP2	Q9NVP2	ASF1B	anti-silencing function 1B histone chaperone	0.89	increased	6.8E-03	3 / 3
Q14563	Q14563	SEMA3A	semaphorin 3A	0.89	increased	4.1E-05	3 / 3
Q8TB03	Q8TB03	CXorf38	chromosome X open reading frame 38	0.88	increased	4.2E-04	3 / 3
Q9BQ04	Q9BQ04	RBM4B	RNA binding motif protein 4B	0.88	increased	1.6E-02	3 / 3
O94832	O94832	MYO1D	myosin ID	0.88	increased	8.4E-03	3 / 3
Q8N5M4	Q8N5M4	TTC9C	tetratricopeptide repeat domain 9C	0.88	increased	2.5E-02	3 / 3
Q9UBF6	Q9UBF6	RNF7	ring finger protein 7	0.88	increased	5.0E-03	3 / 3
P30291 †	P30291	WEE1	WEE1 G2 checkpoint kinase	0.87	increased	2.7E-04	2 / 0
Q9H920	Q9H920	RNF121	ring finger protein 121	0.87	increased	1.5E-04	3 / 3
Q6PFW1 †	Q6PFW1	PPIP5K1	diphosphoinositol pentakisphosphate kinase 1	0.86	increased	1.6E-03	0 / 2
Q69YN4	Q69YN4	VIRMA	vir like m6A methyltransferase associated	0.86	increased	7.2E-03	3 / 3
R4GN18	R4GN18	CD46	CD46 molecule	0.86	increased	8.0E-03	2 / 2
Q9Y6B7	Q9Y6B7	AP4B1	adaptor related protein complex 4 subunit beta 1	0.85	increased	8.6E-03	3 / 3
Q9GZT8	Q9GZT8	NIF3L1	NGG1 interacting factor 3 like 1	0.85	increased	9.1E-04	3 / 3
H7C4G8	H7C4G8	NT5DC2	5'-nucleotidase domain containing 2	0.85	increased	4.7E-02	1 / 2
P27348.3	P27348;P31946;P63104	YWHAB;YWHAQ;YWHAZ	tyrosine 3-monooxygenase/tryptophan 5-monooxygenase activation protein theta	0.84	increased	2.9E-03	3 / 3
Q8IUH5	Q8IUH5	ZDHHC17	zinc finger DHHC-type palmitoyltransferase 17	0.84	increased	1.7E-03	3 / 3
O00159	O00159	MYO1C	myosin IC	0.84	increased	2.5E-03	3 / 3
P07437.3	P07437;P68371;Q13885	TUBB;TUBB2A;TUBB4B	tubulin beta class I	0.84	increased	3.3E-02	3 / 3
A6NHQ2.1	A6NHQ2;P22087	FBL;FBLL1	fibrillarin	0.84	increased	3.2E-04	3 / 3
Q08AF3.1	Q08AF3;Q7Z7L1	SLFN11;SLFN5	schlafen family member 5	0.83	increased	2.7E-04	3 / 3
Q15631	Q15631	TSN	translin	0.83	increased	1.3E-04	3 / 3

Identifier	UniProt accessions (protein group)	Gene name(s)	Protein name	log2FC	Direction	q-value	Valid values sham-CM / irradiated-CM (n/3)
P19105	P19105	MYL12A	myosin light chain 12A	0.82	increased	7.6E-03	3 / 3
Q92994	Q92994	BRF1	BRF1 RNA polymerase III transcription initiation factor subunit	0.82	increased	4.8E-03	1 / 2
O60725	O60725	ICMT	isoprenylcysteine carboxyl methyltransferase	0.81	increased	2.3E-02	3 / 3
O95243	O95243	MBD4	methyl-CpG binding domain 4, DNA glycosylase	0.80	increased	2.5E-02	2 / 1
O15013.1	O15013;Q9HCE6	ARHGEF10;ARHGEF10L	Rho guanine nucleotide exchange factor 10	0.80	increased	2.1E-02	3 / 2
P26038.1	P26038;P35241	MSN;RDX	moesin	0.80	increased	4.4E-02	3 / 3
O95707	O95707	POP4	POP4 homolog, ribonuclease P/MRP subunit	0.79	increased	3.0E-02	3 / 3
P54753.1	P54753;P54760	EPHB3;EPHB4	EPH receptor B3	0.77	increased	7.2E-04	3 / 3
P49427	P49427	CDC34	cell division cycle 34, ubiqiutin conjugating enzyme	0.76	increased	1.2E-02	3 / 3
P45983.1	P45983;P45984	MAPK8;MAPK9	mitogen-activated protein kinase 8	0.76	increased	5.0E-03	3 / 3
A0AVF1	A0AVF1	TTC26	intraflagellar transport 56	0.76	increased	2.8E-04	3 / 3
Q99720	Q99720	SIGMAR1	sigma non-opioid intracellular receptor 1	0.76	increased	7.1E-04	3 / 3
Q09161	Q09161	NCBP1	nuclear cap binding protein subunit 1	0.76	increased	2.5E-03	3 / 3
Q96DX4	Q96DX4	RSPRY1	ring finger and SPRY domain containing 1	0.76	increased	2.5E-03	3 / 3
Q96CP6	Q96CP6	GRAMD1A	GRAM domain containing 1A	0.75	increased	2.3E-02	3 / 3
Q5T2E6	Q5T2E6	ARMH3	armadillo like helical domain containing 3	0.75	increased	4.8E-02	3 / 3
Q86YT6	Q86YT6	MIB1	MIB E3 ubiquitin protein ligase 1	0.75	increased	4.4E-02	3 / 3
P19634	P19634	SLC9A1	solute carrier family 9 member A1	0.74	increased	1.4E-03	3 / 3
P48507	P48507	GCLM	glutamate-cysteine ligase modifier subunit	0.73	increased	2.1E-02	3 / 3
Q9UK58	Q9UK58	CCNL1	cyclin L1	0.72	increased	3.8E-02	3 / 3
Q15173	Q15173	PPP2R5B	protein phosphatase 2 regulatory subunit B'beta	0.71	increased	1.5E-02	3 / 3
O43504	O43504	LAMTOR5	late endosomal/lysosomal adaptor, MAPK and MTOR activator 5	0.70	increased	1.2E-02	3 / 3
P61619	P61619	SEC61A1	SEC61 translocon subunit alpha 1	0.70	increased	3.0E-02	3 / 3
O14495	O14495	PLPP3	phospholipid phosphatase 3	0.70	increased	3.2E-02	3 / 3
Q9NX61	Q9NX61	TMEM161A	transmembrane protein 161A	0.70	increased	2.4E-02	3 / 3
Q8N9I9	Q8N9I9	DTX3	deltex E3 ubiquitin ligase 3	0.70	increased	2.3E-03	3 / 3
Q15283	Q15283	RASA2	RAS p21 protein activator 2	0.70	increased	1.8E-02	3 / 3

Identifier	UniProt accessions (protein group)	Gene name(s)	Protein name	log2FC	Direction	q-value	Valid values sham-CM / irradiated-CM (n/3)
Q16280	Q16280	CNGA2	cyclic nucleotide gated channel subunit alpha 2	0.69	increased	3.1E-02	3 / 3
Q8IV50	Q8IV50	LYSMD2	LysM domain containing 2	0.68	increased	1.8E-02	3 / 3
Q03519	Q03519	TAP2	transporter 2, ATP binding cassette subfamily B member	0.68	increased	1.5E-02	2 / 2
Q8IXQ5	Q8IXQ5	KLHL7	kelch like family member 7	0.68	increased	1.2E-02	3 / 3
Q96HA7	Q96HA7	TONSL	tonsoku like, DNA repair protein	0.67	increased	2.9E-03	3 / 3
Q8WVF1	Q8WVF1	OSCP1	organic solute carrier partner 1	0.67	increased	6.7E-03	3 / 3
O95989	O95989	NUDT3	nudix hydrolase 3	0.67	increased	4.1E-02	3 / 3
P00395	P00395	MT-CO1	COX1_HUMAN	0.67	increased	2.8E-03	2 / 3
Q7Z3V4	Q7Z3V4	UBE3B	ubiquitin protein ligase E3B	0.65	increased	2.8E-02	3 / 3
Q9BZ23.1	Q9BZ23;Q9H999	PANK2;PANK3	pantothenate kinase 2	0.65	increased	1.4E-02	3 / 3
Q9H9G7.3	Q9H9G7;Q9UL18	AGO1;AGO3	argonaute RISC catalytic component 3	0.65	increased	1.5E-03	3 / 3
Q14678.1	Q14678;Q63ZY3	KANK1;KANK2	KN motif and ankyrin repeat domains 1	0.64	increased	1.7E-02	3 / 3
O43660	O43660	PLRG1	pleiotropic regulator 1	0.64	increased	1.5E-03	3 / 3
O14735	O14735	CDIPT	CDP-diacylglycerol--inositol 3-phosphatidyltransferase	0.64	increased	1.6E-02	3 / 3
Q9NRX1	Q9NRX1	PNO1	partner of NOB1 homolog	0.63	increased	3.5E-02	3 / 3
O95619	O95619	YEATS4	YEATS domain containing 4	0.62	increased	2.8E-02	3 / 3
O60870	O60870	KIN	Kin17 DNA and RNA binding protein	0.62	increased	4.4E-03	3 / 3
A0A087WZL0	A0A087WZL0	PHLDB1	pleckstrin homology like domain family B member 1	0.62	increased	3.6E-02	3 / 3
P05976	P05976;P08590	MYL1;MYL3	myosin light chain 1	0.61	increased	4.1E-03	3 / 3
Q8WUA8	Q8WUA8	TSKU	tsukushi, small leucine rich proteoglycan	0.61	increased	6.1E-03	3 / 3
Q8IY33	Q8IY33;Q8N3F8	MICALL1;MICALL2	MICAL like 2	0.60	increased	4.5E-02	3 / 3
O15228	O15228	GNPAT	glyceronephosphate O-acyltransferase	0.60	increased	7.6E-03	3 / 3
Q8TEL6	Q8TEL6	TRPC4AP	transient receptor potential cation channel subfamily C member 4 associated protein	0.60	increased	3.7E-02	3 / 3
Q96M27	Q96M27	PRRC1	proline rich coiled-coil 1	0.60	increased	2.1E-02	3 / 3
Q75QN2	Q75QN2	INTS8	integrator complex subunit 8	0.60	increased	7.5E-03	3 / 3
Q9Y3R5	Q9Y3R5	DOP1B	DOP1 leucine zipper like protein B	0.60	increased	2.0E-02	3 / 3
P20674	P20674	COX5A	cytochrome c oxidase subunit 5A	0.60	increased	1.2E-02	3 / 3
P24539	P24539	ATP5PB	ATP synthase peripheral stalk-membrane subunit b	0.59	increased	2.7E-02	3 / 3
Q8NDI1	Q8NDI1	EHBP1	EH domain binding protein 1	-0.59	decreased	4.7E-02	3 / 3

Identifier	UniProt accessions (protein group)	Gene name(s)	Protein name	log2FC	Direction	q-value	Valid values sham-CM / irradiated-CM (n/3)
O95382	O95382	MAP3K6	mitogen-activated protein kinase kinase kinase 6	-0.59	decreased	4.2E-02	3 / 3
Q9BT30	Q9BT30	ALKBH7	alkB homolog 7	-0.59	decreased	3.7E-02	2 / 1
Q8N8N7	Q8N8N7	PTGR2	prostaglandin reductase 2	-0.60	decreased	3.8E-02	3 / 3
O75608	O75608	LYPLA1	lysophospholipase 1	-0.60	decreased	1.4E-02	3 / 3
Q9Y2B9	Q9Y2B9	PKIG	cAMP-dependent protein kinase inhibitor gamma	-0.60	decreased	2.3E-02	3 / 3
P33240.1	P33240;Q9H0L4	CSTF2;CSTF2T	cleavage stimulation factor subunit 2	-0.60	decreased	2.1E-02	3 / 3
O43715	O43715	TRIAP1	TP53 regulated inhibitor of apoptosis 1	-0.61	decreased	6.8E-03	3 / 3
P54259	P54259	ATN1	atrophin 1	-0.61	decreased	2.3E-02	3 / 3
P35227	P35227	PCGF2	polycomb group ring finger 2	-0.62	decreased	1.7E-03	3 / 3
Q92688	Q92688	ANP32B	acidic nuclear phosphoprotein 32 family member B	-0.62	decreased	2.6E-02	3 / 3
Q6P1N0	Q6P1N0	CC2D1A	coiled-coil and C2 domain containing 1A	-0.62	decreased	3.9E-02	3 / 3
O75896	O75896	TUSC2	tumor suppressor 2, mitochondrial calcium regulator	-0.62	decreased	8.7E-03	3 / 3
Q9BSE4	Q9BSE4	HERPUD2	HERPUD family member 2	-0.62	decreased	1.3E-02	3 / 2
O43414	O43414	ERI3	ERI1 exoribonuclease family member 3	-0.63	decreased	2.1E-02	3 / 3
P04350.8	P04350;P68371;Q13509	TUBB3;TUBB4A;TUBB4B	tubulin beta 4A class IVa	-0.63	decreased	3.1E-02	3 / 3
A0JLT2	A0JLT2	MED19	mediator complex subunit 19	-0.64	decreased	3.4E-03	3 / 3
Q5T653	Q5T653	MRPL2	mitochondrial ribosomal protein L2	-0.65	decreased	3.9E-03	3 / 3
Q92800	Q92800	EZH1	enhancer of zeste 1 polycomb repressive complex 2 subunit	-0.65	decreased	3.1E-02	3 / 1
Q9NZZ3	Q9NZZ3	CHMP5	charged multivesicular body protein 5	-0.66	decreased	4.4E-02	3 / 3
O00425.2	O00425;Q9NZI8;Q9Y6M1	IGF2BP1;IGF2BP2;IGF2BP3	insulin like growth factor 2 mRNA binding protein 3	-0.67	decreased	3.3E-02	3 / 3
A0A2R8Y7C0	A0A2R8Y7C0	HBA2	hemoglobin subunit alpha 2	-0.68	decreased	1.5E-02	3 / 3
Q9BQE3	Q9BQE3	TUBA1C	tubulin alpha 1c	-0.68	decreased	3.7E-02	3 / 3
Q9HAN9	Q9HAN9	NMNAT1	nicotinamide nucleotide adenylyltransferase 1	-0.68	decreased	1.2E-02	3 / 3
P54725.1	P54725;P54727	RAD23A;RAD23B	RAD23 homolog A, nucleotide excision repair protein	-0.69	decreased	1.6E-02	3 / 3
O43633	O43633	CHMP2A	charged multivesicular body protein 2A	-0.70	decreased	6.2E-03	3 / 3
O75155.1	O75155;Q86VP6	CAND1;CAND2	cullin associated and neddylation dissociated 2 (putative)	-0.71	decreased	5.0E-02	3 / 3

Identifier	UniProt accessions (protein group)	Gene name(s)	Protein name	log2FC	Direction	q-value	Valid values sham-CM / irradiated-CM (n/3)
Q9H8H2	Q9H8H2	DDX31	DEAD-box helicase 31	-0.71	decreased	8.2E-04	3 / 3
Q9UK45	Q9UK45	LSM7	LSM7 homolog, U6 small nuclear RNA and mRNA degradation associated	-0.71	decreased	4.5E-02	3 / 3
P32322.1	P32322;Q96C36	PYCR1;PYCR2	pyrroline-5-carboxylate reductase 1	-0.71	decreased	2.0E-02	3 / 3
O95302.1	O95302;Q96AY3	FKBP10;FKBP9	FKBP prolyl isomerase 9	-0.71	decreased	1.3E-02	3 / 3
P23284	P23284	PPIB	peptidylprolyl isomerase B	-0.71	decreased	4.7E-03	3 / 3
O00264.1	O00264;O15173	PGRMC1;PGRMC2	progesterone receptor membrane component 1	-0.71	decreased	1.7E-03	3 / 3
Q86SX6	Q86SX6	GLRX5	glutaredoxin 5	-0.72	decreased	4.1E-02	3 / 3
Q70Z53	Q70Z53	FRA10AC1	FRA10A associated CGG repeat 1	-0.73	decreased	3.6E-02	3 / 3
Q9BQ48	Q9BQ48	MRPL34	mitochondrial ribosomal protein L34	-0.73	decreased	2.5E-03	3 / 3
Q9H223.5	Q9H223;Q9NZN4	EHD2;EHD4	EH domain containing 4	-0.73	decreased	2.0E-03	3 / 3
Q6FIF0	Q6FIF0	ZFAND6	zinc finger AN1-type containing 6	-0.73	decreased	2.2E-04	3 / 3
O75449.1	O75449;Q9BW62	KATNA1;KATNAL1	katanin catalytic subunit A1	-0.75	decreased	4.4E-02	3 / 3
Q08170.1	Q08170;Q13243;Q13247	SRSF4;SRSF5;SRSF6	serine and arginine rich splicing factor 4	-0.75	decreased	2.0E-02	3 / 3
O15014	O15014	ZNF609	zinc finger protein 609	-0.75	decreased	4.7E-03	3 / 3
A0A804HIE8	A0A804HIE8	DOCK9	dedicator of cytokinesis 9	-0.76	decreased	1.6E-02	2 / 2
P48382	P48382	RFX5	regulatory factor X5	-0.76	decreased	1.6E-02	3 / 3
Q9NWV4	Q9NWV4	CZIB	CXXC motif containing zinc binding protein	-0.77	decreased	1.6E-02	3 / 3
Q96DA6	Q96DA6	DNAJC19	DnaJ heat shock protein family (Hsp40) member C19	-0.77	decreased	1.1E-02	3 / 3
Q9NYV6	Q9NYV6	RRN3	RRN3 homolog, RNA polymerase I transcription factor	-0.77	decreased	7.5E-03	3 / 3
P51784.2	P51784;Q13107;Q9Y4E8	USP11;USP15;USP4	ubiquitin specific peptidase 11	-0.78	decreased	9.9E-03	3 / 3
Q9NZJ5	Q9NZJ5	EIF2AK3	eukaryotic translation initiation factor 2 alpha kinase 3	-0.79	decreased	4.0E-02	3 / 3
Q8NCJ5	Q8NCJ5	SPRYD3	SPRY domain containing 3	-0.79	decreased	4.6E-02	3 / 3
P50570.1	P50570;Q05193	DNM1;DNM2	dynamin 2	-0.80	decreased	3.9E-02	3 / 3
P12268.1	P12268;P20839	IMPDH1;IMPDH2	inosine monophosphate dehydrogenase 2	-0.80	decreased	2.8E-03	3 / 3
O60814.1	O60814;P57053;P58876;P62807;Q5QNW6;Q93079;Q99877;Q99879;Q99880	H2BC12;H2BC12L;H2BC13; H2BC14;H2BC15;H2BC18; H2BC4;H2BC5;H2BC9	H2B clustered histone 12	-0.81	decreased	4.2E-02	3 / 3
A2VDJ0	A2VDJ0	TMEM131L	transmembrane 131 like	-0.81	decreased	4.9E-02	3 / 2

Identifier	UniProt accessions (protein group)	Gene name(s)	Protein name	log2FC	Direction	q-value	Valid values sham-CM / irradiated-CM (n/3)
Q6ZN54	Q6ZN54	DEF8	differentially expressed in FDCP 8 homolog	-0.81	decreased	4.0E-02	2 / 2
O43676	O43676	NDUFB3	NADH:ubiquinone oxidoreductase subunit B3	-0.82	decreased	8.6E-03	3 / 3
P06732	P06732;P12277	CKB;CKM	creatine kinase, M-type	-0.83	decreased	4.6E-02	2 / 2
Q9P0N9	Q9P0N9	TBC1D7	TBC1 domain family member 7	-0.83	decreased	1.6E-04	3 / 3
O43739.2	O43739;Q15438;Q99418	CYTH1;CYTH2;CYTH3	cytohesin 3	-0.84	decreased	2.0E-02	3 / 3
Q9P289.1	Q9P289;Q9Y6E0	STK24;STK26	serine/threonine kinase 26	-0.84	decreased	1.1E-02	3 / 3
P07900.10	P07900;Q58FG1	HSP90AA1;HSP90AA4P	heat shock protein 90 alpha family class A member 1	-0.84	decreased	1.9E-04	3 / 3
Q9H0G5	Q9H0G5	NSRP1	nuclear speckle splicing regulatory protein 1	-0.85	decreased	5.0E-02	3 / 2
P08581.1	P08581;P12931;P42685	FRK;MET;SRC	MET proto-oncogene, receptor tyrosine kinase	-0.86	decreased	1.5E-02	3 / 3
O60437	O60437	PPL	periplakin	-0.86	decreased	3.6E-02	2 / 2
O94842	O94842	TOX4	TOX high mobility group box family member 4	-0.86	decreased	3.4E-02	3 / 3
O75525.1	O75525;Q07666	KHDRBS1;KHDRBS3	KH RNA binding domain containing, signal transduction associated 3	-0.86	decreased	9.7E-04	3 / 3
P57721.1	P57721;P57723;Q15365;Q1536 6	PCBP1;PCBP2;PCBP3;PC BP 4	poly(rC) binding protein 3	-0.87	decreased	2.6E-03	3 / 3
Q5JU69	Q5JU69	TOR2A	torsin family 2 member A	-0.88	decreased	4.0E-02	3 / 3
Q9NSA3	Q9NSA3	CTNNBIP1	catenin beta interacting protein 1	-0.88	decreased	2.6E-02	3 / 3
Q8N3D4.1	Q8N3D4;Q8NDI1	EHBP1;EHBP1L1	EH domain binding protein 1 like 1	-0.89	decreased	2.7E-04	3 / 3
Q9BT81 †	Q9BT81	SOX7	SRY-box transcription factor 7	-0.90	decreased	2.4E-02	2 / 0
A0A2R8Y5A6	A0A2R8Y5A6;A0A2R8Y6W7;A0 A2R8YDM9;A0A2R8YGV1	ATXN2	ataxin 2	-0.90	decreased	1.2E-02	2 / 1
B1AK88	B1AK88	CAPZB	capping actin protein of muscle Z-line subunit beta	-0.91	decreased	1.6E-05	3 / 3
Q9HC77	Q9HC77	CENPJ	centromere protein J	-0.91	decreased	5.9E-03	3 / 3
Q92839	Q92839	HAS1	hyaluronan synthase 1	-0.93	decreased	2.5E-02	3 / 3
P35080	P35080	PFN2	profilin 2	-0.93	decreased	1.6E-03	3 / 3
Q8TD16.1	Q8TD16;Q96G01	BICD1;BICD2	BICD cargo adaptor 2	-0.93	decreased	2.2E-06	3 / 3
Q8N6T7	Q8N6T7	SIRT6	sirtuin 6	-0.95	decreased	1.9E-06	3 / 3
Q12800.1	Q12800;Q9NZI7	TFCP2;UBP1	transcription factor CP2	-0.96	decreased	5.0E-04	3 / 3

Identifier	UniProt accessions (protein group)	Gene name(s)	Protein name	log2FC	Direction	q-value	Valid values sham-CM / irradiated-CM (n/3)
P20700.1	P20700;Q03252	LMNB1;LMNB2	lamin B1	-0.96	decreased	3.4E-03	3 / 3
Q9NP97	Q9NP97	DYNLRB1	dynein light chain roadblock-type 1	-0.96	decreased	5.1E-03	3 / 3
L0R6Q1	L0R6Q1	SLC35A4	solute carrier family 35 member A4	-0.96	decreased	4.1E-05	3 / 3
F5GZQ4	F5GZQ4	LDHA	lactate dehydrogenase A	-0.97	decreased	2.3E-03	3 / 2
O95402	O95402	MED26	mediator complex subunit 26	-0.97	decreased	3.4E-02	3 / 1
O95319.1	O95319;Q92879	CELF1;CELF2	CUGBP Elav-like family member 2	-0.97	decreased	3.0E-05	3 / 3
Q14657	Q14657	LAGE3	L antigen family member 3	-0.97	decreased	4.9E-03	2 / 3
Q9NRX2	Q9NRX2	MRPL17	mitochondrial ribosomal protein L17	-0.98	decreased	1.5E-02	3 / 3
Q9NV64	Q9NV64	TMEM39A	transmembrane protein 39A	-0.98	decreased	5.3E-03	2 / 2
Q969V6	Q969V6	MRTFA	myocardin related transcription factor A	-0.98	decreased	9.3E-03	3 / 3
Q5SW79.1	Q5SW79;Q9Y4F5	CEP170;CEP170B	centrosomal protein 170	-0.98	decreased	3.5E-04	3 / 3
P46459.1	P46459;Q969G3	NSF;SMARCE1	N-ethylmaleimide sensitive factor, vesicle fusing ATPase	-0.98	decreased	1.7E-02	3 / 3
O14939	O14939	PLD2	phospholipase D2	-0.99	decreased	1.0E-02	3 / 3
P62195.1	P62195;Q8NB90	PSMC5;SPATA5	proteasome 26S subunit, ATPase 5	-1.00	decreased	1.6E-05	3 / 3
Q9H1J1	Q9H1J1	UPF3A	UPF3A regulator of nonsense mediated mRNA decay	-1.01	decreased	6.6E-04	2 / 1
P15153.2	P15153;P60763;P63000	RAC1;RAC2;RAC3	Rac family small GTPase 2	-1.02	decreased	6.7E-04	3 / 3
Q6NW29	Q6NW29	RWDD4	RWD domain containing 4	-1.03	decreased	3.8E-03	3 / 3
P14618.1	P14618;P30613	PKLR;PKM	pyruvate kinase M1/2	-1.03	decreased	6.3E-04	3 / 3
Q9Y689	Q9Y689	ARL5A	ADP ribosylation factor like GTPase 5A	-1.03	decreased	3.8E-02	3 / 2
Q7Z769	Q7Z769	SLC35E3	solute carrier family 35 member E3	-1.04	decreased	2.8E-04	3 / 3
O95613	O95613	PCNT	pericentrin	-1.04	decreased	1.8E-04	3 / 3
Q6STE5.3	Q6STE5;Q96GM5	SMARCD1;SMARCD3	SWI/SNF related, matrix associated, actin dependent regulator of chromatin, subfamily d, member 3	-1.06	decreased	2.2E-05	3 / 3
Q8IXI1.1	Q8IXI1;Q8IXI2	RHOT1;RHOT2	ras homolog family member T2	-1.07	decreased	2.9E-02	3 / 2
P57721.3	P57721;Q15366	PCBP2;PCBP3	poly(rC) binding protein 3	-1.07	decreased	3.7E-05	3 / 3
O14618	O14618	CCS	copper chaperone for superoxide dismutase	-1.08	decreased	2.5E-03	3 / 3
P22681.1	P22681;Q13191	CBL;CBLB	Cbl proto-oncogene	-1.08	decreased	1.0E-02	3 / 3
O43252.1	O43252;O95340	PAPSS1;PAPSS2	3'-phosphoadenosine 5'-phosphosulfate synthase 1	-1.09	decreased	4.1E-02	3 / 3
Q8IZ13	Q8IZ13	ZBED8	family with sequence similarity 200 member C	-1.10	decreased	1.0E-04	1 / 2

Identifier	UniProt accessions (protein group)	Gene name(s)	Protein name	log2FC	Direction	q-value	Valid values sham-CM / irradiated-CM (n/3)
Q9NYA1	Q9NYA1	SPHK1	sphingosine kinase 1	-1.13	decreased	5.3E-03	2 / 2
P02795.1	P02795;P04732;P80297	MT1E;MT1X;MT2A	metallothionein 2A	-1.14	decreased	1.0E-03	3 / 3
Q9NX05.1	Q9NX05;Q9NZB2	FAM120A;FAM120C	family with sequence similarity 120 member C	-1.15	decreased	6.6E-04	3 / 3
P01116	P01116	KRAS	KRAS proto-oncogene, GTPase	-1.15	decreased	2.5E-03	3 / 3
P05362	P05362	ICAM1	intercellular adhesion molecule 1	-1.17	decreased	3.6E-02	2 / 2
Q96IW7	Q96IW7	SEC22A	SEC22 homolog A, vesicle trafficking protein	-1.17	decreased	1.5E-03	3 / 2
Q86TX2	Q86TX2	ACOT1	acyl-CoA thioesterase 1	-1.18	decreased	2.0E-02	3 / 2
Q7Z7F7	Q7Z7F7	MRPL55	mitochondrial ribosomal protein L55	-1.18	decreased	2.6E-03	3 / 3
Q53S58	Q53S58	TMEM177	transmembrane protein 177	-1.18	decreased	1.5E-05	3 / 3
Q8TD22	Q8TD22	SFXN5	sideroflexin 5	-1.22	decreased	2.5E-02	3 / 1
Q53SF7	Q53SF7	COBLL1	cordon-bleu WH2 repeat protein like 1	-1.22	decreased	1.4E-02	3 / 3
Q86X95	Q86X95	CIR1	corepressor interacting with RBPJ, CIR1	-1.25	decreased	9.6E-03	3 / 3
Q9Y605	Q9Y605	MRFAP1	Morf4 family associated protein 1	-1.25	decreased	4.3E-03	3 / 3
Q16512.1	Q16512;Q16513;Q6P5Z2	PKN1;PKN2;PKN3	protein kinase N1	-1.26	decreased	2.7E-02	3 / 3
P61165	P61165	TMEM258	transmembrane protein 258	-1.27	decreased	1.1E-04	3 / 3
P20062 †	P20062	TCN2	transcobalamin 2	-1.28	decreased	4.5E-03	2 / 0
Q9UHQ1	Q9UHQ1	NARF	nuclear prelamin A recognition factor	-1.30	decreased	7.3E-03	3 / 3
O43513	O43513	MED7	mediator complex subunit 7	-1.31	decreased	4.4E-03	3 / 2
Q5JPE7	Q5JPE7	NOMO2	NODAL modulator 2	-1.31	decreased	5.2E-03	3 / 2
Q9BQ24 †	Q9BQ24	ZFYVE21	zinc finger FYVE-type containing 21	-1.32	decreased	4.0E-10	3 / 0
O60885.2	O60885;P25440;Q15059	BRD2;BRD3;BRD4	bromodomain containing 4	-1.33	decreased	2.5E-04	3 / 3
Q9UI32	Q9UI32	GLS2	glutaminase 2	-1.36	decreased	8.3E-04	3 / 2
P29558.1	P29558;Q15434	RBMS1;RBMS2	RNA binding motif single stranded interacting protein 1	-1.37	decreased	1.7E-02	3 / 2
P31946.3	P31946;P63104	YWHAB;YWHAZ	tyrosine 3-monooxygenase/tryptophan 5-monooxygenase activation protein beta	-1.41	decreased	9.1E-04	3 / 2
P63167	P63167	DYNLL1	dynein light chain LC8-type 1	-1.44	decreased	9.1E-03	3 / 3
O15243 †	O15243	LEPROT	leptin receptor overlapping transcript	-1.47	decreased	1.0E-02	0 / 2
P52333	P52333	JAK3	Janus kinase 3	-1.47	decreased	1.1E-10	3 / 3
P47974	P47974	ZFP36L2	ZFP36 ring finger protein like 2	-1.49	decreased	4.9E-02	3 / 3
P46940.1	P46940;Q13576	IQGAP1;IQGAP2	IQ motif containing GTPase activating protein 1	-1.50	decreased	6.1E-05	3 / 3
P30405.1	P30405;Q9UNP9	PPIE;PPIF	peptidylprolyl isomerase F	-1.53	decreased	2.4E-06	3 / 3

Identifier	UniProt accessions (protein group)	Gene name(s)	Protein name	log2FC	Direction	q-value	Valid values sham-CM / irradiated-CM (n/3)
P33991.1	P33991;Q9UJA3	MCM4;MCM8	minichromosome maintenance complex component 4	-1.54	decreased	3.1E-02	3 / 3
Q15365.1	Q15365;Q15366	PCBP1;PCBP2	poly(rC) binding protein 1	-1.55	decreased	2.8E-10	3 / 3
P13639.1	P13639;Q15029	EEF2;EFTUD2	eukaryotic translation elongation factor 2	-1.56	decreased	7.1E-07	3 / 3
P01112	P01112	HRAS	HRas proto-oncogene, GTPase	-1.56	decreased	2.6E-04	3 / 2
Q5W0V3.1	Q5W0V3;Q86V87	FHIP2A;FHIP2B	FHF complex subunit HOOK interacting protein 2A	-1.57	decreased	4.3E-09	3 / 1
O43739.3	O43739;Q99418	CYTH2;CYTH3	cytohesin 3	-1.58	decreased	7.5E-03	3 / 1
Q96BP2	Q96BP2	CHCHD1	coiled-coil-helix-coiled-coil-helix domain containing 1	-1.64	decreased	4.5E-02	2 / 2
Q96LI5.1	Q96LI5;Q9ULM6	CNOT6;CNOT6L	CCR4-NOT transcription complex subunit 6 like	-1.66	decreased	6.3E-05	3 / 3
Q15532	Q15532	SS18	SS18 subunit of BAF chromatin remodeling complex	-1.69	decreased	1.1E-03	3 / 3
P00338.1	P00338;P07195	LDHA;LDHB	lactate dehydrogenase A	-1.69	decreased	8.1E-10	3 / 3
Q9NUU7	Q9NUU7	DDX19A	DEAD-box helicase 19A	-1.70	decreased	5.6E-04	3 / 3
Q86TP1	Q86TP1	PRUNE1	prune exopolyphosphatase 1	-1.72	decreased	1.1E-02	3 / 2
P04908.2 †	P04908;Q7L7L0;Q93077	H2AC4;H2AC6;H2AW	H2A clustered histone 4	-1.75	decreased	4.7E-02	2 / 0
Q9Y289 †	Q9Y289	SLC5A6	solute carrier family 5 member 6	-1.78	decreased	1.2E-03	2 / 0
P01111.3	P01111;P01116	KRAS;NRAS	NRAS proto-oncogene, GTPase	-1.78	decreased	9.3E-04	3 / 3
Q9BSB4	Q9BSB4	ATG101	autophagy related 101	-1.79	decreased	3.3E-02	3 / 3
Q9H223.2	Q9H223;Q9H4M9;Q9NZN4	EHD1;EHD2;EHD4	EH domain containing 4	-1.82	decreased	1.1E-04	3 / 3
P30047	P30047	GCHFR	GTP cyclohydrolase I feedback regulator	-1.83	decreased	2.5E-02	2 / 2
P42685	P42685	FRK	fyn related Src family tyrosine kinase	-1.85	decreased	7.9E-04	3 / 3
P07437.1	P07437;P68371;Q13509	TUBB;TUBB3;TUBB4B	tubulin beta class I	-1.87	decreased	3.7E-03	3 / 3
Q96HN2	Q96HN2	AHCYL2	adenosylhomocysteinase like 2	-1.88	decreased	6.2E-10	3 / 3
D6RBR0	D6RBR0	G3BP2	G3BP stress granule assembly factor 2	-1.88	decreased	4.6E-02	2 / 1
Q5W0Z9	Q5W0Z9	ZDHHC20	zinc finger DHHC-type palmitoyltransferase 20	-1.93	decreased	1.7E-02	3 / 2
Q9H7B4	Q9H7B4	SMYD3	SET and MYND domain containing 3	-1.96	decreased	3.8E-02	3 / 2
P43360	P43360	MAGEA6	MAGE family member A6	-1.97	decreased	3.2E-03	3 / 1
O43739.1	O43739;Q15438	CYTH1;CYTH3	cytohesin 3	-1.98	decreased	3.0E-05	3 / 2
Q5UCC4	Q5UCC4	EMC10	ER membrane protein complex subunit 10	-2.00	decreased	2.3E-08	3 / 2
Q32P28.1 †	Q32P28;Q8IVL5	P3H1;P3H2	prolyl 3-hydroxylase 1	-2.00	decreased	4.8E-04	3 / 0
E9PHC9	E9PHC9	KLF7	KLF transcription factor 7	-2.02	decreased	1.1E-06	3 / 1

Identifier	UniProt accessions (protein group)	Gene name(s)	Protein name	log2FC	Direction	q-value	Valid values sham-CM / irradiated-CM (n/3)
A0A087WUT0	A0A087WUT0;A0A0A0MQW0;A0A0A0MR39;A0A0C4DGV1;H0Y N19	MYEF2	myelin expression factor 2	-2.04	decreased	2.2E-03	3 / 3
Q8NDV7.1	Q8NDV7;Q9HCJ0	TNRC6A;TNRC6C	trinucleotide repeat containing adaptor 6A	-2.09	decreased	7.9E-03	2 / 1
P0CG12	P0CG12	DERPC	DERPC proline and glycine rich nuclear protein	-2.16	decreased	2.4E-04	3 / 1
E9PK54 †	E9PK54	HSPA8	heat shock protein family A (Hsp70) member 8	-2.21	decreased	3.1E-02	2 / 0
Q8NDT2.1	Q8NDT2;Q96T37	RBM15;RBM15B	RNA binding motif protein 15B	-2.23	decreased	8.0E-05	2 / 1
P0CG38.1	P0CG38;P60709;P63261	ACTB;ACTG1;POTEI	POTE ankyrin domain family member I	-2.24	decreased	6.9E-04	3 / 3
Q68CZ2.1	Q68CZ2;Q9HBL0	TNS1;TNS3	tensin 3	-2.25	decreased	2.3E-06	3 / 1
Q5BKY9	Q5BKY9;Q8N9E0	FAM133A;FAM133B	family with sequence similarity 133 member B	-2.27	decreased	2.4E-13	3 / 1
C9JTX5 †	C9JTX5	ACTB	actin beta	-2.29	decreased	3.8E-13	2 / 0
P07477	P07477;P07478;Q8NHM4	PRSS1;PRSS2;PRSS3P2	serine protease 1	-2.33	decreased	1.2E-07	3 / 3
P24723.1	P24723;Q02156	PRKCE;PRKCH	protein kinase C eta	-2.35	decreased	3.0E-02	3 / 2
P23246.1	P23246;Q15233	NONO;SFPQ	splicing factor proline and glutamine rich	-2.39	decreased	1.5E-02	3 / 2
P46531.1	P46531;Q04721	NOTCH1;NOTCH2	notch receptor 1	-2.41	decreased	3.7E-11	3 / 3
A0A494C1R9	A0A494C1R9;A6NKD2;P0CV98;P0CV99;P0CW00;P0CW01;Q01 534	TSPY1;TSPY10;TSPY2;TSPY3;TSPY4;TSPY8;TSPY9	testis specific protein Y-linked 1	-2.41	decreased	5.9E-03	3 / 2
Q9Y5W9	Q9Y5W9	SNX11	sorting nexin 11	-2.44	decreased	5.1E-04	3 / 3
A4FU01	A4FU01	MTMR11	myotubularin related protein 11	-2.48	decreased	9.5E-04	3 / 2
O14802.1	O14802;O95602	POLR1A;POLR3A	RNA polymerase III subunit A	-2.49	decreased	2.4E-06	3 / 2
H3BSM3	H3BSM3	POLR3E	RNA polymerase III subunit E	-2.51	decreased	7.5E-03	3 / 2
F8VXC8	F8VXC8	SMARCC2	SWI/SNF related, matrix associated, actin dependent regulator of chromatin subfamily c member 2	-2.56	decreased	9.5E-11	3 / 1
Q2M1P5.1 †	Q2M1P5;Q7Z4S6	KIF21A;KIF7	kinesin family member 7	-2.60	decreased	2.4E-13	3 / 0
A5A3E0.4	A5A3E0;P60709;P63261;Q562R 1	ACTB;ACTBL2;ACTG1;POTE F	POTE ankyrin domain family member F	-2.95	decreased	6.1E-04	3 / 2
P00338.3	P00338;Q6ZMR3	LDHA;LDHAL6A	lactate dehydrogenase A	-3.05	decreased	5.3E-04	3 / 3
O00462.1 †	O00462;Q9NQW7	MANBA;XPNPEP1	mannosidase beta	-3.26	decreased	3.6E-03	2 / 0
Q8TF01	Q8TF01	PNISR	PNN interacting serine and arginine rich protein	-3.31	decreased	3.3E-07	3 / 1

Identifier	UniProt accessions (protein group)	Gene name(s)	Protein name	log2FC	Direction	q-value	Valid values sham-CM / irradiated-CM (n/3)

log2FC = log2(mean abundance in irradiated-CM / mean abundance in sham-CM); positive values indicate higher abundance in bystander cells exposed to irradiated-CM.

q-value: DEP-adjusted p-value (fdrtool q-value) from the limma moderated t-test.

Identifier: leading UniProt accession of the protein group, as used in the text. Numerical suffixes (.1, .2, …) were added by the DEP software to distinguish protein groups sharing the same leading accession; they are not UniProt isoforms. The full protein group is given in the next column.
Protein name: name of the gene of the leading accession; when a protein group contains several genes, the peptides used for quantification are shared between these proteins.

† Detected in only one condition (≥ 2/3 replicates in one group, 0/3 in the other): the fold change depends on imputed values and should be read as presence/absence.

Table S3. Gene Ontology (GO) term enrichment for "bystander" chondrocytes T/C28-A2, which received medium from SW1353 chondrosarcoma cells irradiated with protons at a dose of 0.1 Gy.

GO ID	GO term	Associated Genes (%)	Nr. Genes	Gene associate
GO:0042733	embryonic digit morphogenesis	5.00	3.00	B9D1, LRP5, TWIST1
GO:0045668	negative regulation of osteoblast dif-ferentiation	6.25	3.00	HDAC4, LRP5, TWIST1
GO:0098918	structural constituent of synapse	12.50	3.00	ACTB, ACTN1, MPP2
GO:0099186	structural constituent of postsynapse	16.67	3.00	ACTB, ACTN1, MPP2

Table S4. Gene Ontology (GO) term enrichment for "bystander" endothelial cells EA.hy926, which received medium from SW1353 chondrosarcoma cells irradiated with protons at a dose of 2 Gy.

GO ID	GO term	Associated Genes (%)	Nr. Genes	Gene associate
GO:0000082	G1/S transition of mitotic cell cycle	6.84	16.00	ACTB, AKT1, APC, BRD4, BRIP1, CDC34, CENPJ, MDM2, PLRG1, POTEF, SMARCA2, SMARCB1, SMARCC2, SMARCD3, TRIAP1, WEE1
GO:0005200	structural constit-uent of cytoskele-ton	9.82	11.00	ACTB, EPB41, LMNB1, MSN, POTEF, PPL, TUBA1C, TUBA3C, TUBB, TUBB4A, YEATS4
GO:2000045	regulation of G1/S transition of mitotic cell cycle	7.74	13.00	ACTB, AKT1, APC, CENPJ, MDM2, PLRG1, POTEF, SMARCA2, SMARCB1, SMARCC2, SMARCD3, TRIAP1, WEE1
GO:0005338	nucleotide-sugar transmembrane transporter activ-ity	33.33	4.00	SLC35A3, SLC35A4, SLC35B1, SLC35E3

GO:2000819	regulation of nu-cleotide-excision repair	20.69	6.00	ACTB, POTEF, SMARCA2, SMARCB1, SMARCC2, SMARCD3
GO:0007010	cytoskeleton or-ganization	4.36	70.00	ACTB, APC, AQP1, ARHGEF10, AURKA, BICD2, CAPZA2, CAPZB, CCNB2, CCNL1, CENPJ, CHMP2A, CHMP5, CLASP2, DNAAF2,
**GO ID**	**GO term**	**Associated Genes (%)**	**Nr. Genes**	**Gene associate**
				DYNLL1, EHBP1, EHBP1L1, EPB41, HDAC3, HRAS, IQCJ-SCHIP1, IQGAP1, KANK1, KATNA1, KRAS, LMNB1, MAST3, MET, MI-CALL2, MRTFA, MSN, MYH7B, MYH9, MYL9, MYO1B, MYO1C, MYO1D, NRAS, PCNT, PFN2, PFN3, PHLDB1, PKN1, PLD2, PLEKHG2, POTEF, PPL, PRUNE1, RAC2, RAF1, SLC9A1, SPRYD3, SS18, SSH1, STK19, SYDE1, TAOK1, TNFAIP1, TNKS, TOX4, TRAF3, TRAF3IP1, TTC26, TUBA1C, TUBA3C, TUBB, TUBB4A, WEE1, YEATS4
GO:0015931	nucleobase-con-taining compound transport	6.53	16.00	ATXN2, BICD2, DDX19A, G3BP2, IGF2BP3, LRRC8E, MYO1C, NCBP1, RBM15B, SLC35A3, SLC35A4, SLC35B1, SLC35E3, SRSF1, TNKS, UPF3A
GO:0016071	mRNA metabolic process	4.50	34.00	AGO3, AKT1, CELF2, CIR1, CNOT6L, CSTF2, DHX16, HSPA8, IGF2BP3, KHDRBS3, KIN, LSM7, MBD4, MYEF2, NCBP1, NSRP1, NUDT16, PCBP3, PLRG1, RBM15B, RBM4B, RC3H1, RNPC3, RPUSD4, SFPQ, SRSF1, SRSF4, TBP, TFCP2, TNRC6A, TSEN15, UPF3A, VIRMA, ZFP36L2

GO ID	GO term	Associated Genes (%)	Nr. Gene s	Gene associate
GO:0030036	actin cytoskeleton organization	4.79	37.00	ACTB, AQP1, ARHGEF10, CAPZA2, CAPZB, CLASP2, DYNLL1, EHBP1, EHBP1L1, EPB41, HRAS, IQGAP1, KANK1, KRAS, MET, MI-CALL2, MRTFA, MYH7B, MYH9, MYL9, MYO1B, MYO1C, MYO1D, NRAS, PFN2, PFN3, PKN1, PLEKHG2, POTEF, RAC2, SLC9A1, SSH1, SYDE1, TAOK1, TNFAIP1, TOX4, TRAF3
GO:0030705	cytoskeleton-de-pendent intracel-lular transport	7.31	16.00	BICD2, DYNLL1, HSPA8, KIF13A, MYO1B, MYO1C, MYO1D, RHOT2, SFPQ, STK11, TERF2, TRAF3IP1, TTC26, TTC30A, TUBA1C, TUBB
GO:0032388	positive regula-tion of intracellu-lar transport	6.88	15.00	ANP32B, HDAC3, HRAS, HSP90AA1, LEPROT, MAPK8, MICALL2, MSN, MYL1, MYO1C, PCNT, PRNP, RAC2, SIRT6, STK11
GO:0033043	regulation of or-ganelle organiza-tion	4.28	55.00	ACTB, AKT1, APC, ARHGEF10, ARMH3, AURKA, BICD2, CAPZA2, CAPZB, CENPJ, CHMP2A, CHMP5, CLASP2, CNOT6L, DNM2, DYNLL1, G3BP2, HRAS, IQCJ-SCHIP1,
				IQGAP1, KANK1, MAPK8, MET, MSN, OMA1, PFN2, PFN3, PHLDB1, PKN1, PLEKHG2, PO-TEF, PPIF, PRUNE1, RAC2, RHOT2, SFPQ, SIRT6, SLC9A1, SMARCA2, SMARCB1, SMARCC2, SMARCD3, SPHK1, SSH1, STK19, SYDE1, TAOK1, TERF2, TMEM33, TMEM39A, TNKS, TOX4, TRAF3IP1, TRIAP1, TUBB4A

GO:0044087	regulation of cel-lular component biogenesis	4.61	49.00	APC, ARHGEF10, CAPZA2, CAPZB, CDC34, CENPJ, CHMP2A, CHMP5, CLASP2, CNOT6L, CTNNBIP1, DEF8, DNM2, DYNLL1, EPHB3, FLRT2, G3BP2, HRAS, HSP90AA1, HSPA8, ICAM1, KANK1, MET, MSN, MYO1C, NLRC3, NOTCH1, NUDT16, PFN2, PFN3, PHLDB1, PKN1, PLEKHG2, PPP2R5B, PRKCH, PRUNE1, PSMC5, RAC2, RAF1, SLC9A1, SPHK1, SSH1, STK19, TERF2, THBS1, TMEM39A, TOX4, TRAF3IP1, TUBB4A
GO:0046578	regulation of Ras protein signal transduction	6.79	15.00	ARHGEF10, CBL, CYTH3, DNM2, DYNLL1, KANK1, KRAS, MET, NOTCH1, NRAS, RAF1, RASA2, STK19, SYDE1, TNFAIP1
GO:0046907	intracellular transport	4.14	69.00	AKT1, ALKBH7, ANP32B, AP4B1, APPBP2, ARL5A, BICD2, CHMP2A, CHMP5, DDX19A, DNAJC19, DOP1B, DYNLL1, EHD4, GRAMD1A, HDAC3, HRAS, HSP90AA1, HSPA8, ICMT, KDELR1, KDELR3, KIF13A, LEP-ROT, LMNB1, MAPK8, MBD4, MICALL2, MON1A, MSN, MYL1, MYO1B, MYO1C, MYO1D, NCBP1, NOTCH1, NSF, NUS1, PCNT, PKIG, PLEKHA8, PPIF, PRNP, RAC2, RBM15B, RHOT2, SEC22A, SEC61A1, SFPQ, SIRT6, SLC25A14, SLC9A1, SNX11, STK11, TAP2, TERF2, TOX4, TRAF3IP1, TRAM1, TTC26, TTC30A, TUBA1C, TUBB, UPF3A, YIPF6, YWHAB, YWHAQ, ZDHHC20, ZFAND6
GO:0051493	regulation of cy-toskeleton organi-zation	4.89	28.00	APC, ARHGEF10, BICD2, CAPZA2, CAPZB, CENPJ, CHMP2A, CHMP5, CLASP2, DYNLL1, HRAS, IQCJ-SCHIP1, IQGAP1, KANK1, MET, PFN2, PFN3, PHLDB1, PKN1, PLEKHG2, PRUNE1, RAC2, SLC9A1, SSH1, SYDE1, TAOK1, TRAF3IP1, TUBB4A]
**GO ID**	**GO term**	**Associated Genes (%)**	**Nr. Gene**	**Gene associate**

			s	
GO:0070507	regulation of mi-crotubule cyto-skeleton organiza-tion	7.69	13.00	APC, BICD2, CENPJ, CHMP2A, CHMP5, CLASP2, MET, PHLDB1, PKN1, PRUNE1, TAOK1, TRAF3IP1, TUBB4A
GO:0070897	transcription preinitiation com-plex assembly	10.98	9.00	BRF1, MBD4, MED19, MED26, MED7, RRN3, SMARCB1, TBP, ZFP36L2
GO:0071824	protein-DNA com-plex subunit or-ganization	6.35	19.00	ANP32B, ASF1B, BRF1, H2BC12, KALRN, MBD4, MED19, MED26, MED7, RRN3, SIRT6, SMARCB1, SMARCC2, SMARCD3, SMYD3, TBP, TSPY9, YEATS4, ZFP36L2
GO:0045023	G0 to G1 transi-tion	17.02	8.00	ACTB, MBD4, POTEF, PPP2R5B, SMARCA2, SMARCB1, SMARCC2, SMARCD3
GO:0070316	regulation of G0 to G1 transition	17.78	8.00	ACTB, MBD4, POTEF, PPP2R5B, SMARCA2, SMARCB1, SMARCC2, SMARCD3

Table S5. STRING Network enrichment and associated proteins for EA.hy926 after receiving medium from SW1353 exposed to 2 Gy

term ID	term description	strength	false discovery rate	matching proteins in your network (labels)
GO:0044237	Cellular metabolic process	0.16	0.0031	RHBDD2,APPBP2,PPP2R5B,RRN3,CDC34,TCN2,AURKA,CDIPT,CKM,HAS1,CHMP5, TNFAIP1,KALRN,ALKBH7,NSRP1,MBD4,HBA2,TRPC4AP,MRPL34,LSM7,TERF2,APC,PNPLA8,MDM2,BRIP1,GCHFR,MIB1,MAST3,PLD2,ITPKC,MED26,POLQ,ALG10,PNKD, RNF7,ZFP36L2,MED7,MRPL17,CCNL1,VIRMA,RPUSD4,POLR3E,LDHAL6B,KLF13,PGK2, EIF2AK3,UTP23,RCE1,GLS2,ACOT1,TNKS,SPHK1,SSH1,AHCYL2,COX5A,CAMK2G,LY-PLA1,STK11,TMEM39A,RNF166,GLRX5,UQCR10,SIRT6,ATG101,CIR1,UBE3B,SMARCB1,STAT5A,USP9Y,NBR1,ICMT,DMPK,LAGE3,MAP3K6,MT-CO1,RNF121,MAP3K3,MT-CO3,TSEN15,GNPAT,SDHC,NUS1,ATP5PB,ATP5MD, SLC35A3,GCLM,PLPP3,FRA10AC1, ERI3,DOLPP1,CHCHD1,PKIG,UGCG,CSGALNACT2,NCBP1,UPF3A,STK19,NMNAT1, KIN,APTX,MRPL2,TSN,GALNT3,ATN1,PRPS2,ZDHHC20,TONSL,MTMR11,PPIP5K1,WEE1,ZDHHC17,MRPL55,EZH1,NDUFB3,HRAS,CBFB,RPL7L1,BTBD9,SMYD3,PLRG1,PRPS1L1,INTS8,PTDSS1,JAK3,RNPC3,RBM4B,CCS,HSPA8,GRAMD1A,TMEM258,LDHA,ATXN2,BRF1,SMARCC2,PTGR2,POP4,UQCR11,CHMP2A,SETD1B,NUDT3,FRK,SLC25A14,IRAK4,EYA1,MED19
GO:0008152	Metabolic process	0.12	0.0268	RHBDD2,APPBP2,PPP2R5B,RRN3,CDC34,TCN2,MYH9,AURKA,CDIPT,CKM,HAS1, CHMP5,TNFAIP1,KALRN,ALKBH7,NSRP1,YEATS4,MBD4,HBA2,TRPC4AP,MRPL34,LSM7,TERF2,APC,PNPLA8,MDM2,BRIP1,GCHFR,MIB1,MAST3,PLD2,ITPKC,MED26,POLQ, ALG10,PNKD,RNF7,ZFP36L2,MED7,MRPL17,CCNL1,VIRMA,RPUSD4,POLR3E,PPIB,LDHAL6B,KLF13,PGK2,DDX19A,EIF2AK3,UTP23,PRSS1,RCE1,GLS2,ACOT1,TNKS,SPHK1,SSH1,AHCYL2,COX5A,CAMK2G,LYPLA1,STK11,TMEM39A,RNF166,GLRX5,SPPL2C, UQCR10,SIRT6,ATG101,CIR1,UBE3B,SMARCB1,STAT5A,USP9Y,KLHL7,NBR1,ICMT,DMP

				K,LAGE3,MAP3K6,MT-CO1,RNF121,MAP3K3,MT-CO3,TSEN15,GNPAT,SDHC,NUS1,ATP5PB,ATP5MD,SLC35A3,GCLM,OMA1,PLPP3,FRA10AC1,ERI3,DOLPP1,CHCHD1,PKIG,UGCG,CSGALNACT2,NCBP1,UPF3A,STK19,NMNAT1,KIN,APTX,MRPL2,TSN,GALNT3,TRAF3,ATN1,PRPS2,ZDHHC20,TONSL,MTMR11,BRMS1,PPIP5K1,HECTD2,WEE1,ZDHHC17,MRPL55,EZH1,NDUFB3,HRAS,CBFB,RPL7L1,BTBD9,SMYD3,LMF2,PLRG1,PRPS1L1,INTS8,PTDSS1,JAK3,RNPC3,RBM4B,TSKU,CCS,HSPA8,PHC1,GRAMD1A,RSPRY1,TMEM258,LDHA,ATXN2,BRF1,DTX3,SMARCC2,PTGR2,POP4,UQCR11,CHMP2A,SETD1B,NUDT3,FRK,SLC25A14,IRAK4,PCGF2,ACTB,EYA1,MED19
GO:0005622	Intracellular anatomical structure	0.08	6.58E-10	RHBDD2,APPBP2,PPP2R5B,RRN3,CDC34,TCN2,MYH9,ZFYVE21,AURKA,RP2,CDIPT,CKM,HAS1,CHMP5,TNFAIP1,GNB3,TUSC2,PFN2,KALRN,SEC61A1,ALKBH7,NSRP1,YEATS4,MBD4,HBA2,TRPC4AP,MRPL34,LSM7,TERF2,KRAS,LAMTOR5,APC,PNPLA8,MDM2,BRIP1,KIF13A,GCHFR,MIB1,TRAM1,PLD2,ITPKC,ASF1B,MED26,PNO1,SLC9A1,EHBP1,TUBB4A,POLQ,ALG10,PRUNE1,SFXN5,PNKD,RNF7,SIGMAR1,MYL9,ZFP36L2,ITGA1,MED7,RASA2,MTERF3,CCNB2,MRPL17,MITD1,RFX5,ARL5A,CCNL1,MON1A,MICALL2,VIRMA,DNAAF2,ZNF22,RPUSD4,POLR3E,PPIB,SPRYD3,SOX7,LDHAL6B,KLF13,DERPC,PGK2,DDX19A,EIF2AK3,MYL1,UTP23,RCE1,LRRC7,KLF7,NARF,GLS2,SEC22A,AQP1,ACOT1,TNKS,CC2D1A,SPHK1,CD46,SSH1,AHCYL2,ZNF609,MYEF2,COX5A,MRFAP1,CAMK2G,DEF6,LYPLA1,MYO1D,STK11,TUBA3D,TMEM39A,RNF166,ZBTB11,SMDT1,CNGA2,GLRX5,KDELR1,SPPL2C,FLRT2,UQCR10,EMC10,SIRT6,ATG101,CIR1,PPL,SMARCB1,STAT5A,USP9Y,KLHL7,NBR1,ICMT,ANP32B,DMPK,TTC30A,OSCP1,DYNLRB1,LAGE3,PFN3,NLRC3,PCNT,G3BP2,PHLDB1,MT-CO1,RNF121,MAP3K3,ZNF318,MT-CO3,TSEN15,GNPAT,SDHC,NUS1,AP4B1,ATP5PB,ATP5MD,ARMH3,SLC35A3,GCLM,OMA1,PLPP3,FRA10AC1,DDX31,DOLPP1,CHCHD1,PKIG,TOR2A,TRAF3IP1,UGCG,CSGALNACT2,SLC39A7,TAP2,NCBP1,UPF3A,STK19,NMNAT1,CTNNBIP1,KIN,APTX,CENPJ,DNAJC19,MRPL2,TSN,DYNLL1,GALNT3,TRAF3,SNX11,GPS2,ATN1,SLC35E3,PRPS2,DOP1B,ZDHHC20,CBX6,ZBED8,TONSL,ZC3H6,TMEM131L,MTMR11,PLEKHG2,BRMS1,URGC

				P,PLEKHA8,PRNP,PPIP5K1,ZNF780A,CAPZB,HECTD2,WEE1,TMEM177,ZDHHC17,MRPL55,EZH1,NDUFB3,HRAS,SS18,CBFB,ZC3H18,YIPF6,VWA3B,RPL7L1,BTBD9,ZBTB20,SMYD3,TTC26,IQCJ-SCHIP1,PRRC1, TMEM33,LMF2,PLRG1,SLC35A4,PRPS1L1,FAM92A, INTS8,PTDSS1,JAK3,RNPC3,RBM4B,CCS,HSPA8,PHC1,GRAMD1A,MARVELD1,RSPRY1, TMEM258,TUBA1C,LDHA,ZNF362,ATXN2,BRF1,DTX3,SMARCC2,TRIAP1,PTGR2,TGIF2LX,ARPP19,MYL12A,POP4,UQCR11,CHMP2A,SETD1B,TBC1D7,DNAH11,NUDT3,FRK,NOMO2,TOX4,SLC25A14,ZFAND6,IRAK4,MAGEA6,PCGF2,LEPROT,ACTB,EYA1,PNISR, MYO1C,SLC35B1,MRTFA,MED19,NIF3L1,TSPY9P,CNK3/IPCEF1
GO:0043229	Intracellular organelle	0.1	7.01E-09	RHBDD2,APPBP2,PPP2R5B,RRN3,CDC34,TCN2,MYH9,ZFYVE21,AURKA,RP2,CDIPT, HAS1,CHMP5,TNFAIP1,TUSC2,PFN2,KALRN,SEC61A1,ALKBH7,NSRP1,YEATS4,MBD4, HBA2,MRPL34,LSM7,TERF2,KRAS,LAMTOR5,APC,PNPLA8,MDM2,BRIP1,KIF13A,GCHFR,MIB1,TRAM1,PLD2,ITPKC,ASF1B,MED26,PNO1,SLC9A1,EHBP1,TUBB4A,POLQ, ALG10,PRUNE1,SFXN5,PNKD,RNF7,SIGMAR1,MYL9,ZFP36L2,ITGA1,MED7,MTERF3, CCNB2,MRPL17,MITD1,RFX5,ARL5A,CCNL1,MON1A,MICALL2,VIRMA,DNAAF2, ZNF22,RPUSD4,POLR3E,PPIB,SOX7,LDHAL6B,KLF13,DERPC,PGK2,DDX19A,EIF2AK3,MYL1,UTP23,RCE1,LRRC7,KLF7,NARF,GLS2,SEC22A,AQP1,TNKS,CC2D1A,SPHK1,CD46,SSH1,AHCYL2,ZNF609,MYEF2,COX5A,MRFAP1,CAMK2G,DEF6,LYPLA1,MYO1D, STK11,TUBA3D,TMEM39A,ZBTB11,SMDT1,CNGA2,GLRX5,KDELR1,SPPL2C,FLRT2,UQCR10,EMC10,SIRT6,ATG101,CIR1,PPL,SMARCB1,STAT5A,USP9Y,KLHL7,NBR1,ICMT,ANP32B,DMPK,TTC30A,DYNLRB1,LAGE3,PFN3,NLRC3,PCNT,G3BP2,PHLDB1,MT-CO1,RNF121,ZNF318,MT-CO3,TSEN15,GNPAT,SDHC,NUS1,AP4B1,ATP5PB,ATP5MD, ARMH3,SLC35A3,OMA1,PLPP3,FRA10AC1,DDX31,DOLPP1,CHCHD1,PKIG,TOR2A,TRAF3IP1,UGCG,CSGALNACT2,SLC39A7,TAP2,NCBP1,UPF3A,STK19,NMNAT1,CTNNBIP1, KIN,APTX,CENPJ,DNAJC19,MRPL2,TSN,DYNLL1,GALNT3,TRAF3,SNX11,GPS2,ATN1,SLC35E3,DOP1B,ZDHHC20,CBX6,ZBED8,TONSL,ZC3H6,TMEM131L,BRMS1,URGCP,PLEKHA8,PRNP,ZNF780A,CAPZB,WEE1,TMEM177,ZDHHC17,MRPL55,EZH1,NDUFB3,HRA

				S,SS18,CBFB,ZC3H18,YIPF6,VWA3B,RPL7L1,ZBTB20,SMYD3,TTC26,PRRC1,TMEM33,LMF2,PLRG1,SLC35A4,FAM92A,INTS8,PTDSS1,JAK3,RNPC3,RBM4B,CCS,HSPA8,PHC1,GRAMD1A,MARVELD1,TMEM258,TUBA1C,LDHA,ZNF362,ATXN2,BRF1,DTX3,SMARCC2, TRIAP1,TGIF2LX,ARPP19,MYL12A,POP4,UQCR11,CHMP2A,SETD1B,TBC1D7,DNAH11, NUDT3,FRK,NOMO2,TOX4,SLC25A14,IRAK4,MAGEA6,PCGF2,LEPROT,ACTB,EYA1, PNISR,MYO1C,SLC35B1,MRTFA,MED19,NIF3L1,TSPY9P
GO:0043227	Membrane-bounded organelle	0.1	7.34E-09	RHBDD2,APPBP2,PPP2R5B,RRN3,CDC34,TCN2,MYH9,ZFYVE21,AURKA,RP2,CDIPT, HAS1,CHMP5,TNFAIP1,GNB3,TUSC2,PFN2,KALRN,SEC61A1,ALKBH7,NSRP1,YEATS4, MBD4,HBA2,MRPL34,LSM7,TERF2,KRAS,LAMTOR5,APC,PNPLA8,MDM2,BRIP1,KIF13A,GCHFR,MIB1,TRAM1,PLD2,ITPKC,ASF1B,MED26,PNO1,SLC9A1,EHBP1,TUBB4A, POLQ,ICAM1,ALG10,PRUNE1,SFXN5,PNKD,RNF7,SIGMAR1,ZFP36L2,ITGA1, MED7, MTERF3,CCNB2,MRPL17,MITD1,RFX5,ARL5A,CCNL1,MON1A,MICALL2,VIRMA, ZNF22,RPUSD4,POLR3E,PPIB,SOX7,LDHAL6B,KLF13,DERPC,PGK2,DDX19A,EIF2AK3,UTP23,RCE1,LRRC7,KLF7,NARF,SLC5A6,GLS2,SEC22A,AQP1,TNKS,CC2D1A,SPHK1,CD4, AHCYL2,ZNF609,MYEF2,COX5A,MRFAP1,CAMK2G,DEF6,LYPLA1,MYO1D,STK11, TUBA3D,TMEM39A,ZBTB11,SMDT1,CNGA2,GLRX5,KDELR1,SPPL2C,FLRT2,UQCR10,EMC10,SIRT6,ATG101,CIR1,PPL,SMARCB1,STAT5A,USP9Y,KLHL7,NBR1,ICMT,ANP32B,DMPK,TTC30A,DYNLRB1,LAGE3,PFN3,NLRC3,PHLDB1,MT-CO1,RNF121,ZNF318,MT-CO3,TSEN15,GNPAT,SDHC,NUS1,AP4B1,ATP5PB,ATP5MD, ARMH3,SLC35A3,OMA1, PLPP3,FRA10AC1,DDX31,DOLPP1,CHCHD1,PKIG,TOR2A,TRAF3IP1,UGCG,CSGAL-NACT2,SLC39A7,TAP2,NCBP1,UPF3A,STK19,NMNAT1,CTNNBIP1,KIN,APTX,CENPJ,DNAJC19,MRPL2,TSN,DYNLL1,GALNT3,TRAF3,SNX11,GPS2,ATN1,SLC35E3,DOP1B,ZDHHC20,CBX6,ZBED8,TONSL,ZC3H6,TMEM131L,MTMR11,BRMS1,URGCP,PLEKHA8,PRNP,ZNF780A,CAPZB,WEE1,TMEM177,ZDHHC17,MRPL55,EZH1,NDUFB3,HRAS,SS18,CBFB,ZC3H18,YIPF6,VWA3B,RPL7L1,ZBTB20,SMYD3,TTC26,PRRC1,TMEM33,LMF2,PLRG1,SLC35A4,FAM92A,INTS8,PTDSS1,JAK3,RNPC3,RBM4B,CCS,HSPA8,PHC1,GRAMD1A,MA

				RVELD1,TMEM258,TUBA1C,LDHA,ZNF362,ATXN2,BRF1,DTX3,SMARCC2,TRIAP1,TGIF2LX,ARPP19,MYL12A,POP4,UQCR11,CHMP2A,SETD1B,TBC1D7,DNAH11,NUDT3,FRK,NOMO2,TOX4,SLC25A14,IRAK4,MAGEA6,PCGF2,LEPROT,COBLL1,ACTB,EYA1,PNISR,MYO1C,SLC35B1,MRTFA,MED19,NIF3L1,TSPY9P
GO:0043231	Intracellular membrane-bounded organelle	0.11	9.77E-09	RHBDD2,APPBP2,PPP2R5B,RRN3,CDC34,TCN2,MYH9,ZFYVE21,AURKA,RP2,CDIPT,HAS1,CHMP5,TNFAIP1,TUSC2,KALRN,SEC61A1,ALKBH7,NSRP1,YEATS4,MBD4,HBA2,MRPL34,LSM7,TERF2,KRAS,LAMTOR5,APC,PNPLA8,MDM2,BRIP1,KIF13A,GCHFR,MIB1,TRAM1,PLD2,ITPKC,ASF1B,MED26,PNO1,SLC9A1,EHBP1,TUBB4A,POLQ,ALG10,PRUNE1,SFXN5,PNKD,RNF7,SIGMAR1,ZFP36L2,ITGA1,MED7,MTERF3,CCNB2,MRPL17,MITD1,RFX5,ARL5A,CCNL1,MON1A,MICALL2,VIRMA,ZNF22,RPUSD4,POLR3E,PPIB,SOX7,LDHAL6B,KLF13,DERPC,PGK2,DDX19A,EIF2AK3,UTP23,RCE1,LRRC7,KLF7,NARF,GLS2,SEC22A,AQP1,TNKS,CC2D1A,SPHK1,CD46,AHCYL2,ZNF609,MYEF2,COX5A,MRFAP1,CAMK2G,DEF6,LYPLA1,MYO1D,STK11,TUBA3D,TMEM39A,ZBTB11,SMDT1,CNGA2,GLRX5,KDELR1,SPPL2C,FLRT2,UQCR10,EMC10,SIRT6,ATG101,CIR1,SMARCB1,STAT5A,USP9Y,KLHL7,NBR1,ICMT,ANP32B,DMPK,LAGE3,PFN3,NLRC3,PHLDB1,MT-CO1,RNF121,ZNF318,MT-CO3,TSEN15,GNPAT,SDHC,NUS1,AP4B1,ATP5PB, ATP5MD,ARMH3,SLC35A3,OMA1,PLPP3,FRA10AC1,DDX31,DOLPP1,CHCHD1,PKIG,TOR2A,UGCG,CSGALNACT2,SLC39A7,TAP2,NCBP1,UPF3A,STK19,NMNAT1,CTNNBIP1,KIN,APTX,CENPJ,DNAJC19,MRPL2,TSN,DYNLL1,GALNT3,TRAF3,SNX11,GPS2,ATN1,SLC35E3,DOP1B,ZDHHC20,CBX6,ZBED8,TONSL,ZC3H6,TMEM131L,BRMS1,URGCP,PLEKHA8,PRNP,ZNF780A,WEE1,TMEM177,ZDHHC17,MRPL55,EZH1,NDUFB3,HRAS,SS18,CBFB,ZC3H18,YIPF6,VWA3B,RPL7L1,ZBTB20,SMYD3,PRRC1,TMEM33,LMF2,PLRG1,SLC35A4,FAM92A,INTS8,PTDSS1,JAK3,RNPC3,RBM4B,CCS,HSPA8,PHC1,GRAMD1A,MARVELD1,TMEM258,TUBA1C,LDHA,ZNF362,ATXN2,BRF1,DTX3,SMARCC2,TRIAP1,TGIF2LX,ARPP19,POP4,UQCR11,CHMP2A,SETD1B,TBC1D7,NUDT3,F

				RK,NOMO2,TOX4,SLC25A14,IRAK4,MAGEA6,PCGF2,LEPROT,ACTB,EYA1,PNISR, MYO1C,SLC35B1,MRTFA,MED19,NIF3L1,TSPY9P
GO:0043226	Organelle	0.08	2.41E-07	RHBDD2,APPBP2,PPP2R5B,RRN3,CDC34,TCN2,MYH9,ZFYVE21,AURKA,RP2,CDIPT, HAS1,CHMP5,TNFAIP1,GNB3,TUSC2,PFN2,KALRN,SEC61A1,ALKBH7,NSRP1,YEATS4, MBD4,HBA2,MRPL34,LSM7,TERF2,KRAS,LAMTOR5,APC,PNPLA8,MDM2,BRIP1,KIF13A,GCHFR,MIB1,TRAM1,PLD2,ITPKC,ASF1B,MED26,PNO1,SLC9A1,EHBP1,TUBB4A,POLQ, ICAM1,ALG10,PRUNE1,SFXN5,PNKD,RNF7,SIGMAR1,MYL9,ZFP36L2,ITGA1,MED7, MTERF3,CCNB2,MRPL17,MITD1,RFX5,ARL5A,CCNL1,MON1A,MICALL2,VIRMA, DNAAF2,ZNF22,RPUSD4,POLR3E,PPIB,SOX7,LDHAL6B,KLF13,DERPC,PGK2,DDX19A,EIF2AK3,MYL1,UTP23,RCE1,LRRC7,KLF7,NARF,SLC5A6,GLS2,SEC22A,AQP1,TNKS,CC2D1A,SPHK1,CD46,SSH1,AHCYL2,ZNF609,MYEF2,COX5A,MRFAP1,CAMK2G,DEF6,LY-PLA1,MYO1D,STK11,TUBA3D,TMEM39A,ZBTB11,SMDT1,CNGA2,GLRX5,KDELR1,SPPL2C,FLRT2,UQCR10,EMC10,SIRT6,ATG101,CIR1,PPL,SMARCB1,STAT5A,USP9Y,KLHL7,NBR1,ICMT,ANP32B,DMPK,TTC30A,DYNLRB1,LAGE3,PFN3,NLRC3,PCNT,G3BP2,PHLDB1,MT-CO1,RNF121,ZNF318,MT-CO3,TSEN15,GNPAT,SDHC,NUS1,AP4B1,ATP5PB, ATP5MD,ARMH3,SLC35A3,OMA1,PLPP3,FRA10AC1,DDX31,DOLPP1,CHCHD1,PKIG,TOR2A,TRAF3IP1,UGCG,CSGALNACT2,SLC39A7,TAP2,NCBP1,UPF3A,STK19,NMNAT1, CTNNBIP1,KIN,APTX,CENPJ,DNAJC19,MRPL2,TSN,DYNLL1,GALNT3,TRAF3,SNX11,GPS2,ATN1,SLC35E3,DOP1B,ZDHHC20,CBX6,ZBED8,TONSL,ZC3H6,TMEM131L,MTMR11, BRMS1,URGCP,PLEKHA8,PRNP,ZNF780A,CAPZB,WEE1,TMEM177,ZDHHC17,MRPL55, EZH1,NDUFB3,HRAS,SS18,CBFB,ZC3H18,YIPF6,VWA3B,RPL7L1,ZBTB20,SMYD3,TTC26, PRRC1,TMEM33,LMF2,PLRG1,SLC35A4,FAM92A,INTS8,PTDSS1,JAK3,RNPC3,RBM4B,CCS,HSPA8,PHC1,GRAMD1A,MARVELD1,TMEM258,TUBA1C,LDHA,ZNF362,ATXN2, BRF1,DTX3,SMARCC2,TRIAP1,TGIF2LX,ARPP19,MYL12A,POP4,UQCR11,CHMP2A,SETD1B,TBC1D7,DNAH11,NUDT3,FRK,NOMO2,TOX4,SLC25A14,IRAK4,MAGEA6,PCGF2,LEPROT,COBLL1,ACTB,EYA1,PNISR,MYO1C,SLC35B1,MRTFA,MED19,NIF3L1,TSPY9P

GO:0005737	Cytoplasm	0.1	7.48E-07	RHBDD2,APPBP2,PPP2R5B,CDC34,TCN2,MYH9,ZFYVE21,AURKA,RP2,CDIPT,CKM,HAS1,CHMP5,TNFAIP1,GNB3,TUSC2,PFN2,KALRN,SEC61A1,ALKBH7,HBA2,TRPC4AP,MRPL34,LSM7,TERF2,KRAS,LAMTOR5,APC,PNPLA8,MDM2,BRIP1,KIF13A,GCHFR,MIB1,TRAM1,PLD2,ITPKC,PNO1,SLC9A1,EHBP1,TUBB4A,POLQ,ALG10,PRUNE1,SFXN5,PNKD,RNF7,SIGMAR1,MYL9,ZFP36L2,ITGA1,RASA2,MTERF3,CCNB2,MRPL17,MITD1,ARL5A,MON1A,MICALL2,VIRMA,DNAAF2,RPUSD4,POLR3E,PPIB,SPRYD3,SOX7,LDHAL6B,PGK2,DDX19A,EIF2AK3,MYL1,RCE1,LRRC7,KLF7,GLS2,SEC22A,AQP1,ACOT1,TNKS,CC2D1A,SPHK1,CD46,SSH1,AHCYL2,MYEF2,COX5A,MRFAP1,CAMK2G,DEF6,LYPLA1,MYO1D,STK11,TUBA3D,TMEM39A,RNF166,SMDT1,CNGA2,GLRX5,KDELR1,SPPL2C,FLRT2,UQCR10,EMC10,SIRT6,ATG101,CIR1,PPL,STAT5A,USP9Y,KLHL7,NBR1,ICMT,ANP32B,DMPK,TTC30A,OSCP1,DYNLRB1,LAGE3,PFN3,NLRC3,PCNT,G3BP2,PHLDB1,MT-CO1,RNF121,MAP3K3,ZNF318,MT-CO3,GNPAT,SDHC,NUS1,AP4B1, ATP5PB,ATP5MD,ARMH3,SLC35A3,GCLM,OMA1,PLPP3,DDX31,DOLPP1,CHCHD1,PKIG,TOR2A,TRAF3IP1,UGCG,CSGALNACT2,SLC39A7,TAP2,NCBP1,UPF3A,CTNNBIP1,KIN,APTX,CENPJ,DNAJC19,MRPL2,TSN,DYNLL1,GALNT3,TRAF3,SNX11,GPS2,ATN1,SLC35E3,PRPS2,DOP1B,ZDHHC20,TONSL,TMEM131L,MTMR11,PLEKHG2,BRMS1,URGCP,PLEKHA8,PRNP,PPIP5K1,CAPZB,HECTD2,WEE1,TMEM177,ZDHHC17,MRPL55,NDUFB3,HRAS,YIPF6,VWA3B,RPL7L1,BTBD9,ZBTB20,SMYD3,IQCJ-SCHIP1,PRRC1,TMEM33,LMF2,SLC35A4,PRPS1L1,FAM92A, PTDSS1,JAK3,RBM4B,CCS,HSPA8,GRAMD1A,MARVELD1,RSPRY1,TMEM258,TUBA1C,LDHA,ATXN2,DTX3,TRIAP1,PTGR2,ARPP19,MYL12A,UQCR11,CHMP2A,TBC1D7,DNAH11,NUDT3,FRK,NOMO2,SLC25A14,ZFAND6,IRAK4,LEPROT,ACTB,EYA1,PNISR,MYO1C,SLC35B1,MRTFA,NIF3L1,TSPY9P,CNK3/IPCEF1
GO:0031090	Organelle membrane	0.23	1.71E-05	RHBDD2,APPBP2,CDIPT,CHMP5,SEC61A1,YEATS4,MRPL34,KRAS,LAMTOR5,PNPLA8,MDM2,BRIP1,KIF13A,GCHFR,TRAM1,PLD2,SLC9A1,ALG10,SFXN5,SIGMAR1,MTERF3,MRPL17,MITD1,EIF2AK3,RCE1,SLC5A6,SEC22A,AQP1,TNKS,SPHK1,CD46,COX5A,CAMK2G,LYPLA1,TMEM39A,SMDT1,CNGA2,KDELR1,SPPL2C,FLRT2,

				UQCR10,EMC10,ATG101,NBR1,ICMT,DMPK,MT-CO1,RNF121,MT-CO3,GNPAT,SDHC, NUS1,AP4B1,ATP5PB,ATP5MD,ARMH3, SLC35A3,OMA1,PLPP3,DOLPP1,CHCHD1, UGCG,CSGALNACT2,SLC39A7,TAP2,DNAJC19,MRPL2,DYNLL1,GALNT3,DOP1B, ZDHHC20,PLEKHA8,PRNP,TMEM177,ZDHHC17,MRPL55,NDUFB3,HRAS,YIPF6,TMEM33,LMF2,PLRG1,SLC35A4,FAM92A,PTDSS1,HSPA8,GRAMD1A,TMEM258,UQCR11,CHMP2A,NOMO2,SLC25A14,IRAK4,LEPROT,MYO1C,SLC35B1
GO:0005654	Nucleoplasm	0.21	3.35E-05	RHBDD2,APPBP2,RRN3,CDC34,MYH9,AURKA,RP2,KALRN,NSRP1,YEATS4,MBD4, LSM7,TERF2,APC,MDM2,BRIP1,GCHFR,ITPKC,ASF1B,MED26,PNO1,SLC9A1, EHBP1,POLQ,RNF7,MED7,RFX5,CCNL1,VIRMA,ZNF22,RPUSD4,POLR3E,PPIB,SOX7,DERPC,DDX19A,LRRC7,KLF7,NARF,TNKS,ZNF609,MRFAP1,CAMK2G,DEF6,STK11,ZBTB11,SMDT1,SIRT6,CIR1,SMARCB1,STAT5A,KLHL7,NBR1,ANP32B,LAGE3,ZNF318,TSEN15,CHCHD1,SLC39A7,TAP2,NCBP1,UPF3A,STK19,NMNAT1,CTNNBIP1,KIN,APTX,MRPL2,TSN,GPS2,ATN1,CBX6,ZBED8,TONSL,BRMS1,PLEKHA8,WEE1,EZH1,HRAS,CBFB,ZC3H18,VWA3B,ZBTB20,SMYD3,PLRG1,INTS8,RNPC3,RBM4B,HSPA8,PHC1,BRF1,DTX3,SMARCC2,TRIAP1,ARPP19,POP4,SETD1B,FRK,PCGF2,ACTB,EYA1,PNISR,MYO1C,MRTFA
GO:0032991	Protein-containing complex	0.17	8.45E-05	RHBDD2,APPBP2,PPP2R5B,MYH9,AURKA,CHMP5,TNFAIP1,GNB3,SEC61A1, NSRP1,YEATS4,HBA2,TRPC4AP,MRPL34,LSM7,TERF2,LAMTOR5,APC,MDM2,BRIP1,KIF13A,GCHFR,ASF1B,MED26,SLC9A1,EHBP1,RNF7,MYL9,ITGA1,MED7,CCNB2,MRPL17,RFX5,CCNL1,MON1A,VIRMA,DNAAF2,POLR3E,PPIB,LDHAL6B,MYL1,UTP23,TNKS,MYEF2,COX5A,CAMK2G,MYO1D,STK11,SMDT1,CNGA2,GLRX5,UQCR10,EMC10,ATG101,CIR1,SMARCB1,STAT5A,KLHL7,NBR1,TTC30A,DYNLRB1,LAGE3,G3BP2,MT-CO1,MT-CO3,SDHC,NUS1,AP4B1,ATP5PB,ATP5MD, GCLM,CHCHD1,TRAF3IP1,TAP2,NCBP1, UPF3A,CTNNBIP1,KIN,CENPJ,DNAJC19,MRPL2,TSN,DYNLL1,TRAF3,GPS2,PRPS2,CBX6,BRMS1,CAPZB,MRPL55,EZH1,NDUFB3,HRAS,SS18,CBFB,ZC3H18,RPL7L1,TTC26,PLRG1,PRPS1L1,INTS8,RNPC3,RBM4B,HSPA8,PHC1,PACC1,TMEM258,LDHA,ATXN2,BRF1,SM

				ARCC2,TRIAP1,MYL12A,POP4,UQCR11,CHMP2A,SETD1B,TBC1D7,DNAH11,NOMO2,TOX4,PCGF2,ACTB,EYA1,MYO1C,MED19
GO:0031981	Nuclear lumen	0.19	0.00013	RHBDD2,APPBP2,RRN3,CDC34,MYH9,AURKA,RP2,KALRN,NSRP1,YEATS4,MBD4,LSM7,TERF2,APC,MDM2,BRIP1,GCHFR,ITPKC,ASF1B,MED26,PNO1,SLC9A1,EHBP1,POLQ,RNF7,MED7,RFX5,CCNL1,VIRMA,ZNF22,RPUSD4,POLR3E,PPIB,SOX7,DERPC,DDX19A,UTP23,LRRC7,KLF7,NARF,TNKS,CC2D1A,ZNF609,MRFAP1,CAMK2G,DEF6,STK11,ZBTB11,SMDT1,SIRT6,CIR1,SMARCB1,STAT5A,KLHL7,NBR1,ANP32B,LAGE3,ZNF318,TSEN15,DDX31,CHCHD1,SLC39A7,TAP2,NCBP1,UPF3A,STK19,NMNAT1,CTNNBIP1,KIN,APTX,MRPL2,TSN,GPS2,ATN1,CBX6,ZBED8,TONSL,BRMS1,PLEKHA8,WEE1,EZH1,HRAS,CBFB,ZC3H18,VWA3B,RPL7L1,ZBTB20,SMYD3,PLRG1,INTS8,RNPC3,RBM4B,HSPA8,PHC1,BRF1,DTX3,SMARCC2,TRIAP1,ARPP19,POP4,SETD1B,FRK,PCGF2,ACTB,EYA1,PNISR,MYO1C,MRTFA
GO:0031967	Organelle envelope	0.33	0.00061	CHMP5,YEATS4,MRPL34,KRAS,PNPLA8,BRIP1,GCHFR,SFXN5,SIGMAR1,MTERF3,MRPL17,AQP1,TNKS,COX5A,LYPLA1,SMDT1,UQCR10,NBR1,DMPK,MT-CO1,MT-CO3,GNPAT,SDHC, ATP5PB,ATP5MD,OMA1,CHCHD1,TOR2A,DNAJC19,MRPL2,PRNP,TMEM177,MRPL55,NDUFB3,TMEM33,PLRG1,FAM92A,TRIAP1,UQCR11,CHMP2A,SLC25A14,MYO1C
GO:1902494	Catalytic complex	0.31	0.00061	RHBDD2,APPBP2,PPP2R5B,TNFAIP1,GNB3,YEATS4,TRPC4AP,LSM7,RNF7,MED7,CCNB2,CCNL1,VIRMA,POLR3E,LDHAL6B,STK11,UQCR10,ATG101,SMARCB1,KLHL7,DYNLRB1,MT-CO1,SDHC,NUS1,GCLM,TSN,DYNLL1,TRAF3,PRPS2,BRMS1,EZH1,NDUFB3,HRAS,SS18,PLRG1,PRPS1L1,PHC1,TMEM258,LDHA,SMARCC2,POP4,UQCR11,SETD1B,DNAH11,TOX4,PCGF2,ACTB,MYO1C
GO:0031984	Organelle sub-compartment	0.3	0.00088	RHBDD2,CDIPT,SEC61A1,KRAS,PNPLA8,KIF13A,TRAM1,PLD2,SLC9A1,ALG10,SIGMAR1,ARL5A,EIF2AK3,RCE1,SEC22A,CAMK2G,TMEM39A,KDELR1,SPPL2C,FLRT2,EMC10,ATG101,ICMT,DMPK,RNF121,NUS1,AP4B1,PLPP3,DOLPP1,CSGALNACT2,

				SLC39A7,TAP2,GALNT3,DOP1B,ZDHHC20,PLEKHA8,HRAS,YIPF6,TMEM33,LMF2,PTDS S1,GRAMD1A,TMEM258,ATXN2,NOMO2,SLC35B1
GO:0005 634	Nucleus	0.12	0.0013	RHBDD2,APPBP2,PPP2R5B,RRN3,CDC34,MYH9,AURKA,RP2,CHMP5,TNFAIP1, KALRN,NSRP1,YEATS4,MBD4,LSM7,TERF2,APC,MDM2,BRIP1,GCHFR,ITPKC, ASF1B,MED26,PNO1,SLC9A1,EHBP1,TUBB4A,POLQ,PRUNE1,PNKD,RNF7,SIGMAR1, ZFP36L2,MED7,CCNB2,RFX5,CCNL1,VIRMA,ZNF22,RPUSD4,POLR3E,PPIB,SOX7,LDHA L6B,KLF13,DERPC,PGK2,DDX19A,UTP23,LRRC7,KLF7,NARF,AQP1,TNKS,CC2D1A,SPH K1,ZNF609,MYEF2,MRFAP1,CAMK2G,DEF6,LYPLA1,STK11,TUBA3D,ZBTB11,SMDT1, GLRX5,SIRT6,CIR1,SMARCB1,STAT5A,USP9Y,KLHL7,NBR1,ANP32B,DMPK,LAGE3,PFN 3,ZNF318,TSEN15,ATP5PB,FRA10AC1,DDX31,CHCHD1,PKIG,TOR2A,SLC39A7,TAP2,NC BP1,UPF3A,STK19,NMNAT1,CTNNBIP1,KIN,APTX,CENPJ,MRPL2,TSN,DYNLL1,GPS2,A TN1,CBX6,ZBED8,TONSL,ZC3H6,BRMS1,URGCP,PLEKHA8,PRNP,ZNF780A,WEE1,EZH1 ,HRAS,SS18,CBFB,ZC3H18,VWA3B,RPL7L1,ZBTB20,SMYD3,TMEM33,PLRG1,FAM92A,IN TS8,RNPC3,RBM4B,CCS,HSPA8,PHC1,MARVELD1,TUBA1C,LDHA,ZNF362,BRF1,DTX3, SMARCC2,TRIAP1,TGIF2LX,ARPP19,POP4,CHMP2A,SETD1B,NUDT3,FRK,TOX4,IRAK4, MAGEA6,PCGF2,ACTB,EYA1,PNISR,MYO1C,MRTFA,MED19,NIF3L1,TSPY9P
GO:0031 300	Intrinsic component of organelle membrane	0.49	0.002	RHBDD2,SEC61A1,TRAM1,SFXN5,EIF2AK3,RCE1,SMDT1,SPPL2C,EMC10,DMPK,SLC35A 3,DOLPP1,CSGALNACT2,TAP2,ZDHHC20,TMEM177,TMEM33,SLC35A4,GRAMD1A, SLC35B1
GO:0031 301	Integral component of organelle membrane	0.5	0.002	RHBDD2,SEC61A1,TRAM1,SFXN5,EIF2AK3,RCE1,SMDT1,SPPL2C,EMC10,DMPK,SLC35A 3,DOLPP1,CSGALNACT2,TAP2,ZDHHC20,TMEM177,TMEM33,SLC35A4,SLC35B1
GO:0070 013	Intracellular organelle lumen	0.15	0.002	RHBDD2,APPBP2,RRN3,CDC34,TCN2,MYH9,AURKA,RP2,KALRN,ALKBH7,NSRP1, YEATS4,MBD4,HBA2,MRPL34,LSM7,TERF2,APC,MDM2,BRIP1,GCHFR,IT- PKC,ASF1B,MED26,PNO1,SLC9A1,EHBP1,POLQ,RNF7,MED7,MRPL17,RFX5,CCNL1,VIR

				MA,ZNF22,RPUSD4,POLR3E,PPIB,SOX7,LDHAL6B,DERPC,DDX19A,UTP23,LRRC7,KLF7,NARF,GLS2,TNKS,CC2D1A,ZNF609,MRFAP1,CAMK2G,DEF6,STK11,ZBTB11,SMDT1,GLRX5,SIRT6,CIR1,SMARCB1,STAT5A,KLHL7,NBR1,ANP32B,LAGE3,ZNF318,TSEN15,GNPAT,AP4B1,ATP5PB,DDX31,CHCHD1,TOR2A,SLC39A7,TAP2,NCBP1,UPF3A,STK19,NMNAT1,CTNNBIP1,KIN,APTX,DNAJC19,MRPL2,TSN,GPS2,ATN1,CBX6,ZBED8,TONSL,BRMS1,PLEKHA8,WEE1,MRPL55,EZH1,HRAS,CBFB,ZC3H18,VWA3B,RPL7L1,ZBTB20,SMYD3,PLRG1,INTS8,RNPC3,RBM4B,HSPA8,PHC1,BRF1,DTX3,SMARCC2,TRIAP1,ARPP19,POP4,SETD1B,FRK,PCGF2,ACTB,EYA1,PNISR,MYO1C,MRTFA
GO:0098798	Mitochondrial protein-containing complex	0.54	0.0024	MRPL34,MRPL17,COX5A,SMDT1,UQCR10,MT-CO1,MT-CO3,SDHC,ATP5PB,ATP5MD,CHCHD1,DNAJC19,MRPL2,MRPL55,NDUFB3,UQCR11
GO:0005740	Mitochondrial envelope	0.37	0.0027	MRPL34,KRAS,PNPLA8,SFXN5,MTERF3,MRPL17,COX5A,SMDT1,UQCR10,NBR1,DMPK,MT-CO1,MT-CO3,GNPAT,SDHC,ATP5PB,ATP5MD,OMA1,CHCHD1,DNAJC19,MRPL2,PRNP,TMEM177,MRPL55,NDUFB3,FAM92A,TRIAP1,UQCR11,SLC25A14
GO:0005789	Endoplasmic reticulum membrane	0.32	0.0027	RHBDD2,CDIPT,SEC61A1,KRAS,PNPLA8,TRAM1,PLD2,SLC9A1,ALG10,SIGMAR1,EIF2AK3,RCE1,SEC22A,CAMK2G,TMEM39A,KDELR1,SPPL2C,FLRT2,EMC10,ATG101,ICMT,DMPK,RNF121,NUS1,PLPP3,DOLPP1,SLC39A7,TAP2,ZDHHC20,HRAS,TMEM33,LMF2,PTDSS1,GRAMD1A,TMEM258,NOMO2,SLC35B1
GO:0031227	Intrinsic component of endoplasmic reticulum membrane	0.65	0.0027	RHBDD2,SEC61A1,TRAM1,EIF2AK3,RCE1,SPPL2C,EMC10,DOLPP1,TAP2,TMEM33,GRAMD1A,SLC35B1

GO:0005743	Mitochondrial inner membrane	0.43	0.0042	MRPL34,SFXN5,MRPL17,COX5A,SMDT1,UQCR10,MT-CO1,MT-CO3,SDHC,ATP5PB, ATP5MD,OMA1,CHCHD1,DNAJC19,MRPL2,TMEM177,MRPL55,NDUFB3,FAM92A,UQCR11,SLC25A14
GO:0098800	Inner mitochondrial membrane protein complex	0.65	0.0042	COX5A,SMDT1,UQCR10,MT-CO1,MT-CO3,SDHC,ATP5PB,ATP5MD,DNAJC19,NDUFB3, UQCR11
GO:0031966	Mitochondrial membrane	0.37	0.0044	MRPL34,KRAS,PNPLA8,SFXN5,MTERF3,MRPL17,COX5A,SMDT1,UQCR10,DMPK,MT-CO1,MT-CO3,GNPAT,SDHC,ATP5PB,ATP5MD,OMA1,CHCHD1,DNAJC19,MRPL2,PRNP, TMEM177,MRPL55,NDUFB3,FAM92A,UQCR11,SLC25A14
GO:0030176	Integral component of endoplasmic reticulum membrane	0.63	0.0062	RHBDD2,SEC61A1,TRAM1,EIF2AK3,RCE1,SPPL2C,EMC10,DOLPP1,TAP2,TMEM33,SLC35B1
GO:0019866	Organelle inner membrane	0.4	0.0068	MRPL34,SFXN5,SIGMAR1,MRPL17,COX5A,SMDT1,UQCR10,MT-CO1,MT-CO3,SDHC, ATP5PB,ATP5MD,OMA1,CHCHD1,DNAJC19,MRPL2,TMEM177,MRPL55,NDUFB3,FAM92A,UQCR11,SLC25A14
GO:0071564	npBAF complex	1.27	0.0087	SMARCB1,SS18,SMARCC2,ACTB
GO:0016604	Nuclear body	0.32	0.0194	CDC34,MYH9,RP2,NSRP1,MBD4,TERF2,ITPKC,MED7,CCNL1,VIRMA,TNKS,CIR1, NBR1,LAGE3,TAP2,STK19,NMNAT1,TONSL,ZC3H18,ZBTB20,PLRG1,RBM4B,SETD1B,PCGF2,EYA1,PNISR,MYO1C
GO:0016459	Myosin complex	0.84	0.0212	MYH9,MYL9,MYL1,MYO1D,MYL12A,MYO1C

GO:0070069	Cytochrome complex	0.91	0.0341	COX5A,UQCR10,MT-CO1,MT-CO3,UQCR11
GO:0098803	Respiratory chain complex	0.7	0.0397	COX5A,UQCR10,MT-CO1,MT-CO3,SDHC,NDUFB3,UQCR11
GO:0012505	Endomembrane system	0.13	0.04	RHBDD2,TCN2,MYH9,ZFYVE21,RP2,CDIPT,HAS1,CHMP5,TNFAIP1,SEC61A1,YEATS4,KRAS,LAMTOR5,APC,PNPLA8,BRIP1,KIF13A,GCHFR,TRAM1,PLD2,SLC9A1,EHBP1,POLQ,ALG10,SIGMAR1,ITGA1,MITD1,ARL5A,MON1A,MICALL2,PPIB,EIF2AK3,RCE1,LRRC7,SEC22A,AQP1,TNKS,SPHK1,CD46,AHCYL2,CAMK2G,LYPLA1,MYO1D,TMEM39A,KDELR1,SPPL2C,FLRT2,EMC10,SIRT6,ATG101,NBR1,ICMT,DMPK,RNF121,NUS1,AP4B1,ARMH3,SLC35A3,PLPP3,DDX31,DOLPP1,TOR2A,UGCG,CSGALNACT2,SLC39A7,TAP2,TSN,DYNLL1,GALNT3,TRAF3,SNX11,SLC35E3,DOP1B,ZDHHC20,TMEM131L,PLEKHA8,PRNP,ZDHHC17,HRAS,YIPF6,PRRC1,TMEM33,LMF2,PLRG1,SLC35A4,PTDSS1,JAK3,HSPA8,GRAMD1A,TMEM258,ATXN2,CHMP2A,FRK,NOMO2,IRAK4,LEPROT,MYO1C,SLC35B1
GO:0005739	Mitochondrion	0.22	0.0465	TUSC2,ALKBH7,MRPL34,KRAS,PNPLA8,SLC9A1,SFXN5,PNKD,MTERF3,MRPL17,RPUSD4,LDHAL6B,GLS2,COX5A,STK11,SMDT1,GLRX5,UQCR10,NBR1,DMPK,MT-CO1,MT-CO3,GNPAT,SDHC,ATP5PB,ATP5MD,OMA1,CHCHD1,NCBP1,DNAJC19,MRPL2,DYNLL1,TRAF3,GPS2,PRNP,TMEM177,MRPL55,NDUFB3,FAM92A,TRIAP1,UQCR11,SLC25A14,NIF3L1
GO:0005746	Mitochondrial respirasome	0.68	0.0466	COX5A,UQCR10,MT-CO1,MT-CO3,SDHC,NDUFB3,UQCR11
GO:0140092	bBAF complex	1.29	0.0466	SMARCB1,SMARCC2,ACTB

hsa05014	Amyotrophic lateral sclerosis	0.57	0.00037	TUBB4A,SIGMAR1,EIF2AK3,COX5A,TUBA3D,UQCR10,ATG101,PFN3,MT-CO1,MT-CO3,SDHC,ATP5PB,NCBP1,NDUFB3,CCS,TUBA1C,ATXN2,UQCR11,DNAH11,ACTB
hsa04714	Thermogenesis	0.57	0.0134	KRAS,COX5A,UQCR10,SIRT6,MT-CO1,MT-CO3,SDHC,ATP5PB,NDUFB3,HRAS, SMARCC2, UQCR11,ACTB
hsa05020	Prion disease	0.54	0.0134	TUBB4A,EIF2AK3,COX5A,TUBA3D,UQCR10,MT-CO1,MT-CO3,SDHC,ATP5PB,PRNP, NDUFB3,HSPA8,TUBA1C,UQCR11
hsa05010	Alzheimer disease	0.47	0.0156	KRAS,APC,TUBB4A,EIF2AK3,COX5A,TUBA3D,UQCR10,ATG101,MT-CO1,MT-CO3,SDHC, ATP5PB,NDUFB3,HRAS,TUBA1C,UQCR11
hsa05012	Parkinson disease	0.52	0.0293	TUBB4A,EIF2AK3,COX5A,TUBA3D,UQCR10,MT-CO1,MT-CO3,SDHC,ATP5PB,NDUFB3, TUBA1C,UQCR11
hsa04810	Regulation of actin cytoskeleton	0.53	0.032	MYH9,KRAS,APC,SLC9A1,MYL9,ITGA1,SSH1,PFN3,HRAS,MYL12A,ACTB
hsa00190	Oxidative phosphorylation	0.61	0.0438	COX5A,UQCR10,MT-CO1,MT-CO3,SDHC,ATP5PB,NDUFB3,UQCR11
hsa05016	Huntington disease	0.46	0.0438	TUBB4A,COX5A,TUBA3D,UQCR10,ATG101,MT-CO1,MT-CO3,SDHC,ATP5PB,NDUFB3, TUBA1C,UQCR11,DNAH11
hsa05230	Central carbon metabolism in cancer	0.76	0.0438	KRAS,LDHAL6B,GLS2,SIRT6,HRAS,LDHA
HSA-5620924	Intraflagellar transport	0.99	0.0087	TUBB4A,TUBA3D,TTC30A,DYNLRB1,TRAF3IP1,DYNLL1,TTC26,TUBA1C

HSA-1632852	Macroautophagy	0.73	0.0148	LAMTOR5,TUBB4A,MTERF3,TUBA3D,ATG101,NBR1,PCNT,DYNLL1,HSPA8,TUBA1C,CHMP2A
HSA-9648002	RAS processing	1.13	0.0332	KRAS,RCE1,LYPLA1,ICMT,HRAS
HSA-9663891	Selective autophagy	0.81	0.0332	TUBB4A,MTERF3,TUBA3D,NBR1,PCNT,DYNLL1,HSPA8,TUBA1C
HSA-5617833	Cilium Assembly	0.59	0.0391	RP2,TUBB4A,TUBA3D,CNGA2,TTC30A,DYNLRB1,PCNT,TRAF3IP1,CENPJ,DYNLL1,TTC26,TUBA1C
HSA-1428517	The citric acid (TCA) cycle and respiratory electron transport	0.6	0.0485	LDHAL6B,COX5A,UQCR10,MT-CO1,MT-CO3,SDHC,ATP5PB,NDUFB3,LDHA,UQCR11,SLC25A14
WP111	Electron transport chain: OXPHOS system in mitochondria	0.75	0.0419	COX5A,UQCR10,MT-CO1,MT-CO3,SDHC,ATP5PB,NDUFB3,UQCR11,SLC25A14
BTO:0000000	Tissues, cell types and enzyme sources	0.08	6.85E-11	RHBDD2,APPBP2,TMEM161A,PPP2R5B,RRN3,CDC34,TCN2,MYH9,FAM118A,ZFYVE21,AURKA,RP2,CDIPT,CKM,HAS1,CHMP5,TNFAIP1,GNB3,TUSC2,PFN2,KALRN,SEC61A1,ALKBH7,MBD4,HBA2,TRPC4AP,MRPL34,TERF2,KRAS,LAMTOR5,APC,PNPLA8,MDM2,KIF13A,GCHFR,MIB1,TRAM1,MAST3,PLD2,ITPKC,ASF1B,MED26,PNO1,SLC9A1,EHBP1,TUBB4A,POLQ,ICAM1,SEMA3A,DEF8,PRUNE1,SFXN5,PNKD,RNF7,SIGMAR1,MYL9,ZFP36L2,ITGA1,MED7,RASA2,MTERF3,CCNB2,MRPL17,MITD1,RFX5,CZIB,ARL5A,CCNL1,MON1A,MICALL2,VIRMA,DNAAF2,ZNF22,RPUSD4,POLR3E,PPIB,SPRYD3,SOX7,LDHAL6B,KLF13,PGK2,DDX19A,EIF2AK3,MYL1,UTP23,PRS

				S1,RCE1,LRRC7,KLF7,NARF,SLC5A6,GLS2,SEC22A,AQP1,TNKS,CC2D1A,SPHK1,CD46,SSH1,AHCYL2,ZNF609,MYEF2,COX5A,MRFAP1,CAMK2G,DEF6,LYPLA1,MYO1D,STK11,TUBA3D,TMEM39A,RNF166,ZBTB11,SMDT1,CNGA2,GLRX5,KDELR1,SPPL2C,FLRT2,UQCR10,EMC10,SIRT6,ATG101,CIR1,PPL,UBE3B,SMARCB1,STAT5A,SYDE1,KLHL7,NBR1,ICMT,ANP32B,DMPK,TTC30A,OSCP1,DYNLRB1,LAGE3,MAP3K6,PFN3,NLRC3,PCNT,G3BP2,PHLDB1,MT-CO1,RNF121,MAP3K3,ZNF318,MT-CO3,TSEN15,GNPAT,SDHC,NUS1,AP4B1, ATP5PB,ATP5MD,SLC35A3,GCLM,OMA1,PLPP3,FRA10AC1,DDX31,ERI3,DOLPP1,CHCHD1,PKIG,TOR2A,TRAF3IP1,UGCG,CSGALNACT2,TMEM245,SLC39A7,NCBP1,UPF3A,STK19,GPR180,NMNAT1,CTNNBIP1,KIN,APTX,CENPJ,DNAJC19,MRPL2,TSN,DYNLL1,GALNT3,TRAF3,SNX11,HERPUD2,GPS2,ATN1,SLC35E3,PRPS2,DOP1B,ZDHHC20,CBX6,TONSL,ZC3H6,TMEM131L,MTMR11,PLEKHG2,BRMS1,URGCP,PLEKHA8,FAM133B,PRNP,PPIP5K1,ZNF780A,CAPZB,HECTD2,WEE1,TMEM177,ZDHHC17,MRPL55,EZH1,NT5DC2,NDUFB3,HRAS,SS18,CBFB,ZC3H18,YIPF6,VWA3B,RPL7L1,BTBD9,ZBTB20,SMYD3,TTC26,PRRC1,TMEM33,LMF2,PLRG1,SLC35A4,FAM92A,INTS8,PTDSS1,JAK3,RNPC3,RBM4B,TSKU,CCS,HSPA8,PHC1,PACC1,GRAMD1A,RSPRY1,TMEM258,TUBA1C,LDHA,ZNF362,ATXN2,BRF1,DTX3,SMARCC2,TRIAP1,PTGR2,TGIF2LX,ARPP19,MYL12A,POP4,UQCR11,CHMP2A,SETD1B,TBC1D7,DNAH11,NUDT3,FRK,NOMO2,TOX4,SLC25A14,ZFAND6,IRAK4,MAGEA6,PCGF2,LEPROT,COBLL1,ACTB,EYA1,PNISR,MYO1C,SLC35B1,MRTFA,MED19,NIF3L1
BTO:0000042	Animal	0.08	6.85E-11	RHBDD2,APPBP2,TMEM161A,PPP2R5B,RRN3,CDC34,TCN2,MYH9,FAM118A,ZFYVE21,AURKA,RP2,CDIPT,CKM,HAS1,CHMP5,TNFAIP1,GNB3,TUSC2,PFN2,KALRN,SEC61A1,ALKBH7,MBD4,HBA2,TRPC4AP,MRPL34,TERF2,KRAS,LAMTOR5,APC,PNPLA8,MDM2,KIF13A,GCHFR,MIB1,TRAM1,MAST3,PLD2,ITPKC,ASF1B,MED26,PNO1,SLC9A1,EHBP1,TUBB4A,POLQ,ICAM1,SEMA3A,DEF8,PRUNE1,SFXN5,PNKD,RNF7,SIG-MAR1,MYL9,ZFP36L2,ITGA1,MED7,RASA2,MTERF3,CCNB2,MRPL17,MITD1,RFX5,CZIB,ARL5A,CCNL1,MON1A,MICALL2,VIRMA,DNAAF2,ZNF22,RPUSD4,POLR3E,PPIB,

				SPRYD3,SOX7,LDHAL6B,KLF13,PGK2,DDX19A,EIF2AK3,MYL1,UTP23,PRSS1,RCE1,LRRC7,KLF7,NARF,SLC5A6,GLS2,SEC22A,AQP1,TNKS,CC2D1A,SPHK1,CD46,SSH1,AHCYL2,ZNF609,MYEF2,COX5A,MRFAP1,CAMK2G,DEF6,LYPLA1,MYO1D,STK11,TUBA3D,TMEM39A,RNF166,ZBTB11,SMDT1,CNGA2,GLRX5,KDELR1,SPPL2C,FLRT2,UQCR10,EMC10,SIRT6,ATG101,CIR1,PPL,UBE3B,SMARCB1,STAT5A,SYDE1,KLHL7,NBR1,ICMT,ANP32B,DMPK,TTC30A,OSCP1,DYNLRB1,LAGE3,MAP3K6,NLRC3,PCNT,G3BP2,PHLDB1,MT-CO1,RNF121,MAP3K3,ZNF318,MT-CO3,TSEN15,GNPAT,SDHC,NUS1,AP4B1,ATP5PB,ATP5MD,SLC35A3,GCLM,OMA1,PLPP3,FRA10AC1,DDX31,ERI3,DOLPP1,CHCHD1,PKIG,TOR2A,TRAF3IP1,UGCG,CSGALNACT2,TMEM245,SLC39A7,NCBP1,UPF3A,STK19,GPR180,NMNAT1,CTNNBIP1,KIN,APTX,CENPJ,DNAJC19,MRPL2,TSN,DYNLL1,GALNT3,TRAF3,SNX11,HERPUD2,GPS2,ATN1,SLC35E3,PRPS2,DOP1B,ZDHHC20,CBX6,TONSL,ZC3H6,TMEM131L,MTMR11,PLEKHG2,BRMS1,URGCP,PLEKHA8,FAM133B,PRNP,PPIP5K1,ZNF780A,CAPZB,HECTD2,WEE1,TMEM177,ZDHHC17,MRPL55,EZH1,NT5DC2,NDUFB3,HRAS,SS18,CBFB,ZC3H18,YIPF6,VWA3B,RPL7L1,BTBD9,ZBTB20,SMYD3,TTC26,PRRC1,TMEM33,LMF2,PLRG1,SLC35A4,FAM92A,INTS8,PTDSS1,JAK3,RNPC3,RBM4B,TSKU,CCS,HSPA8,PHC1,PACC1,GRAMD1A,RSPRY1,TMEM258,TUBA1C,LDHA,ZNF362,ATXN2,BRF1,DTX3,SMARCC2,TRIAP1,PTGR2,TGIF2LX,ARPP19,MYL12A,POP4,UQCR11,CHMP2A,SETD1B,TBC1D7,DNAH11,NUDT3,FRK,NOMO2,TOX4,SLC25A14,ZFAND6,IRAK4,MAGEA6,PCGF2,LEPROT,COBLL1,ACTB,EYA1,PNISR,MYO1C,SLC35B1,MRTFA,MED19,NIF3L1
BTO:0001489	Whole body	0.09	1.62E-06	RHBDD2,APPBP2,PPP2R5B,RRN3,CDC34,TCN2,MYH9,ZFYVE21,AURKA,CDIPT,CKM,HAS1,CHMP5,TNFAIP1,GNB3,TUSC2,PFN2,KALRN,SEC61A1,ALKBH7,MBD4,HBA2,TRPC4AP,TERF2,KRAS,LAMTOR5,APC,PNPLA8,MDM2,KIF13A,MIB1,TRAM1,MAST3,PLD2,ITPKC,ASF1B,MED26,PNO1,SLC9A1,EHBP1,TUBB4A,ICAM1,SEMA3A,DEF8,PRUNE1,SFXN5,PNKD,RNF7,SIGMAR1,MYL9,ZFP36L2,ITGA1,MED7,RASA2,MTERF3,CCNB2,MRPL17,RFX5,CZIB,ARL5A,CCNL1,MON1A,MICALL2,VIRMA,DNAAF2,ZNF22,

				RPUSD4,POLR3E,PPIB,SPRYD3,SOX7,LDHAL6B,KLF13,PGK2,DDX19A,EIF2AK3,MYL1,PRSS1,RCE1,LRRC7,KLF7,NARF,SLC5A6,GLS2,AQP1,TNKS,CC2D1A,SPHK1,CD46,SSH1,AHCYL2,ZNF609,MYEF2,COX5A,MRFAP1,CAMK2G,DEF6,LYPLA1,MYO1D,STK11, TUBA3D,TMEM39A,RNF166,SMDT1,CNGA2,GLRX5,KDELR1,SPPL2C,FLRT2,UQCR10,EMC10,SIRT6,ATG101,CIR1,PPL,UBE3B,SMARCB1,STAT5A,SYDE1,KLHL7,NBR1,ICMT,ANP32B,DMPK,TTC30A,OSCP1,DYNLRB1,LAGE3,MAP3K6,NLRC3,PCNT,G3BP2,PHLDB1,MT-CO1,RNF121,MAP3K3,MT-CO3,GNPAT,SDHC,NUS1,AP4B1,ATP5PB,ATP5MD, SLC35A3,GCLM,OMA1,PLPP3,ERI3,CHCHD1,PKIG,TOR2A,TRAF3IP1,UGCG,CSGAL-NACT2,TMEM245,SLC39A7,NCBP1,UPF3A,STK19,NMNAT1,CTNNBIP1,APTX,CENPJ,DNAJC19,TSN,DYNLL1,GALNT3,TRAF3,SNX11,HERPUD2,GPS2,ATN1,SLC35E3,PRPS2, ZDHHC20,CBX6,MTMR11,PLEKHG2,BRMS1,URGCP,PLEKHA8,PRNP,PPIP5K1,ZNF780A,CAPZB,WEE1,ZDHHC17,MRPL55,EZH1,NT5DC2,NDUFB3,HRAS,ZC3H18,VWA3B,RPL7L1,BTBD9,ZBTB20,SMYD3,TTC26,PRRC1,TMEM33,LMF2,PLRG1,SLC35A4,FAM92A,PTDSS1,JAK3,RNPC3,RBM4B,TSKU,CCS,HSPA8,PACC1,GRAMD1A,RSPRY1,TMEM258,TUBA1C,LDHA,ZNF362,ATXN2,BRF1,DTX3,SMARCC2,TRIAP1,TGIF2LX,ARPP19,MYL12A,POP4,UQCR11,CHMP2A,TBC1D7,DNAH11,NUDT3,FRK,NOMO2,TOX4,ZFAND6,IRAK4,MAGEA6,PCGF2,LEPROT,COBLL1,ACTB,EYA1,PNISR,MYO1C,SLC35B1,MRTFA,NIF3L1
BTO:0000083	Female reproductive system	0.15	0.0026	RHBDD2,APPBP2,RRN3,CDC34,MYH9,AURKA,CDIPT,TNFAIP1,TUSC2,KALRN, SEC61A1,ALKBH7,MBD4,HBA2,TRPC4AP,KRAS,LAMTOR5,PNPLA8,MDM2,MIB1,PLD2, ASF1B,MED26,SLC9A1,EHBP1,TUBB4A,PRUNE1,RNF7,SIGMAR1,MYL9,CCNB2,MRPL17, RFX5,ARL5A,CCNL1,MON1A,VIRMA,ZNF22,RPUSD4,PPIB,LDHAL6B,PGK2,DDX19A,EIF2AK3,RCE1,NARF,SLC5A6,AQP1,TNKS,CC2D1A,SPHK1,CD46,AHCYL2,MYEF2,MRFAP1,LYPLA1,MYO1D,STK11,TUBA3D,GLRX5,KDELR1,SPPL2C,EMC10,ATG101,SMARCB1, STAT5A,SYDE1,KLHL7,NBR1,ANP32B,TTC30A,OSCP1,DYNLRB1,G3BP2,PHLDB1,MT-CO1,MAP3K3,GNPAT,SDHC,NUS1,ATP5PB, SLC35A3,PLPP3,UGCG,TMEM245, NCBP1,APTX,TSN,DYNLL1,SNX11,HERPUD2,GPS2,PRPS2,ZDHHC20,CBX6,BRMS1,

				URGCP,PLEKHA8,PRNP,PPIP5K1,CAPZB,WEE1,ZDHHC17,EZH1,ZC3H18,VWA3B,ZBTB20,TTC26,PRRC1,TMEM33,PLRG1,SLC35A4,FAM92A,TSKU,HSPA8,TMEM258,TUBA1C,LDHA,ZNF362,TRIAP1,TGIF2LX,ARPP19,MYL12A,NUDT3,NOMO2,ZFAND6,PCGF2,CO-BLL1,ACTB,PNISR,MYO1C,MRTFA
BTO:0000081	Reproductive system	0.14	0.0028	RHBDD2,APPBP2,RRN3,CDC34,MYH9,AURKA,CDIPT,TNFAIP1,TUSC2,KALRN, SEC61A1,ALKBH7,MBD4,HBA2,TRPC4AP,KRAS,LAMTOR5,PNPLA8,MDM2,MIB1,PLD2, ASF1B,MED26,SLC9A1,EHBP1,TUBB4A,PRUNE1,RNF7,SIGMAR1,MYL9,CCNB2, MRPL17,RFX5,ARL5A,CCNL1,MON1A,VIRMA,DNAAF2,ZNF22,RPUSD4,PPIB,LDHAL6B,PGK2,DDX19A,EIF2AK3,PRSS1,RCE1,NARF,SLC5A6,AQP1,TNKS,CC2D1A,SPHK1,CD46, AHCYL2,MYEF2,MRFAP1,LYPLA1,MYO1D,STK11,TUBA3D,GLRX5,KDELR1, SPPL2C,EMC10,ATG101,UBE3B,SMARCB1,STAT5A,SYDE1,KLHL7,NBR1,ANP32B,DMPK, TTC30A,OSCP1,DYNLRB1,G3BP2,PHLDB1,MT-CO1,MAP3K3, GNPAT,SDHC,NUS1,ATP5PB,ATP5MD,SLC35A3,PLPP3,UGCG,TMEM245,NCBP1,APTX,TSN,DYNLL1,SNX11,HERPUD2,GPS2,PRPS2,ZDHHC20,CBX6,BRMS1,URGCP, PLEKHA8,PRNP,PPIP5K1,CAPZB,WEE1,ZDHHC17,EZH1,ZC3H18,VWA3B,ZBTB20,TTC26,PRRC1,TMEM33,PLRG1,SLC35A4,FAM92A,TSKU,HSPA8,TMEM258,TUBA1C,LDHA,ZNF362,TRIAP1,TGIF2LX,ARPP19,MYL12A,NUDT3,NOMO2,ZFAND6,PCGF2,CO-BLL1,ACTB,PNISR,MYO1C,MRTFA
BTO:0003091	Urogenital system	0.13	0.0032	RHBDD2,APPBP2,RRN3,CDC34,MYH9,AURKA,CDIPT,CHMP5,TNFAIP1,TUSC2, KALRN,SEC61A1,ALKBH7,MBD4,HBA2,TRPC4AP,KRAS,LAMTOR5,PNPLA8,MDM2,MIB1,TRAM1,PLD2,ASF1B,MED26,SLC9A1,EHBP1,TUBB4A,ICAM1,PRUNE1,RNF7,SIG-MAR1,MYL9,MED7,CCNB2,MRPL17,RFX5,ARL5A,CCNL1,MON1A,VIRMA,DNAAF2,ZNF22,RPUSD4,PPIB,LDHAL6B,PGK2,DDX19A,EIF2AK3,PRSS1,RCE1,NARF,SLC5A6,AQP1, TNKS,CC2D1A,SPHK1,CD46,AHCYL2,MYEF2,MRFAP1,LYPLA1,MYO1D,STK11, TUBA3D,GLRX5,KDELR1,SPPL2C,UQCR10,EMC10,ATG101,UBE3B,SMARCB1,STAT5A,SYDE1,KLHL7,NBR1,ANP32B,DMPK,TTC30A,OSCP1,DYNLRB1,G3BP2,PHLDB1,MT-

				CO1,MAP3K3,GNPAT,SDHC,NUS1,ATP5PB,ATP5MD,SLC35A3,PLPP3, UGCG, TMEM245,SLC39A7,NCBP1,APTX,TSN,DYNLL1,SNX11,HERPUD2,GPS2,PRPS2, ZDHHC20,CBX6,BRMS1,URGCP,PLEKHA8,PRNP,PPIP5K1,CAPZB,WEE1,ZDHHC17,EZH1,ZC3H18,VWA3B,ZBTB20,TTC26,PRRC1,TMEM33,LMF2,PLRG1,SLC35A4,FAM92A,TSKU,HSPA8,TMEM258,TUBA1C,LDHA,ZNF362,TRIAP1,TGIF2LX,ARPP19,MYL12A,TBC1D7,NUDT3,FRK,NOMO2,ZFAND6,PCGF2,COBLL1,ACTB,EYA1,PNISR,MYO1C,MRTFA
BTO:0001484	Nervous system	0.13	0.0236	RHBDD2,PPP2R5B,RRN3,CDC34,MYH9,ZFYVE21,CDIPT,CHMP5,TNFAIP1,TUSC2,PFN2, KALRN,SEC61A1,MBD4,HBA2,TRPC4AP,KRAS,APC,PNPLA8,MDM2,MIB1,TRAM1,MAST3,PLD2,EHBP1,TUBB4A,SEMA3A,DEF8,PRUNE1,SFXN5,PNKD,RNF7,SIGMAR1, CCNB2,CZIB,ARL5A,CCNL1,MON1A,VIRMA,POLR3E,PPIB,SPRYD3,DDX19A,EIF2AK3,LRRC7,KLF7,NARF,SLC5A6,GLS2,AQP1,CC2D1A,SSH1,AHCYL2,COX5A,MRFAP1,CAMK2G,LYPLA1,MYO1D,STK11,SMDT1,CNGA2,FLRT2,EMC10,UBE3B,SMARCB1,KLHL7, NBR1,ICMT,DMPK,PCNT,G3BP2,PHLDB1,MAP3K3,GNPAT,SDHC,AP4B1,ATP5PB,OMA1,PLPP3,ERI3,TRAF3IP1,NCBP1,UPF3A,CTNNBIP1,APTX,DNAJC19,TSN,DYNLL1,ATN1,SLC35E3,PRNP,PPIP5K1,CAPZB,WEE1,ZDHHC17,MRPL55,EZH1,NT5DC2,HRAS,ZC3H18, RPL7L1,BTBD9,PRRC1,PTDSS1,RNPC3,RBM4B,CCS,HSPA8,PACC1,GRAMD1A,RSPRY1,TUBA1C,LDHA,BRF1,DTX3,SMARCC2,ARPP19,POP4,NUDT3,TOX4,ZFAND6,ACTB,PNISR,SLC35B1,MRTFA
BTO:0000227	Central nervous system	0.13	0.0304	RHBDD2,PPP2R5B,RRN3,CDC34,MYH9,ZFYVE21,CDIPT,CHMP5,TNFAIP1,TUSC2,PFN2, KALRN,SEC61A1,MBD4,HBA2,TRPC4AP,KRAS,APC,PNPLA8,MDM2,MIB1,TRAM1,MAST3,PLD2,EHBP1,TUBB4A,SEMA3A,DEF8,PRUNE1,SFXN5,PNKD,RNF7,SIGMAR1, CCNB2,CZIB,ARL5A,CCNL1,MON1A,VIRMA,POLR3E,PPIB,SPRYD3,DDX19A,EIF2AK3,LRRC7,KLF7,NARF,SLC5A6,GLS2,AQP1,CC2D1A,AHCYL2,COX5A,MRFAP1,CAMK2G,MYO1D,STK11,SMDT1,FLRT2,EMC10,UBE3B,SMARCB1,KLHL7,NBR1,ICMT,DMPK,PCNT, G3BP2,PHLDB1,MAP3K3,GNPAT,SDHC,AP4B1,ATP5PB,OMA1,PLPP3,ERI3,NCBP1,UPF3A,CTNNBIP1,APTX,DNAJC19,TSN,DYNLL1,ATN1,SLC35E3,PRNP,PPIP5K1,CAPZB,WEE

				1,ZDHHC17,MRPL55,EZH1,NT5DC2,HRAS,ZC3H18,RPL7L1,BTBD9,PRRC1,PTDSS1,RNPC3,RBM4B,CCS,HSPA8,PACC1,GRAMD1A,RSPRY1,TUBA1C,LDHA,BRF1,DTX3,SMARCC2,ARPP19,POP4,NUDT3,TOX4,ZFAND6,ACTB,PNISR,SLC35B1,MRTFA
BTO:0000142	Brain	0.13	0.0335	RHBDD2,PPP2R5B,RRN3,CDC34,ZFYVE21,CDIPT,CHMP5,TNFAIP1,TUSC2,PFN2,KALRN,SEC61A1,MBD4,HBA2,TRPC4AP,KRAS,APC,PNPLA8,MDM2,MIB1,TRAM1,MAST3,PLD2,EHBP1,TUBB4A,SEMA3A,DEF8,PRUNE1,SFXN5,PNKD,RNF7,SIGMAR1,CCNB2,CZIB,ARL5A,CCNL1,MON1A,VIRMA,POLR3E,PPIB,SPRYD3,DDX19A,EIF2AK3,LRRC7,KLF7,NARF,SLC5A6,GLS2,AQP1,CC2D1A,AHCYL2,COX5A,MRFAP1,CAMK2G,MYO1D,STK11,SMDT1,FLRT2,EMC10,UBE3B,SMARCB1,KLHL7,NBR1,ICMT,DMPK,PCNT,G3BP2,PHLDB1,MAP3K3,GNPAT,SDHC,AP4B1,ATP5PB,OMA1,PLPP3,ERI3,NCBP1,UPF3A,CTNNBIP1,APTX,DNAJC19,TSN,DYNLL1,ATN1,SLC35E3,PRNP,PPIP5K1,CAPZB,WEE1,ZDHHC17,MRPL55,EZH1,NT5DC2,HRAS,ZC3H18,RPL7L1,BTBD9,PTDSS1,RNPC3,RBM4B,CCS,HSPA8,PACC1,GRAMD1A,RSPRY1,TUBA1C,LDHA,BRF1,DTX3,SMARCC2,ARPP19,POP4,NUDT3,TOX4,ZFAND6,ACTB,PNISR,SLC35B1,MRTFA
BTO:0000282	Head	0.11	0.0476	RHBDD2,PPP2R5B,RRN3,CDC34,ZFYVE21,CDIPT,CHMP5,TNFAIP1,GNB3,TUSC2,PFN2,KALRN,SEC61A1,MBD4,HBA2,TRPC4AP,KRAS,APC,PNPLA8,MDM2,KIF13A,MIB1,TRAM1,MAST3,PLD2,EHBP1,TUBB4A,SEMA3A,DEF8,PRUNE1,SFXN5,PNKD,RNF7,SIG-MAR1,CCNB2,CZIB,ARL5A,CCNL1,MON1A,VIRMA,POLR3E,PPIB,SPRYD3,SOX7,DDX19A,EIF2AK3,LRRC7,KLF7,NARF,SLC5A6,GLS2,AQP1,CC2D1A,CD46,SSH1,AHCYL2,COX5A,MRFAP1,CAMK2G,MYO1D,STK11,SMDT1,CNGA2,FLRT2,EMC10,SIRT6,UBE3B,SMARCB1,KLHL7,NBR1,ICMT,DMPK,PCNT,G3BP2,PHLDB1,MT-CO1,MAP3K3,GNPAT,SDHC,AP4B1,ATP5PB,OMA1,PLPP3,ERI3,TMEM245, NCBP1,UPF3A,NMNAT1,CTNNBIP1,APTX,DNAJC19,TSN,DYNLL1,GALNT3,ATN1,SLC35E3,PRNP,PPIP5K1,CAPZB,WEE1,ZDHHC17,MRPL55,EZH1,NT5DC2,HRAS,ZC3H18,RPL7L1,BTBD9,SLC35A4,PTDSS1,RNPC3,RBM4B,CCS,HSPA8,PACC1,GRAMD1A,RSPRY1,TUBA1C,LDHA,BRF1,DTX3,

				SMARCC2,ARPP19,POP4,DNAH11,NUDT3,TOX4,ZFAND6,ACTB,PNISR,MYO1C,SLC35B1,MRTFA
BTO:0001488	Endocrine gland	0.12	0.0476	RHBDD2,APPBP2,RRN3,MYH9,AURKA,CDIPT,CKM,HAS1,CHMP5,TUSC2,SEC61A1, ALKBH7,MBD4,HBA2,TRPC4AP,TERF2,KRAS,LAMTOR5,APC,MDM2,TRAM1,IT-PKC,ASF1B,PNO1,EHBP1,TUBB4A,ICAM1,DEF8,PRUNE1,PNKD,SIGMAR1,ZFP36L2, ITGA1,CCNB2,MRPL17,RFX5,ARL5A,CCNL1,MICALL2,VIRMA,POLR3E,PPIB,LDHAL6B, KLF13,PGK2,EIF2AK3,PRSS1,RCE1,GLS2,TNKS,SPHK1,CD46,MYEF2,COX5A,MRFAP1,CAMK2G,DEF6,LYPLA1,MYO1D,STK11,TUBA3D,GLRX5,SPPL2C,UQCR10,EMC10,SIRT6, PPL,UBE3B,KLHL7,NBR1,ICMT,ANP32B,DMPK,TTC30A,OSCP1,DYNLRB1,LAGE3,G3BP2,MT-CO1,GNPAT,SDHC,ATP5PB,ATP5MD,GCLM,PKIG, UGCG,TMEM245,SLC39A7, NCBP1,APTX,CENPJ,DYNLL1,TRAF3,SNX11,HERPUD2,GPS2,PRPS2,ZDHHC20, PLEKHG2,BRMS1,PRNP,CAPZB,WEE1,EZH1,ZC3H18,VWA3B,ZBTB20,TTC26,PRRC1,LMF2,SLC35A4,FAM92A,CCS,HSPA8,TUBA1C,LDHA,ATXN2,SMARCC2,TGIF2LX,MYL12A, CHMP2A,NOMO2,TOX4,ZFAND6,COBLL1,ACTB,PNISR,MYO1C,MRTFA
GOCC:0005622	Intracellular	0.13	1.01E-11	RHBDD2,APPBP2,PPP2R5B,RRN3,CDC34,TCN2,MYH9,ZFYVE21,AURKA,RP2, CDIPT,CKM,HAS1,CHMP5,TNFAIP1,GNB3,PFN2,KALRN,SEC61A1,ALKBH7,NSRP1,YEATS4,MBD4,HBA2,TRPC4AP,MRPL34,LSM7,TERF2,KRAS,LAMTOR5,APC,PNPLA8,MDM2,BRIP1,KIF13A,GCHFR,MIB1,TRAM1,PLD2,ITPKC,ASF1B,MED26,PNO1,SLC9A1,EHBP1, TUBB4A,POLQ,ALG10,PRUNE1,SFXN5,PNKD,RNF7,SIGMAR1,MYL9,ZFP36L2,MED7, RASA2,MTERF3,CCNB2,MRPL17,MITD1,RFX5,CCNL1,MON1A,MICALL2,VIRMA, DNAAF2,ZNF22,RPUSD4,POLR3E,PPIB,SOX7,LDHAL6B,KLF13,PGK2,EIF2AK3,MYL1,RCE1,LRRC7,KLF7,NARF,GLS2,AQP1,ACOT1,TNKS,CC2D1A,SPHK1,CD46,SSH1,ZNF609,MYEF2,COX5A,MRFAP1,CAMK2G,DEF6,LYPLA1,MYO1D,STK11,TUBA3D,TMEM39A, ZBTB11,SMDT1,CNGA2,GLRX5,KDELR1,SPPL2C,UQCR10,EMC10,SIRT6,ATG101,CIR1,PPL,SMARCB1,STAT5A,KLHL7,NBR1,ICMT,ANP32B,DMPK,TTC30A,OSCP1,DYNLRB1,LAGE3,NLRC3,PCNT,G3BP2,PHLDB1,MT-CO1,MAP3K3,ZNF318,MT-CO3,TSEN15,

				GNPAT,SDHC,NUS1,AP4B1,ATP5PB,ATP5MD,ARMH3,SLC35A3,GCLM,OMA1,PLPP3,FRA10AC1,DDX31,CHCHD1,TOR2A,TRAF3IP1,UGCG,CSGALNACT2,SLC39A7,TAP2,NCBP1,UPF3A,STK19,NMNAT1,CTNNBIP1,KIN,APTX,CENPJ,DNAJC19,MRPL2,TSN,DYNLL1,GALNT3,TRAF3,SNX11,GPS2,ATN1,DOP1B,ZDHHC20,CBX6,ZBED8,TONSL,TMEM131L,MTMR11,PLEKHG2,BRMS1,URGCP,PLEKHA8,PRNP,PPIP5K1,CAPZB,HECTD2,WEE1,TMEM177,ZDHHC17,MRPL55,EZH1,NDUFB3,HRAS,SS18,CBFB,ZC3H18,YIPF6,VWA3B,ZBTB20,SMYD3,TMEM33,PLRG1,FAM92A,INTS8,PTDSS1,JAK3,RNPC3,RBM4B,CCS,HSPA8,PHC1,GRAMD1A,TMEM258,TUBA1C,LDHA,ATXN2,BRF1,SMARCC2,TRIAP1,ARPP19,MYL12A,POP4,UQCR11,CHMP2A,SETD1B,TBC1D7,DNAH11,NUDT3,FRK,NOMO2,TOX4,SLC25A14,ZFAND6,IRAK4,PCGF2,LEPROT,ACTB,EYA1,PNISR,MYO1C,SLC35B1,MRTFA,MED19,NIF3L1
GOCC:0043226	Organelle	0.15	1.01E-11	RHBDD2,APPBP2,RRN3,CDC34,TCN2,MYH9,ZFYVE21,AURKA,RP2,CDIPT,CKM,HAS1,CHMP5,TNFAIP1,PFN2,KALRN,SEC61A1,ALKBH7,NSRP1,YEATS4,MBD4,HBA2,MRPL34,LSM7,TERF2,KRAS,LAMTOR5,APC,PNPLA8,MDM2,BRIP1,KIF13A,GCHFR,MIB1,TRAM1,PLD2,ITPKC,ASF1B,MED26,PNO1,SLC9A1,EHBP1,TUBB4A,POLQ,ICAM1,ALG10,PRUNE1,SFXN5,PNKD,RNF7,SIGMAR1,MYL9,ZFP36L2,ITGA1,MED7,MTERF3,CCNB2,MRPL17,MITD1,RFX5,CCNL1,MON1A,MICALL2,VIRMA,DNAAF2,ZNF22,RPUSD4,POLR3E,PPIB,SOX7,LDHAL6B,KLF13,PGK2,EIF2AK3,MYL1,RCE1,LRRC7,KLF7,NARF,GLS2,AQP1,TNKS,CC2D1A,SPHK1,CD46,SSH1,ZNF609,MYEF2,COX5A,CAMK2G,LY-PLA1,MYO1D,STK11,TUBA3D,TMEM39A,ZBTB11,SMDT1,CNGA2,GLRX5,KDELR1,SPPL2C,UQCR10,EMC10,SIRT6,ATG101,CIR1,PPL,SMARCB1,STAT5A,KLHL7,NBR1,ICMT,ANP32B,DMPK,TTC30A,DYNLRB1,LAGE3,NLRC3,PCNT,G3BP2,PHLDB1,MT-CO1,ZNF318,MT-CO3,TSEN15,GNPAT,SDHC,NUS1,AP4B1,ATP5PB,ATP5MD,ARMH3,SLC35A3,OMA1,PLPP3,DDX31,CHCHD1,TOR2A,TRAF3IP1,UGCG,CSGALNACT2,SLC39A7,TAP2,NCBP1,UPF3A,NMNAT1,CTNNBIP1,KIN,APTX,CENPJ,DNAJC19,MRPL2,DYNLL1,GALNT3,TRAF3,SNX11,GPS2,ATN1,DOP1B,ZDHHC20,CBX6,TONSL,MTMR11,

				BRMS1,PLEKHA8,PRNP,CAPZB,WEE1,TMEM177,ZDHHC17,MRPL55,EZH1,NDUFB3,HRAS,SS18,CBFB,ZC3H18,YIPF6,ZBTB20,SMYD3,TTC26,TMEM33,PLRG1,FAM92A,INTS8,PTDSS1,JAK3,RNPC3,RBM4B,CCS,HSPA8,PHC1,GRAMD1A,TMEM258,TUBA1C,LDHA,ATXN2,BRF1,SMARCC2,TRIAP1,MYL12A,POP4,UQCR11,CHMP2A,SETD1B,TBC1D7,DNAH11,FRK,NOMO2,TOX4,SLC25A14,IRAK4,PCGF2,LEPROT,COBLL1,ACTB,EYA1, PNISR,MYO1C,SLC35B1,MRTFA,MED19,NIF3L1
GOCC:0043229	Intracellular organelle	0.16	1.01E-11	RHBDD2,APPBP2,RRN3,CDC34,TCN2,MYH9,ZFYVE21,AURKA,RP2,CDIPT,CKM, HAS1,CHMP5,TNFAIP1,PFN2,KALRN,SEC61A1,ALKBH7,NSRP1,YEATS4,MBD4,HBA2,MRPL34,LSM7,TERF2,KRAS,LAMTOR5,APC,PNPLA8,MDM2,BRIP1,KIF13A,GCHFR,MIB1, TRAM1,PLD2,ITPKC,ASF1B,MED26,PNO1,SLC9A1,EHBP1,TUBB4A,POLQ,ALG10, PRUNE1,SFXN5,PNKD,RNF7,SIGMAR1,MYL9,ZFP36L2,MED7,MTERF3,CCNB2,MRPL17, MITD1,RFX5,CCNL1,MON1A,MICALL2,VIRMA,DNAAF2,ZNF22,RPUSD4,POLR3E, PPIB,SOX7,LDHAL6B,KLF13,PGK2,EIF2AK3,MYL1,RCE1,LRRC7,KLF7,NARF,GLS2,AQP1,TNKS,CC2D1A,SPHK1,CD46,SSH1,ZNF609,MYEF2,COX5A,CAMK2G,LYPLA1, MYO1D,STK11,TUBA3D,TMEM39A,ZBTB11,SMDT1,CNGA2,GLRX5,KDELR1,SPPL2C,UQCR10,EMC10,SIRT6,ATG101,CIR1,PPL,SMARCB1,STAT5A,KLHL7,NBR1,ICMT,ANP32B,DMPK,TTC30A,DYNLRB1,LAGE3,NLRC3,PCNT,G3BP2,PHLDB1,MT-CO1,ZNF318, MT-CO3,TSEN15,GNPAT,SDHC,NUS1,AP4B1,ATP5PB,ATP5MD,ARMH3,SLC35A3,OMA1, PLPP3,DDX31,CHCHD1,TOR2A,UGCG,CSGALNACT2,SLC39A7,TAP2,NCBP1,UPF3A, NMNAT1,CTNNBIP1,KIN,APTX,CENPJ,DNAJC19,MRPL2,DYNLL1,GALNT3,TRAF3,SNX11,GPS2,ATN1,DOP1B,ZDHHC20,CBX6,TONSL,BRMS1,PLEKHA8,PRNP,CAPZB,WEE1,TMEM177,ZDHHC17,MRPL55,EZH1,NDUFB3,HRAS,SS18,CBFB,ZC3H18,YIPF6,ZBTB20,SMYD3,TMEM33,PLRG1,FAM92A,INTS8,PTDSS1,JAK3,RNPC3,RBM4B,CCS,HSPA8,PHC1, GRAMD1A,TMEM258,TUBA1C,LDHA,ATXN2,BRF1,SMARCC2,TRIAP1,MYL12A,POP4,UQCR11,CHMP2A,SETD1B,TBC1D7,DNAH11,FRK,NOMO2,TOX4,SLC25A14,IRAK4,PCGF2,LEPROT,ACTB,EYA1,PNISR,MYO1C,SLC35B1,MRTFA,MED19

GOCC:0043231	Intracellular membrane-bounded organelle	0.18	4.32E-11	RHBDD2,APPBP2,RRN3,CDC34,TCN2,MYH9,ZFYVE21,AURKA,CDIPT,CKM,HAS1,CHMP5,TNFAIP1,SEC61A1,ALKBH7,NSRP1,YEATS4,MBD4,MRPL34,LSM7,TERF2,KRAS,LAMTOR5,APC,PNPLA8,MDM2,BRIP1,KIF13A,GCHFR,TRAM1,PLD2,ITPKC,ASF1B,MED26,PNO1,POLQ,ALG10,PRUNE1,SFXN5,PNKD,RNF7,SIGMAR1,ZFP36L2,MED7,MTERF3,CCNB2,MRPL17,MITD1,RFX5,CCNL1,MON1A,MICALL2,VIRMA,ZNF22,RPUSD4,POLR3E,PPIB,SOX7,LDHAL6B,KLF13,PGK2,EIF2AK3,RCE1,LRRC7,KLF7,NARF,GLS2,AQP1,TNKS,CC2D1A,SPHK1,ZNF609,MYEF2,COX5A,CAMK2G,LYPLA1,MYO1D,STK11,TMEM39A,ZBTB11,SMDT1,GLRX5,KDELR1,SPPL2C,UQCR10,EMC10,SIRT6,ATG101,CIR1,SMARCB1,STAT5A,KLHL7,NBR1,ICMT,ANP32B,DMPK,LAGE3,PHLDB1,MT-CO1,ZNF318,MT-CO3,TSEN15,GNPAT,SDHC,NUS1,AP4B1,ATP5PB, ATP5MD,ARMH3,SLC35A3,OMA1,PLPP3,DDX31,CHCHD1,TOR2A,UGCG,CSGALNACT2,SLC39A7,TAP2,NCBP1,UPF3A,NMNAT1,CTNNBIP1,KIN,APTX,DNAJC19,MRPL2,DYNLL1,GALNT3,TRAF3,SNX11,GPS2,ATN1,DOP1B,ZDHHC20,CBX6,TONSL,BRMS1,PLEKHA8,PRNP,WEE1,TMEM177,ZDHHC17,MRPL55,EZH1,NDUFB3,HRAS,SS18,CBFB,ZC3H18,YIPF6,ZBTB20,SMYD3,TMEM33,PLRG1,FAM92A,INTS8,PTDSS1,JAK3,RNPC3,RBM4B,CCS,HSPA8,PHC1,GRAMD1A,TMEM258,TUBA1C,LDHA,ATXN2,BRF1,SMARCC2,TRIAP1,POP4,UQCR11,CHMP2A,SETD1B,TBC1D7,FRK,NOMO2,TOX4,SLC25A14,IRAK4,PCGF2,LEPROT,ACTB,EYA1,PNISR,MYO1C,SLC35B1,MRTFA,MED19
GOCC:0043227	Membrane-bounded organelle	0.16	9.03E-11	RHBDD2,APPBP2,RRN3,CDC34,TCN2,MYH9,ZFYVE21,AURKA,RP2,CDIPT,CKM,HAS1,CHMP5,TNFAIP1,SEC61A1,ALKBH7,NSRP1,YEATS4,MBD4,HBA2,MRPL34,LSM7,TERF2,KRAS,LAMTOR5,APC,PNPLA8,MDM2,BRIP1,KIF13A,GCHFR,TRAM1,PLD2,ITPKC,ASF1B,MED26,PNO1,TUBB4A,POLQ,ICAM1,ALG10,PRUNE1,SFXN5,PNKD,RNF7,SIGMAR1,ZFP36L2,ITGA1,MED7,MTERF3,CCNB2,MRPL17,MITD1,RFX5,CCNL1,MON1A,MICALL2,VIRMA,ZNF22,RPUSD4,POLR3E,PPIB,SOX7,LDHAL6B,KLF13,PGK2,EIF2AK3,RCE1,LRRC7,KLF7,NARF,GLS2,AQP1,TNKS,CC2D1A,SPHK1,CD46,ZNF609,MYEF2,COX5A,CAMK2G,LYPLA1,MYO1D,STK11,TMEM39A,ZBTB11,SMDT1,CNGA2,

				GLRX5,KDELR1,SPPL2C,UQCR10,EMC10,SIRT6,ATG101,CIR1,SMARCB1,STAT5A,KLHL7,NBR1,ICMT,ANP32B,DMPK,LAGE3,PHLDB1,MT-CO1,ZNF318,MT-CO3,TSEN15,GNPAT,SDHC,NUS1,AP4B1,ATP5PB,ATP5MD,ARMH3,SLC35A3,OMA1,PLPP3,DDX31,CHCHD1,TOR2A,TRAF3IP1,UGCG,CSGALNACT2,SLC39A7,TAP2,NCBP1,UPF3A,NMNAT1,CTNNBIP1,KIN,APTX,DNAJC19,MRPL2,DYNLL1,GALNT3,TRAF3,SNX11,GPS2,ATN1,DOP1B,ZDHHC20,CBX6,TONSL,BRMS1,PLEKHA8,PRNP,CAPZB,WEE1,TMEM177,ZDHHC17,MRPL55,EZH1,NDUFB3,HRAS,SS18,CBFB,ZC3H18,YIPF6,ZBTB20,SMYD3,TTC26,TMEM33,PLRG1,FAM92A,INTS8,PTDSS1,JAK3,RNPC3,RBM4B,CCS,HSPA8,PHC1,GRAMD1A,TMEM258,TUBA1C,LDHA,ATXN2,BRF1,SMARCC2,TRIAP1,POP4,UQCR11,CHMP2A,SETD1B,TBC1D7,DNAH11,FRK,NOMO2,TOX4,SLC25A14,IRAK4,PCGF2,LEP-ROT,COBLL1,ACTB,EYA1,PNISR,MYO1C,SLC35B1,MRTFA,MED19,NIF3L1
GOCC:0031090	Organelle membrane	0.32	2.90E-07	RHBDD2,APPBP2,CDIPT,CHMP5,SEC61A1,KRAS,LAMTOR5,PNPLA8,MDM2,BRIP1,KIF13A,GCHFR,TRAM1,ALG10,MTERF3,MITD1,EIF2AK3,RCE1,AQP1,TNKS,SPHK1,CD46,COX5A,CAMK2G,LYPLA1,TMEM39A,SMDT1,CNGA2,KDELR1,SPPL2C,UQCR10,EMC10,ATG101,NBR1,ICMT,DMPK,MT-CO1,MT-CO3,GNPAT,SDHC,NUS1,AP4B1,ATP5PB,ATP5MD,ARMH3, SLC35A3,OMA1,PLPP3,UGCG,CSGALNACT2,TAP2,DNAJC19,DYNLL1,GALNT3,DOP1B,ZDHHC20,PLEKHA8,PRNP,TMEM177,ZDHHC17,MRPL55,NDUFB3,HRAS,TMEM33,FAM92A,HSPA8,GRAMD1A,TMEM258,UQCR11,CHMP2A,NOMO2,SLC25A14,IRAK4,SLC35B1
GOCC:0005737	Cytoplasm	0.14	3.36E-06	RHBDD2,APPBP2,CDC34,TCN2,MYH9,ZFYVE21,AURKA,RP2,CDIPT,CKM,HAS1,CHMP5,TNFAIP1,GNB3,KALRN,SEC61A1,ALKBH7,HBA2,MRPL34,KRAS,LAMTOR5,APC,PNPLA8,MDM2,BRIP1,KIF13A,GCHFR,TRAM1,PLD2,ITPKC,PNO1,SLC9A1,EHBP1,TUBB4A,POLQ,ALG10,PRUNE1,SFXN5,PNKD,SIGMAR1,MYL9,RASA2,MTERF3,CCNB2,MRPL17,MITD1,MON1A,MICALL2,VIRMA,DNAAF2,RPUSD4,PPIB,SOX7,LDHAL6B,PGK2,EIF2AK3,MYL1,RCE1,LRRC7,GLS2,AQP1,ACOT1,TNKS,SPHK1,CD46,SSH1,COX5A,CAMK2G,DEF6,LYPLA1,MYO1D,STK11,TMEM39A,SMDT1,CNGA2,

				GLRX5,KDELR1,SPPL2C,UQCR10,EMC10,SIRT6,ATG101,STAT5A,KLHL7,NBR1,ICMT,ANP32B,DMPK,TTC30A,OSCP1,DYNLRB1,LAGE3,NLRC3,PCNT,G3BP2,PHLDB1,MT-CO1,MAP3K3,ZNF318,MT-CO3,GNPAT,SDHC,NUS1,AP4B1,ATP5PB,ATP5MD,ARMH3,SLC35A3,GCLM,OMA1,PLPP3,CHCHD1,TOR2A,UGCG,CSGALNACT2,SLC39A7,TAP2,NCBP1,CTNNBIP1,CENPJ,DNAJC19,MRPL2,TSN,DYNLL1,GALNT3,TRAF3,SNX11,GPS2,DOP1B,ZDHHC20,TMEM131L,MTMR11,PLEKHG2,BRMS1,URGCP,PLEKHA8,PRNP,PPIP5K1,CAPZB,WEE1,TMEM177,ZDHHC17,MRPL55,NDUFB3,HRAS,YIPF6,SMYD3,TMEM33,FAM92A,PTDSS1,JAK3,CCS,HSPA8,GRAMD1A,TMEM258,LDHA,ATXN2,TRIAP1,MYL12A,UQCR11,CHMP2A,TBC1D7,DNAH11,FRK,NOMO2,SLC25A14,ZFAND6,IRAK4,LEPROT,ACTB,EYA1,PNISR,MYO1C,SLC35B1,MRTFA,NIF3L1
GOCC:0032991	Protein-containing complex	0.19	7.72E-06	RHBDD2,APPBP2,PPP2R5B,MYH9,AURKA,CKM,CHMP5,TNFAIP1,GNB3,SEC61A1,NSRP1,YEATS4,HBA2,TRPC4AP,MRPL34,LSM7,TERF2,LAMTOR5,APC,MDM2,BRIP1,KIF13A,GCHFR,ASF1B,MED26,SLC9A1,EHBP1,ICAM1,PRUNE1,RNF7,MYL9,ITGA1,MED7,CCNB2,MRPL17,MON1A,VIRMA,POLR3E,EIF2AK3,MYL1,UTP23,RCE1,GLS2,TNKS,COX5A,CAMK2G,MYO1D,STK11,SMDT1,CNGA2,GLRX5,KDELR1,UQCR10,EMC10,ATG101,CIR1,SMARCB1,KLHL7,NBR1,TTC30A,DYNLRB1,LAGE3,PCNT,MT-CO1,MT-CO3,TSEN15,SDHC,NUS1,AP4B1,ATP5PB, ATP5MD,SLC35A3,GCLM,OMA1,FRA10AC1,TRAF3IP1,TAP2,NCBP1,UPF3A,NMNAT1,CTNNBIP1,KIN,CENPJ,DNAJC19,MRPL2,DYNLL1,TRAF3,GPS2,PRPS2,CBX6,BRMS1,PRNP,CAPZB,MRPL55,EZH1,NDUFB3,HRAS,SS18,CBFB,ZC3H18,TTC26,PLRG1,PRPS1L1,INTS8,RNPC3,RBM4B,HSPA8,PHC1,TMEM258,LDHA,ATXN2,BRF1,SMARCC2,TRIAP1,MYL12A,POP4,UQCR11,CHMP2A,SETD1B,TBC1D7,DNAH11,NOMO2,TOX4,PCGF2,ACTB,MYO1C,MED19
GOCC:1902494	Catalytic complex	0.31	0.00013	RHBDD2,APPBP2,PPP2R5B,CKM,TNFAIP1,GNB3,YEATS4,TRPC4AP,APC,GCHFR,PRUNE1,RNF7,CCNB2,VIRMA,POLR3E,EIF2AK3,RCE1,GLS2,STK11,UQCR10,ATG101,SMARCB1,KLHL7,DYNLRB1,MT-CO1,TSEN15,SDHC,NUS1,SLC35A3,GCLM,OMA1,NMNAT1,DYNLL1,TRAF3, PRPS2,BRMS1,EZH1,NDUFB3,HRAS,SS18,PLRG1,

				PRPS1L1,PHC1,TMEM258,LDHA,SMARCC2,POP4,UQCR11,SETD1B,DNAH11,TOX4,PCGF2,ACTB,MYO1C
GOCC:0031975	Envelope	0.39	0.00099	CHMP5,KRAS,BRIP1,GCHFR,SIGMAR1,MTERF3,AQP1,TNKS,COX5A,LYPLA1,SMDT1, UQCR10,DMPK,MT-CO1,MT-CO3,SDHC,ATP5PB,ATP5MD,OMA1,DNAJC19,GPS2, PRNP,TMEM177, MRPL55,NDUFB3,TMEM33,FAM92A,TRIAP1,UQCR11,CHMP2A, SLC25A14,MYO1C
GOCC:0005789	Endoplasmic reticulum membrane	0.46	0.0012	RHBDD2,CDIPT,SEC61A1,KRAS,TRAM1,ALG10,EIF2AK3,RCE1,CAMK2G,TMEM39A,KDELR1,SPPL2C,EMC10,ATG101,ICMT,DMPK,NUS1,TAP2,HRAS,TMEM33,GRAMD1A,TMEM258,NOMO2,SLC35B1
GOCC:0031967	Organelle envelope	0.38	0.0017	CHMP5,KRAS,BRIP1,GCHFR,SIGMAR1,MTERF3,AQP1,TNKS,COX5A,LYPLA1,SMDT1, UQCR10,DMPK,MT-CO1,MT-CO3,SDHC,ATP5PB,ATP5MD,OMA1,DNAJC19,PRNP, TMEM177, MRPL55,NDUFB3,TMEM33,FAM92A,TRIAP1,UQCR11,CHMP2A, SLC25A14,MYO1C
GOCC:0005634	Nucleus	0.15	0.0075	APPBP2,RRN3,CDC34,MYH9,AURKA,CKM,CHMP5,TNFAIP1,NSRP1,YEATS4, MBD4,LSM7,TERF2,APC,MDM2,BRIP1,GCHFR,ITPKC,ASF1B,MED26,PNO1, POLQ,PRUNE1,PNKD,RNF7,SIGMAR1,ZFP36L2,MED7,CCNB2,RFX5,CCNL1, VIRMA,ZNF22,POLR3E,PPIB,SOX7,KLF13,KLF7,NARF,AQP1,TNKS,CC2D1A,ZNF609,MYEF2,CAMK2G,LYPLA1,STK11,ZBTB11,SIRT6,CIR1,SMARCB1,STAT5A,KLHL7, NBR1,ANP32B,DMPK,LAGE3,ZNF318,TSEN15,DDX31,CHCHD1,NCBP1,UPF3A,NMNAT1,KIN,APTX,DYNLL1,GPS2,ATN1,CBX6,TONSL,BRMS1,PRNP,WEE1,EZH1,HRAS,SS18,CBFB,ZC3H18,ZBTB20,SMYD3,TMEM33,PLRG1,FAM92A,INTS8,RNPC3,RBM4B,CCS,HSPA8,PHC1,TUBA1C,LDHA,BRF1,SMARCC2,POP4,CHMP2A,SETD1B,TOX4,PCGF2,ACTB,EYA1,PNISR,MYO1C,MRTFA,MED19

GOCC:0005739	Mitochondrion	0.31	0.0075	TCN2,ALKBH7,MRPL34,KRAS,PNPLA8,SFXN5,PNKD,MTERF3,MRPL17,RPUSD4,LDHAL6B,GLS2,COX5A,STK11,SMDT1,GLRX5,UQCR10,DMPK,MT-CO1,MT-CO3,SDHC, ATP5PB, ATP5MD,OMA1,CHCHD1,NCBP1,DNAJC19,MRPL2,GPS2,PRNP, TMEM177,MRPL55,NDUFB3,FAM92A,TRIAP1,UQCR11,SLC25A14
GOCC:0031984	Organelle sub-compartment	0.37	0.0075	CDIPT,SEC61A1,KRAS,KIF13A,TRAM1,ALG10,EIF2AK3,RCE1,TMEM39A,KDELR1,SPPL2C,EMC10,ATG101,ICMT,NUS1,AP4B1,PLPP3,TAP2,PLEKHA8,HRAS,YIPF6,TMEM33,GRAMD1A,TMEM258,ATXN2,NOMO2,SLC35B1
GOCC:0098800	Inner mitochondrial membrane protein complex	0.66	0.0075	COX5A,SMDT1,UQCR10,MT-CO1,SDHC,ATP5PB,ATP5MD,OMA1,DNAJC19,NDUFB3, UQCR11
GOCC:0098827	Endoplasmic reticulum subcompartment	0.43	0.0075	CDIPT,SEC61A1,KRAS,TRAM1,ALG10,EIF2AK3,RCE1,TMEM39A,KDELR1,SPPL2C,EMC10,ATG101,ICMT,NUS1,TAP2,HRAS,TMEM33,GRAMD1A,TMEM258,NOMO2,SLC35B1
GOCC:0110165	Cellular anatomical entity	0.06	0.0075	RHBDD2,APPBP2,RRN3,CDC34,TCN2,MYH9,ZFYVE21,AURKA,RP2,CDIPT,CKM, HAS1,CHMP5,TNFAIP1,GNB3,PFN2,KALRN,SEC61A1,ALKBH7,NSRP1,YEATS4,MBD4,HBA2,MRPL34,LSM7,TERF2,KRAS,LAMTOR5,APC,PNPLA8,MDM2,BRIP1,KIF13A,GCHFR, MIB1,TRAM1,PLD2,ITPKC,ASF1B,MED26,PNO1,SLC9A1,EHBP1,TUBB4A,POLQ,ICAM1, SEMA3A,ALG10,PRUNE1,SFXN5,PNKD,RNF7,SIGMAR1,MYL9,ZFP36L2,ITGA1,MED7, RASA2,MTERF3,CCNB2,MRPL17,MITD1,RFX5,CCNL1,MON1A,MICALL2,VIRMA, DNAAF2,ZNF22,RPUSD4,POLR3E,PPIB,SOX7,LDHAL6B,KLF13,PGK2,EIF2AK3,MYL1,PRSS1,RCE1,LRRC7,KLF7,NARF,SLC5A6,GLS2,AQP1,ACOT1,TNKS,CC2D1A,SPHK1,CD46,SSH1,ZNF609,MYEF2,COX5A,CAMK2G,DEF6,LYPLA1,MYO1D,STK11,TUBA3D, TMEM39A,ZBTB11,SMDT1,CNGA2,GLRX5,KDELR1,SPPL2C,FLRT2,UQCR10,EMC10,SIRT6,ATG101,CIR1,PPL,SMARCB1,STAT5A,KLHL7,NBR1,ICMT,ANP32B,DMPK,TTC30A,OSCP1,DYNLRB1,LAGE3,NLRC3,PCNT,G3BP2,PHLDB1,MT-CO1,MAP3K3,ZNF318, MT-

				CO3,TSEN15,GNPAT,SDHC,NUS1,AP4B1,ATP5PB,ATP5MD,ARMH3,SLC35A3, GCLM,OMA1,PLPP3,DDX31,DOLPP1,CHCHD1,TOR2A,TRAF3IP1,UGCG,CSGAL-NACT2,TMEM245,SLC39A7,TAP2,NCBP1,UPF3A,NMNAT1,CTNNBIP1,KIN,APTX,CENPJ ,DNAJC19,MRPL2,TSN,DYNLL1,GALNT3,TRAF3,SNX11,GPS2,ATN1,DOP1B,ZDHHC20, CBX6,TONSL,TMEM131L,MTMR11,PLEKHG2,BRMS1,URGCP,PLEKHA8,PRNP,PPIP5K1, CAPZB,WEE1,TMEM177,ZDHHC17,MRPL55,EZH1,NDUFB3,HRAS,SS18,CBFB,ZC3H18,Y IPF6,ZBTB20,SMYD3,TTC26,IQCJ-SCHIP1,TMEM33,LMF2,PLRG1,FAM92A,INTS8, PTDSS1,JAK3,RNPC3,RBM4B,TSKU,CCS,HSPA8,PHC1,PACC1,GRAMD1A,TMEM258,TUB A1C,LDHA,ATXN2,BRF1,DTX3,SMARCC2,TRIAP1,MYL12A,POP4,UQCR11,CHMP2A,SE TD1B,TBC1D7,DNAH11,FRK,NOMO2,TOX4,SLC25A14,ZFAND6,IRAK4,PCGF2,LEP-ROT,COBLL1,ACTB,EYA1,PNISR,MYO1C,SLC35B1,MRTFA,MED19,NIF3L1
GOCC:0 016459	Myosin complex	0.87	0.0088	MYH9,MYL9,MYL1,MYO1D,KDELR1,MYL12A,MYO1C
GOCC:0 005740	Mitochondrial envelope	0.42	0.0095	KRAS,MTERF3,COX5A,SMDT1,UQCR10,DMPK,MT-CO1,MT-CO3,SDHC,ATP5PB, ATP5MD, OMA1,DNAJC19,PRNP,TMEM177,MRPL55,NDUFB3,FAM92A,TRIAP1, UQCR11,SLC25A14
GOCC:0 031966	Mitochondrial membrane	0.43	0.0107	KRAS,MTERF3,COX5A,SMDT1,UQCR10,DMPK,MT-CO1,MT-CO3,SDHC,ATP5PB, ATP5MD,OMA1,DNAJC19,PRNP,TMEM177,MRPL55,NDUFB3,FAM92A,UQCR11, SLC25A14
GOCC:0 005743	Mitochondrial inner membrane	0.49	0.0109	COX5A,SMDT1,UQCR10,MT-CO1,MT-CO3,SDHC,ATP5PB,ATP5MD,OMA1,DNAJC19, TMEM177,MRPL55,NDUFB3,FAM92A,UQCR11,SLC25A14
GOCC:0 005783	Endoplasmic reticulum	0.3	0.011	RHBDD2,CDIPT,SEC61A1,KRAS,TRAM1,ALG10,SIGMAR1,PPIB,EIF2AK3,RCE1, CAMK2G,LYPLA1,MYO1D,TMEM39A,KDELR1,SPPL2C,EMC10,SIRT6,ATG101,ICMT,

				DMPK,NUS1,PLPP3,TOR2A,SLC39A7,TAP2,PRNP,HRAS,TMEM33,PTDSS1,GRAMD1A,TMEM258,NOMO2,SLC35B1
GOCC:0098798	Mitochondrial protein complex	0.5	0.0125	MRPL34,MRPL17,COX5A,SMDT1,UQCR10,MT-CO1,SDHC,ATP5PB,ATP5MD,OMA1,DNAJC19,MRPL2,MRPL55,NDUFB3,UQCR11
GOCC:0031300	Intrinsic component of organelle membrane	0.51	0.0149	SEC61A1,TRAM1,EIF2AK3,SMDT1,SPPL2C,EMC10,DMPK,OMA1,CSGALNACT2,TAP2,ZDHHC20,TMEM177,TMEM33,GRAMD1A
GOCC:0031301	Integral component of organelle membrane	0.52	0.0227	SEC61A1,TRAM1,EIF2AK3,SMDT1,SPPL2C,EMC10,DMPK,OMA1,CSGALNACT2,TAP2,ZDHHC20,TMEM177,TMEM33
GOCC:0012505	Endomembrane system	0.17	0.0316	RHBDD2,MYH9,ZFYVE21,CDIPT,HAS1,CHMP5,TNFAIP1,SEC61A1,KRAS,LAMTOR5,BRIP1,KIF13A,GCHFR,TRAM1,ALG10,SIGMAR1,MITD1,MON1A,MICALL2,PPIB,EIF2AK3,RCE1,LRRC7,AQP1,TNKS,SPHK1,CD46,CAMK2G,LYPLA1,MYO1D,TMEM39A,KDELR1,SPPL2C,EMC10,SIRT6,ATG101,NBR1,ICMT,DMPK,NUS1,AP4B1,ARMH3,SLC35A3,PLPP3,TOR2A,UGCG,CSGALNACT2,SLC39A7,TAP2,DYNLL1,GALNT3,SNX11,DOP1B,ZDHHC20,PLEKHA8,PRNP,ZDHHC17,HRAS,YIPF6,TMEM33,PTDSS1,HSPA8,GRAMD1A,TMEM258,ATXN2,CHMP2A,FRK,NOMO2,IRAK4,LEPROT,MYO1C,SLC35B1
GOCC:1990234	Transferase complex	0.31	0.0316	RHBDD2,APPBP2,CKM,TNFAIP1,YEATS4,TRPC4AP,PRUNE1,RNF7,CCNB2,VIRMA,POLR3E,EIF2AK3,RCE1,STK11,ATG101,KLHL7,NUS1,NMNAT1,TRAF3,PRPS2,EZH1,PRPS1L1,PHC1,TMEM258,LDHA,SETD1B,PCGF2,ACTB
GOCC:0070069	Cytochrome complex	0.93	0.0349	COX5A,UQCR10,MT-CO1,MT-CO3,UQCR11

GOCC:0098803	Respiratory chain complex	0.71	0.0443	COX5A,UQCR10,MT-CO1,MT-CO3,SDHC,NDUFB3,UQCR11
KW-0597	Phosphoprotein	0.14	4.27E-05	TMEM161A,PPP2R5B,RRN3,CDC34,MYH9,FAM118A,AURKA,CKM,CHMP5,TNFAIP1,TUSC2,KALRN,NSRP1,MBD4,HBA2,MRPL34,TERF2,APC,MDM2,BRIP1,KIF13A,MIB1,TRAM1,MAST3,PLD2,ITPKC,ASF1B,MED26,SLC9A1,EHBP1,TUBB4A,ICAM1,LYSMD2,DEF8,PRUNE1,RNF7,MYL9,ZFP36L2,MED7,RASA2,CCNB2,RFX5,CZIB,CCNL1,MON1A,MI-CALL2,VIRMA,DNAAF2,ZNF22,POLR3E,KLF13,DERPC,PGK2,DDX19A,EIF2AK3,MYL1,UTP23,LRRC7,NARF,SEC22A,AQP1,TNKS,CC2D1A,SPHK1,CD46,SSH1,AHCYL2,ZNF609,MYEF2,COX5A,CAMK2G,DEF6,MYO1D,STK11,TUBA3D,ZBTB11,GLRX5,KDELR1,CXorf38,SIRT6,CIR1,PPL,UBE3B,SMARCB1,STAT5A,SYDE1,USP9Y,NBR1,ANP32B,DMPK,MAP3K6,PCNT,G3BP2,PHLDB1,MAP3K3,ZNF318,TSEN15,GNPAT,PLPP3,FRA10AC1,TRAF3IP1,TMEM245,SLC39A7,NCBP1,UPF3A,NMNAT1,CTNNBIP1,APTX,CENPJ,DNAJC19,TSN,DYNLL1,TRAF3,ATN1,DOP1B,ZDHHC20,CBX6,TONSL,ZC3H6,TMEM131L,PLEKHG2,URGCP,PLEKHA8,FAM133B,PPIP5K1,CAPZB,HECTD2,WEE1,MRPL55,CBFB,ZC3H18,YIPF6,RPL7L1,ZBTB20,SMYD3,IQCJ-SCHIP1,PRRC1,PLRG1,INTS8,PTDSS1, JAK3,RNPC3,CCS,HSPA8,PHC1,PACC1,GRAMD1A,RSPRY1,TUBA1C,LDHA,ZNF362,ATXN2,BRF1,SMARCC2,ARPP19,MYL12A,POP4,CHMP2A,SETD1B,FRK,TOX4,ZFAND6,IRAK4,PCGF2,COBLL1,PNISR,MYO1C,SLC35B1,MRTFA,MED19,NIF3L1,CNK3/IPCEF1
KW-0007	Acetylation	0.18	0.0216	MYH9,FAM118A,PFN2,HBA2,TRPC4AP,LSM7,KRAS,LAMTOR5,APC,BRIP1,PNO1,POLQ,LYSMD2,DEF8,PRUNE1,RNF7,MYL9,RASA2,RFX5,VIRMA,ZNF22,PPIB,PGK2,DDX19A,MYL1,RCE1,GLS2,SEC22A,SSH1,AHCYL2,COX5A,DEF6,LYPLA1,MYO1D,STK11,TUBA3D,SIRT6,UBE3B,ANP32B,DYNLRB1,RNF121,GNPAT,ATP5PB,ATP5MD,GCLM,FRA10AC1,UGCG,TMEM245,NCBP1,DNAJC19,TSN,DYNLL1,ATN1,RWDD4,CAPZB,NDUFB3,HRAS,SS18,ZC3H18,YIPF6,SMYD3,TMEM33,PLRG1,PTDSS1,HSPA8,MARVELD1,TMEM258,TUBA1C,LDHA,SMARCC2,TRIAP1,ARPP19,CHMP2A,NUDT3,ZFAND6,IRAK4,ACTB,MYO1C,NIF3L1

KW-0025	Alternative splicing	0.08	0.0216	RHBDD2,TMEM161A,PPP2R5B,RRN3,TCN2,MYH9,FAM118A,ZFYVE21,CDIPT,CHMP5,TNFAIP1,GNB3,PFN2,KALRN,SEC61A1,MBD4,TRPC4AP,TERF2,KRAS,APC,PNPLA8,MDM2,BRIP1,KIF13A,GCHFR,TRAM1,PLD2,MED26,SLC9A1,EHBP1,POLQ,LYSMD2,DEF8,PRUNE1,PNKD,RNF7,SIGMAR1,MYL9,RASA2,MTERF3,RFX5,ARL5A,CCNL1,MON1A,MICALL2,VIRMA,DNAAF2,RPUSD4,POLR3E,SOX7,DDX19A,MYL1,UTP23,LRRC7,KLF7,NARF,GLS2,AQP1,TNKS,CC2D1A,SPHK1,CD46,SSH1,AHCYL2,ZNF609,MYEF2,CAMK2G,LYPLA1,STK11,TTC9C,TUBA3D,TMEM39A,RNF166,KDELR1,CXorf38,UQCR10,EMC10,SIRT6,CIR1,UBE3B,SMARCB1,STAT5A,SYDE1,USP9Y,KLHL7,NBR1,ANP32B,DMPK,OSCP1,DYNLRB1,MAP3K6,NLRC3,PCNT,G3BP2,PHLDB1,RNF121,MAP3K3,ZNF318,TSEN15,GNPAT,SDHC,AP4B1,ARMH3,SLC35A3,GCLM,OMA1,FRA10AC1,DDX31,ERI3,DOLPP1,TOR2A,TRAF3IP1,CSGALNACT2,TMEM245,TAP2,UPF3A,STK19,KIN,APTX,CENPJ,DNAJC19,TSN,GALNT3,TRAF3,SNX11,GPS2,PRPS2,DOP1B,ZDHHC20,TONSL,TMEM131L,RWDD4,MTMR11,PLEKHG2,URGCP,PLEKHA8,FAM133B,PPIP5K1,ZNF780A,CAPZB,HECTD2,WEE1,ZDHHC17,MRPL55,EZH1,NT5DC2,HRAS,SS18,CBFB,ZC3H18,YIPF6,VWA3B,RPL7L1,BTBD9,ZBTB20,SMYD3,TTC26,IQCJ-SCHIP1,PRRC1,LMF2,PLRG1,FAM92A, INTS8,PTDSS1,JAK3,RNPC3,HSPA8,PACC1,GRAMD1A,RSPRY1,LDHA,ATXN2,BRF1,DTX3,SMARCC2,PTGR2,ARPP19,SETD1B,TBC1D7,FRK,NOMO2,TOX4,SLC25A14,ZFAND6,IRAK4,COBLL1,EYA1,PNISR,MYO1C,SLC35B1,MED19,NIF3L1,CNK3/IPCEF1
KW-0505	Motor protein	0.69	0.0216	MYH9,KIF13A,MYL9,MYL1,MYO1D,DYNLRB1,DYNLL1,MYL12A,DNAH11,MYO1C
KW-0963	Cytoplasm	0.14	0.0216	APPBP2,PPP2R5B,CDC34,MYH9,AURKA,CKM,CHMP5,TNFAIP1,PFN2,KALRN,KRAS,LAMTOR5,APC,PNPLA8,MDM2,BRIP1,KIF13A,GCHFR,MIB1,ITPKC,EHBP1,TUBB4A,PRUNE1,PNKD,RNF7,ZFP36L2,RASA2,MICALL2,VIRMA,DNAAF2,SOX7,PGK2,DDX19A,LRRC7,ACOT1,TNKS,CC2D1A,SPHK1,SSH1,AHCYL2,MRFAP1,DEF6,LYPLA1,MYO1D,STK11,TUBA3D,RNF166,ATG101,CIR1,PPL,STAT5A,USP9Y,KLHL7,NBR1,ANP32B,DMPK,DYNLRB1,LAGE3,PFN3,NLRC3,PCNT,G3BP2,ARMH3,TRAF3IP1,NCBP1,U

				PF3A,CTNNBIP1,KIN,APTX,CENPJ,TSN,DYNLL1,TRAF3,GPS2,ATN1,ZDHHC20,TONSL, TMEM131L,BRMS1,URGCP,PPIP5K1,CAPZB,HRAS,VWA3B,SMYD3,IQCJ-SCHIP1, FAM92A,JAK3,CCS,HSPA8,MARVELD1,TUBA1C,LDHA,ATXN2,DTX3, TRIAP1,PTGR2, ARPP19,DNAH11,NUDT3,FRK,ZFAND6,IRAK4,ACTB,EYA1,MYO1C,MRTFA,NIF3L1
KW-0518	Myosin	0.89	0.0225	MYH9,MYL9,MYL1,MYO1D,MYL12A,MYO1C
KW-0813	Transport	0.22	0.0225	APPBP2,TCN2,RP2,CHMP5,SEC61A1,HBA2,KIF13A,TRAM1,SLC9A1,EHBP1,SFXN5,SIG-MAR1,MITD1,DDX19A,SLC5A6,SEC22A,AQP1,TNKS,MYO1D,SMDT1,CNGA2,KDELR1,UQCR10,OSCP1,DYNLRB1,G3BP2,MT-CO1,SDHC,AP4B1,ATP5PB,SLC35A3,SLC39A7, TAP2,NCBP1, UPF3A,DNAJC19,DYNLL1,SNX11,DOP1B,PLEKHA8,NDUFB3,TTC26,SLC35A4,PACC1,GRAMD1A,TRIAP1,UQCR11,CHMP2A,SLC25A14,ZFAND6,SLC35B1
KW-0653	Protein transport	0.36	0.0278	APPBP2,RP2,CHMP5,SEC61A1,KIF13A,TRAM1,EHBP1,DDX19A,SEC22A,TNKS, MYO1D,KDELR1,AP4B1,TAP2,DNAJC19,SNX11,DOP1B,PLEKHA8,TTC26,CHMP2A,ZFAND6,MYO1C
KW-0808	Transferase	0.22	0.0296	CDC34,AURKA,CDIPT,CKM,HAS1,KALRN,MDM2,MIB1,MAST3,ITPKC, POLQ,ALG10,PGK2,EIF2AK3,TNKS,SPHK1,STK11,RNF166,SIRT6,UBE3B,ICMT,DMPK,MAP3K6,MAP3K3,GNPAT,NUS1,UGCG,CSGALNACT2,STK19,NMNAT1,GALNT3, TRAF3,PRPS2,ZDHHC20,PPIP5K1,HECTD2,WEE1,ZDHHC17,EZH1,SMYD3,PRPS1L1,PTDSS1,JAK3,DTX3,SETD1B,FRK,IRAK4
KW-0539	Nucleus	0.12	0.0377	APPBP2,RRN3,CDC34,TNFAIP1,NSRP1,YEATS4,MBD4,LSM7,TERF2,MDM2,BRIP1, GCHFR,ITPKC,ASF1B,MED26,PNO1,POLQ,PRUNE1,PNKD,RNF7,SIGMAR1, ZFP36L2,MED7,RFX5,CCNL1,VIRMA,ZNF22,POLR3E,SOX7,KLF13,DERPC,DDX19A,UTP23,KLF7,NARF,TNKS,CC2D1A,SPHK1,ZNF609,MYEF2,MRFAP1,DEF6,STK11,ZBTB11,SIRT6,CIR1,PPL,SMARCB1,STAT5A,KLHL7,ANP32B,DMPK,LAGE3,PFN3,ZNF318,TSEN15,F

				RA10AC1,DDX31,CHCHD1,NCBP1,UPF3A,STK19,NMNAT1,CTNNBIP1,KIN,APTX,TSN,DYNLL1,GPS2,ATN1,CBX6,TONSL,BRMS1,URGCP,ZNF780A,WEE1,EZH1,HRAS,SS18,CBFB,ZC3H18,ZBTB20,SMYD3,TMEM33,PLRG1,FAM92A,INTS8,RNPC3,RBM4B,HSPA8,PHC1,MARVELD1,ZNF362,BRF1,SMARCC2,TGIF2LX,POP4,SETD1B,FRK,TOX4,PCGF2,ACTB,EYA1,PNISR,MYO1C,MRTFA,MED19,NIF3L1
KW-0547	Nucleotide-binding	0.22	0.048	CDC34,MYH9,AURKA,RP2,CKM,KALRN,KRAS,BRIP1,KIF13A,MAST3,ITPKC,TUBB4A,POLQ,ARL5A,PGK2,DDX19A,EIF2AK3,SPHK1,CAMK2G,MYO1D,STK11,TUBA3D,CNGA2,DMPK,MAP3K6,NLRC3,MAP3K3,DDX31,TOR2A,TAP2,STK19,NMNAT1,PRPS2,PPIP5K1,WEE1,HRAS,PRPS1L1,JAK3,HSPA8,TUBA1C,DNAH11,FRK,IRAK4,ACTB,MYO1C
KW-0067	ATP-binding	0.24	0.0496	CDC34,MYH9,AURKA,CKM,KALRN,BRIP1,KIF13A,MAST3,ITPKC,POLQ,PGK2,DDX19A,EIF2AK3,SPHK1,CAMK2G,MYO1D,STK11,DMPK,MAP3K6,NLRC3,MAP3K3,DDX31,TOR2A,TAP2,STK19,NMNAT1,PRPS2,PPIP5K1,WEE1,PRPS1L1,JAK3,HSPA8,DNAH11,FRK,IRAK4,ACTB,MYO1C
KW-0112	Calmodulin-binding	0.58	0.0496	MYH9,ITPKC,SLC9A1,SPHK1,CAMK2G,MYO1D,CNGA2,PCNT,MYO1C
KW-0418	Kinase	0.33	0.0496	AURKA,CKM,KALRN,MAST3,ITPKC,PGK2,EIF2AK3,SPHK1,CAMK2G,STK11,DMPK,MAP3K6,MAP3K3,STK19,PRPS2,PPIP5K1,WEE1,PRPS1L1,JAK3,FRK,IRAK4
KW-0496	Mitochondrion	0.26	0.0496	ALKBH7,MRPL34,SFXN5,PNKD,MTERF3,MRPL17,RPUSD4,GLS2,COX5A,STK11,SMDT1,GLRX5,UQCR10,PPL,DMPK,MT-CO1,MT-CO3,SDHC,ATP5PB,ATP5MD,OMA1,CHCHD1,DNAJC19,MRPL2,DYNLL1,TRAF3,GPS2,TMEM177,MRPL55,NDUFB3,TRIAP1,UQCR11,SLC25A14
KW-0999	Mitochondrion inner membrane	0.45	0.0496	COX5A,SMDT1,UQCR10,MT-CO1,MT-CO3,SDHC,ATP5PB,OMA1,DNAJC19,TMEM177,NDUFB3,UQCR11,SLC25A14

GO:0044 237	Cellular metabolic process	0.16	0.0031	RHBDD2,APPBP2,PPP2R5B,RRN3,CDC34,TCN2,AURKA,CDIPT,CKM,HAS1,CHMP5, TNFAIP1,KALRN,ALKBH7,NSRP1,MBD4,HBA2,TRPC4AP,MRPL34,LSM7,TERF2,APC, PNPLA8,MDM2,BRIP1,GCHFR,MIB1,MAST3,PLD2,ITPKC,MED26,POLQ,ALG10,PNKD, RNF7, ZFP36L2,MED7,MRPL17,CCNL1,VIRMA,RPUSD4,POLR3E,LDHAL6B,KLF13, PGK2,EIF2AK3,UTP23,RCE1,GLS2,ACOT1,TNKS,SPHK1,SSH1,AHCYL2,COX5A, CAMK2G,LYPLA1,STK11,TMEM39A,RNF166,GLRX5,UQCR10,SIRT6,ATG101,CIR1, UBE3B,SMARCB1,STAT5A,USP9Y,NBR1,ICMT,DMPK,LAGE3,MAP3K6,MT-CO1,RNF121, MAP3K3,MT-CO3,TSEN15,GNPAT,SDHC,NUS1,ATP5PB, ATP5MD,SLC35A3,GCLM, PLPP3, FRA10AC1,ERI3,DOLPP1,CHCHD1,PKIG,UGCG,CSGALNACT2,NCBP1,UPF3A, STK19,NMNAT1,KIN,APTX,MRPL2,TSN,GALNT3,ATN1,PRPS2,ZDHHC20,TONSL,MTM R11,PPIP5K1,WEE1,ZDHHC17,MRPL55,EZH1,NDUFB3,HRAS,CBFB,RPL7L1,BTBD9,SMY D3,PLRG1,PRPS1L1,INTS8,PTDSS1,JAK3,RNPC3,RBM4B,CCS,HSPA8,GRAMD1A,TMEM2 58,LDHA,ATXN2,BRF1,SMARCC2,PTGR2,POP4,UQCR11,CHMP2A,SETD1B,NUDT3,FRK, SLC25A14,IRAK4,EYA1,MED19

A

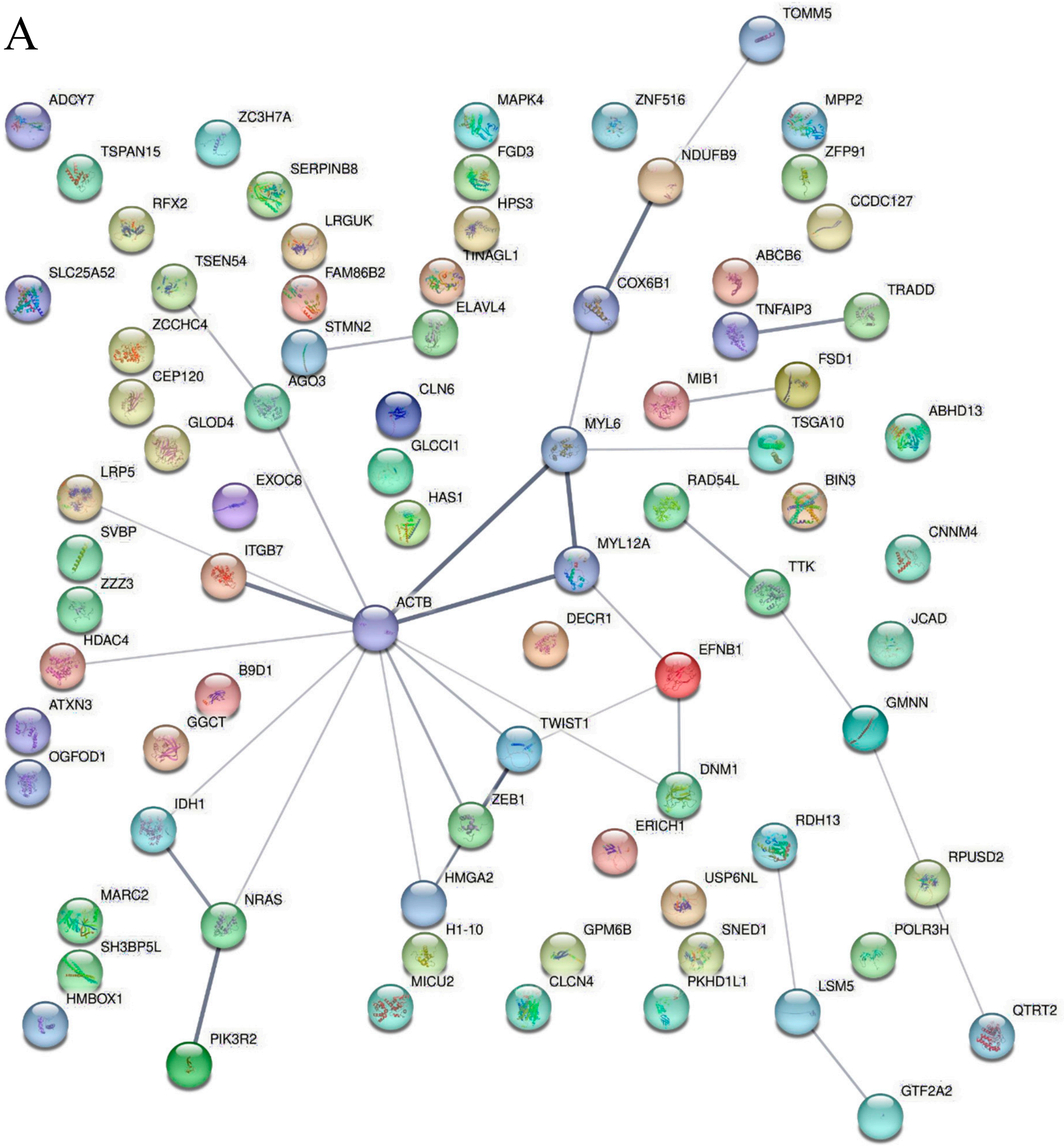
TOMM5
ADCY7
ZC3H7A
MAPK4
ZNF516
MPP2
TSPAN15
SERPINB8
FGD3
NDUFB9
ZFP91
RFX2
LRGUK
HPS3
CCDC127
TINAGL1
ABCB6
SLC25A52
TSEN54
FAM86B2
COX6B1
TRADD
ELAVL4
TNFAIP3
ZCCHC4
STMN2
FSD1
CEP120
AGO3
CLN6
MIB1
GLOD4
GLCCI1
MYL6
TSGA10
ABHD13
LRP5
EXOC6
RAD54L
BIN3
HAS1
SVBP
ITGB7
MYL12A
CNNM4
ZZZ3
ACTB
TTK
HDAC4
DECR1
JCAD
EFNB1
B9D1
GMNN
ATXN3
GGCT
TWIST1
OGFOD1
DNM1
IDH1
ZEB1
ERICH1
RDH13
RPUSD2
HMGA2
USP6NL
MARC2
NRAS
SH3BP5L
H1-10
GPM6B
SNED1
POLR3H
HMBOX1
MICU2
CLCN4
PKHD1L1
LSM5
QTRT2
PIK3R2
GTF2A2

B

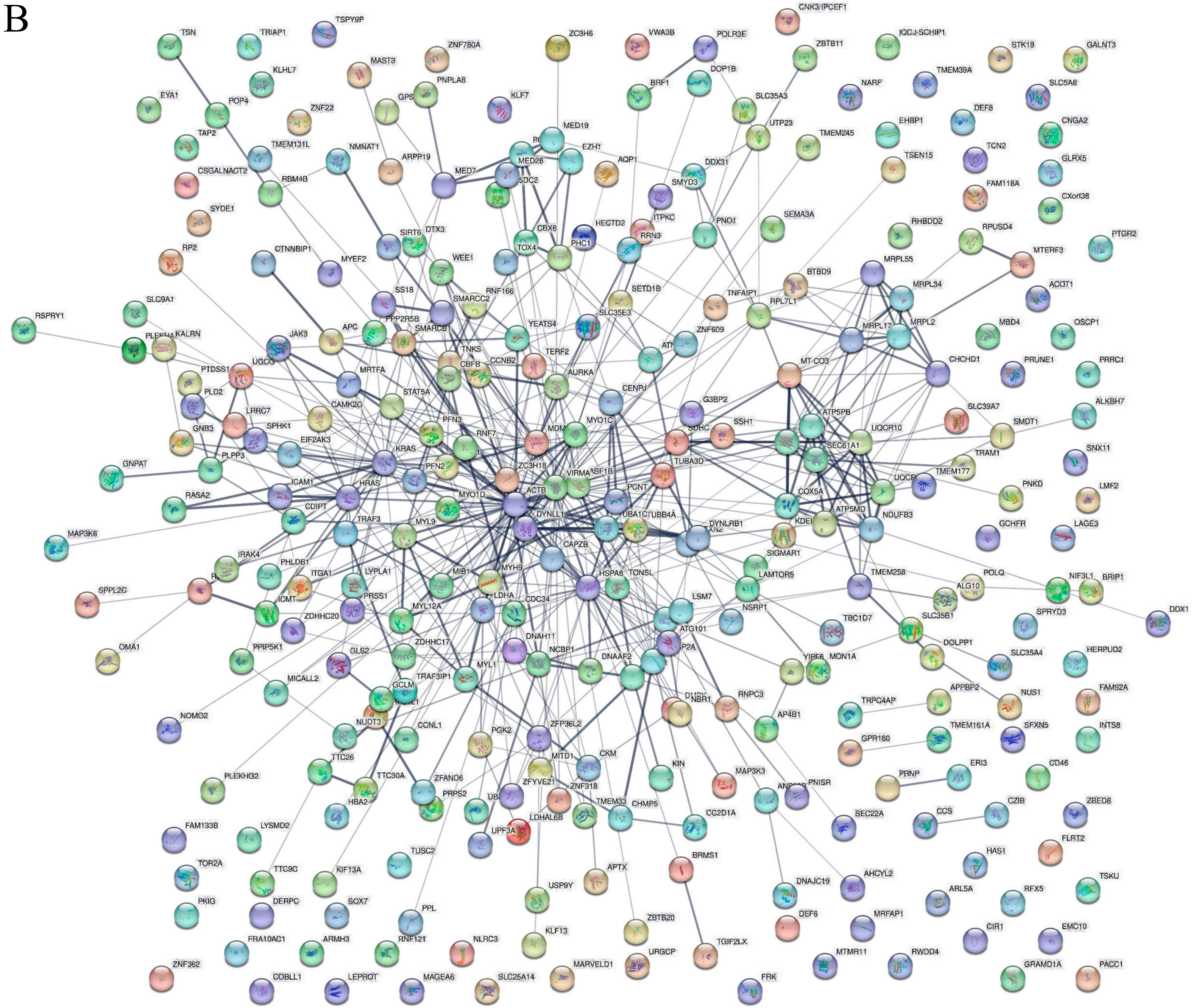
CNK3/IPCEF1
TSN
TRIAP1
TSPY9P
ZC3H6
VWA3B
POLR3E
ZBTB11
IQCJ-SCHIP1
STK19
GALNT3
ZNF780A
MAST3
KLHL7
PNPLA8
BRF1
DOP1B
NARF
TMEM39A
SLC5A6
EYA1
POP4
ZNF22
GPS
KLF7
SLC35A3
DEF8
CNGA2
TAP2
TMEM131L
NMNAT1
MED19
UTP23
TMEM245
EHBP1
TCN2
CSGALNACT2
RBM4B
ARPP19
MED7
MED26
EZH1
AQP1
DDX31
SMYD3
TSEN15
GLRX5
FAM118A
SYDE1
CXorf38
ITPKC
PNO1
SEMA3A
RHBDD2
RPUSD4
HECTD2
RRN3
SIRT6
DTX3
CBX6
TOX4
PHC1
PTGR2
RP2
CTNNBIP1
MYEF2
WEE1
BTBD9
MRPL55
MTERF3
SETD1B
SS18
RNF166
SMARCC2
TNFAIP1
RPL7L1
MRPL34
ACOT1
SLC9A1
SLC35E3
MRPL17
MRPL2
RSPRY1
JAK3
APC
PPP2R5B
SMARCB1
YEATS4
ZNF609
MBD4
OSCP1
KALRN
UGCG
TNKS
CCNB2
TERF2
MT-CO3
CHCHD1
PRUNE1
PRRC1
PTDSS1
MRTFA
CBFB
AURKA
PLD2
STAT5A
CENPJ
LRRC7
CAMK2G
G3BP2
ALKBH7
SLC39A7
GNB3
SPHK1
PFN3
SSH1
ATP5PB
SMDT1
RNF7
SDHC
UQCR10
EIF2AK3
KRAS
ZC3H18
SEC61A1
SNX11
PLPP3
TUBA3D
TRAM1
GNPAT
PFN2
VIRMA
PCNT
TMEM177
ICAM1
HRAS
ACTB
COX5A
PNKD
LMF2
RASA2
MYO1D
ATP5MD
CDIPT
DYNLL1
NDUFB3
TRAF3
TUBB4A
DYNLRB1
GCHFR
MAP3K6
MYL9
CAPZB
LAGE3
SIGMAR1
IRAK4
PHLDB1
TMEM258
POLQ
LAMTOR5
NIF3L1
BRIP1
ITGA1
LYPLA1
HSPA8
TONSL
MIB1
MYH9
ALG10
SPPL2C
LDHA
LSM7
DDX1
CDC34
NSRP1
SPRYD3
ICMT
PRSS1
MYL12A
TBC1D7
SLC35B1
ZDHHC20
ATG101
OMA1
DNAH11
DOLPP1
HERPUD2
PPIP5K1
ZDHHC17
NCBP1
SLC35A4
GLS2
DNAAF2
YIPF6
MON1A
MICALL2
TRAF3IP1
MYL1
GCLM
NBR1
RNPC3
APPBP2
FAM92A
TRPC4AP
NUS1
NOMO2
AP4B1
NUDT3
CCNL1
PGK2
ZFP36L2
TMEM161A
SFXN5
INTS8
GPR180
MITD1
CKM
TTC26
KIN
MAP3K3
ERI3
CD46
PLEKHG2
TTC30A
ZFAND6
ZFYVE21
ZNF318
PNISR
PRNP
HBA2
PRPS2
TMEM33
CHMP5
CZIB
ZBED8
FAM133B
LDHAL6B
CC2D1A
SEC22A
CCS
LYSMD2
UPF3A
FLRT2
TUSC2
BRMS1
HAS1
TOR2A
KIF13A
TTC9C
APTX
AHCYL2
ARL5A
TSKU
DNAJC19
RFX5
PKIG
DERPC
SOX7
USP9Y
PPL
ZBTB20
DEF6
MRFAP1
CIR1
EMC10
FRA10AC1
ARMH3
RNF121
NLRC3
KLF13
TGIF2LX
MTMR11
RWDD4
URGCP
ZNF362
MARVELD1
GRAMD1A
PACC1
COBLL1
LEPROT
MAGEA6
SLC25A14
FRK

Figure S1. Identity and interaction of proteins within functional network that are altered 24h after conditioned medium transfer in chondrocyte and endothelial cells. STRING database was used to illustrate the interactions of the proteins in chondrocyte T/C-28A2 cells and EA.hy926 that received conditioned medium from proton exposed SW1353 at doses 0.1 Gy and 2 Gy respectively. (**A**) Proteins altered in T/C-28A2 after receiving medium from SW1353 exposed to 0.1 Gy are associated to cytoskeleton and actin dynamics pathway. Hub proteins in the network: ACTB, TWIST1, HMGA2, ZEB1, MYL12A, EFNB1, MYL6, DNM1, NDUFB9 and TTK. (**B**) Proteins altered in EA.hy926 after receiving medium from SW1353 exposed to 2 Gy are associated with mitochondrial energy metabolism and oxidative phosphorylation, neurodegenerative disorders (such as Alzheimer's, Parkinson's, and Huntington's disease), chromatin-remodeling complexes (including npBAF and bBAF), and autophagy pathways. Hub proteins in the network: UQCR10, UQCR11, COX5A, NDUFB3, SDHC, MT-CO1, MT-CO3, ACTB, ATP5PB and KRAS. Nodes represent individual proteins, and edges denote functional or physical associations between them
